# The global burden and trends analysis of early-onset colorectal cancer attributable to dietary risk factors in 204 countries and territories, 1990–2019: a secondary analysis for the global burden of disease study 2019

**DOI:** 10.3389/fnut.2024.1384352

**Published:** 2024-05-31

**Authors:** Jiao Su, Yuanhao Liang, Xiaofeng He

**Affiliations:** ^1^Department of Biochemistry, Changzhi Medical College, Changzhi, China; ^2^Clinical Experimental Center, Jiangmen Key Laboratory of Clinical Biobanks and Translational Research, Jiangmen Central Hospital, Jiangmen, China; ^3^Institute of Evidence-Based Medicine, Heping Hospital Affiliated to Changzhi Medical College, Changzhi, China

**Keywords:** early-onset, colorectal cancer, burden of disease, dietary risks, age-specific rate, estimated annual percentage change

## Abstract

**Background:**

Rising trends in early-onset colorectal cancer (CRC) burden have been observed, but the distribution and temporal patterns of early-onset CRC attributable to dietary risks remain unclear.

**Objectives:**

This study aimed to estimate the burden of early-onset CRC attributable to dietary risk factors globally, regionally, and nationally, by age and sex, from 1990 to 2019.

**Methods:**

The absolute number and age-specific rates (ASR) of diet-related early-onset CRC burden, as well as summary exposure value (SEV) of attributable dietary risk factors, were extracted from the Global Burden of Disease (GBD) Study 2019. The temporal changes in the burden between 1990 and 2019 were analyzed by calculating the percentage change in the absolute number of burden and the estimated annual percentage change (EAPC) in ASR of burden. The annualized rates of change (ARC) were calculated to evaluate the variation trend of SEV.

**Results:**

In 2019, diet-related early-onset CRC caused 30,096 (95% UI: 23,148 to 36,091) death cases and 1,465,755 (95% UI: 1,126,489 to 1,761,661) DALYs worldwide, accounting for 34.8% deaths and 34.4% DALYs of overall early-onset CRC, respectively. Moreover, a diet low in milk (responsible for 16.5% [95% UI: 11.1 to 21.9%] of DALYs in 2019), low in whole grains (15.2% [95% UI: 5.9 to 19.9%]), low in calcium (14.3% [95% UI: 10.7 to 18.9%]), high in red meat (5.3% [95% UI: 1.7 to 9.5%]), high in processed meat (2.5% [95% UI: 0.9 to 4.0%]), and low in fiber (2.3% [95% UI: 0.9 to 4.2%]) were early-onset CRC attributable dietary risk factors. The age-specific DALYs rate of early-onset CRC attributable to each dietary risk factor generally showed an increasing trend globally between 1990 and 2019, except for low intake of fiber (EAPC = −0.57, 95% CI: −0.76 to −0.38). In addition, from 1990 to 2019, males have a higher burden than females and this gap may continue to widen due to the increasing difference between the sexes in most dietary risk factors. Furthermore, dietary risks-attributable early-onset CRC burden has shifted from regions with high socio-demographic index (SDI) to high-middle and middle SDI quintiles with uncontrolled dietary risks.

**Conclusion:**

Early-onset CRC remains a concerning issue globally, and effective prevention and modification of dietary risk factors holds great promise to reduce early-onset CRC-related burden. Prioritizing diet improvement for males is critical and urgent for CRC control efforts, particularly for those living in developing countries with ongoing dietary pattern transition.

## 1 Introduction

Cancer is a major contributor to the global burden of disease and the cancer-related burden is projected to reach 28.4 million cases in 2040 worldwide, being a significant public health concern (1). Colorectal cancer (CRC) is a highly common cancer and ranked as the third most common cause of cancer-related deaths and the second leading cause of disability-adjusted life-years (DALYs) for cancer across the globe in 2019 (2). The number of incident cases and deaths from CRC worldwide has more than doubled from 1990 to 2019 (2). In addition, the incidence of CRC varies significantly by geographical region, with high incidence in high-income countries and low incidence in low-income countries (1, 2). However, over the past 30 years, the temporal patterns of CRC incidence either remained unchanged or decreased in high socio-demographic index (SDI) countries, whereas low-income and economically transitioning countries have witnessed an upward trend (2, 3). CRC is associated with aging, and the burden of CRC increases sharply after the age of 50 (4). However, the incidence and mortality of early-onset CRC, defined as cancer cases diagnosed in adults younger than 50 years, has alarmingly increased across the globe particularly in high-income countries over the last decades (2, 5, 6). The incidence of early-onset CRC in the USA has been on the rise and it is projected to nearly double by 2030 (7). CRC incidence has increased among aged 20-to-49-years-old in Europe over the past 25 years, with the fastest rise occurring in individuals aged 20–39 years (8). In Australia, the incidence of CRC surged among individuals diagnosed below the age of 40 but remained stable or declining in adults over 40 years between 1990 and 2010 (9). Moreover, from 1990 to 2019, CRC incidence rose from 3.9 to 10.1 per 10^5^ populations in East Asia and increased by 104% in Latin America and the Caribbean among individuals aged 15–49 years (10). Importantly, early-onset CRC exhibits distinct clinical and molecular characteristics, including higher rates of synchronous metastatic disease, microsatellite instability, distal/rectal tumor location, and the CMS1 subtype, with fewer BRAF V600 mutations compared to patients over 50. Furthermore, patients aged 18–29 and those with predisposing conditions show differences within the broader early-onset CRC group, suggesting it may not be appropriate to consider all CRC patients under 50 as a single group (11). In summary, the epidemiological patterns of CRC have changed broadly over the past decades and the rapid increase of early-onset CRC has become a growing concern.

With the population growing and people living longer, the increasing incidence of early-onset CRC poses a significant challenge to public health systems. The upward trend in early-onset CRC is expected to persist worldwide unless precise preventative measures are implemented. Although genetic factors predispose individuals to early-onset CRC, most CRC cases are sporadic and largely attributable to unhealthy dietary patterns and lifestyles (12, 13). For instance, modifiable risk factors account for 45% of CRC cases in the UK and 47% in the USA (12). Additionally, increases in incidence by birth cohort indicate that early-life exposure to modifiable risk factors is an important contributor to early-onset CRC (10). Moreover, it was reported that early-onset CRC are more aggressive tumors with worse prognosis compared to later-onset CRC, so identifying potential risk factors is important for improving the prevention and therapy of early-onset CRC (14, 15). The significant association between unhealthy dietary habits and the risk of CRC has been well-established by extensive epidemiologic and experimental studies (16). It was acknowledged that a higher consumption of red meat and processed meat was associated with a higher risk of CRC, while a high intake of whole grains, fiber, and milk contributed to a decreased risk. Notably, between 1990 and 2010, the increase in global consumption of unhealthy food exceeded the rise in consumption of healthy food (17). In 2019, it was reported that 32% of CRC-related deaths and 34% of CRC-related DALYs burden were due to dietary risks, surpassing tobacco smoking- and alcohol use-attributable CRC burden (18). Furthermore, a diet low in milk was the greatest contributor to the global burden of CRC in 2019, responsible for 15.6% of CRC-related DALYs (2). It was reported that younger adults had poorer dietary patterns than older adults (17). However, the effect of dietary risk factors on the burden of early-onset CRC has not been systematically evaluated and needs further exploration. To effectively prevent expected increases in the global burden of early-onset CRC, timely estimates of dietary risks-attributable early-onset CRC burden and its temporal trends are essential at the global, regional, and national levels to guide evidence-based dietary strategies, planning, and resource allocation for CRC prevention.

The Global Burden of Disease (GBD) study 2019 systematically assesses the incidence, prevalence, mortality, and resulting health loss of 369 diseases and injuries, along with 87 attributable risk factors across 204 countries and territories (19). Gu and colleagues have evaluated the overall burden of early-onset CRC and its risk factors from 1990 to 2019, while the other study described the burden of dietary risks-related CRC for all ages (20, 21). However, no study has specifically assessed the global burden of early-onset CRC attributable to dietary risk factors and its secular trend, as well as the variations by gender and between regions or countries with different levels of socio-economic development. Therefore, using data from the GBD study 2019, our study aims to methodically analyze the global, regional, and national burden of early-onset CRC attributed to dietary risks, as well as its temporal trends from 1990 to 2019. Meanwhile, this investigation takes into account regional, temporal, gender-based, and socio-economic development differences.

## 2 Methods

### 2.1 Data source and data collection

The GBD study, a collaborative project led by the Institute for Health Metrics and Evaluation (IHME), aims to identify, compile, and standardize the impact of diseases, injuries, and risk factors on health loss across various age groups, genders, and geographic regions at specific time points. Since 1990, this evaluation has been conducted annually, using a standardized approach to produce regular estimates of disease burden. The GBD study 2019 provides insights into age-specific and sex-specific estimations of incidence, prevalence, mortality, years of life lost (YLLs), years lived with disability (YLDs), and DALYs for 369 diseases and injuries, as well as 87 risk factors, across 204 countries and territories (19). DALYs are a measure that combines the years lost due to premature death (YLLs) and the years lost in productive life due to disability (YLDs) compared to a standardized life expectancy (22). This measure helps assess disease burden systematically, covering the cumulative number of years lost to ill health, disability, or untimely death. Detailed information about the GBD study protocol can be found online.^1^ We obtained yearly data on dietary-attributable CRC deaths and DALY burden in young adults under 50 from 1990 to 2019 across the globe, 5 socio-demographic index quintiles, 21 GBD regions, and 204 countries and territories using the Global Health Data Exchange (GHDx) query tool^2^ in the current study.

### 2.2 Case identification and exposure definition

We used age at diagnosis or death before 50 years as a proxy to classify early-onset CRC, as early-onset cancers are often defined as those diagnosed in adults under 50 years of age (23). In the GBD study 2019, the list of international classification of diseases (ICD) codes mapped to the global burden of disease for CRC has been detailly described in previous studies (2). Dietary risks are a composite risk factor consisting of suboptimal exposure to multiple dietary factors. Exposure for each dietary factor was defined as either not reaching or exceeding the theoretical minimum risk exposure level (TMREL) at which the minimum risk occurs (24). The estimation of the population attributable fraction (PAF) for combinations of dietary risk factors was adjusted to account for the overestimation of the combined effects, using a mediation matrix, as the impact of an individual dietary factor on early-onset CRC can be mediated through an intermediate factor (24). The GBD study 2019 identified 6 dietary risk factors for early-onset CRC among the 87 risk factors assessed: (1) diets low in milk (< 360–500 g per day); (2) diets low in whole grains (< 140–160 g per day); (3) diets low in calcium (< 1.06–1.1 g per day); (4) diet high in red meat (any intake of red meat per day); (5) diet high in processed meat (any intake of all processed meats per day); and (6) diet low in fiber (< 21–22 g per day) (19, 24). Dietary risks-related burden was estimated in the GBD study 2019 using a comparative risk assessment framework. Input data sources mainly come from nutrition surveys, such as the 24-h diet recall, the food frequency questionnaire, and the Food and Agriculture Organization of the United Nations (24).

### 2.3 Socio-demographic index and human development index

Researchers of the GBD study have devised and established the SDI metric, as a composite measure of a country’s development, based on three pivotal indicators: (1) the total fertility rate for individuals under the age of 25, (2) the average educational attainment for those aged 15 and above, and (3) the distribution of income *per capita* with temporal lag. These indicators serve to exhibit a strong correlation with health outcomes. Based on the calculated SDI score, regions and countries are classified into five distinct quintiles: high SDI, high-middle SDI, middle SDI, low-middle SDI, and low SDI. Another frequently employed index for evaluating human development levels is the Human Development Index (HDI), which can be accessed through the United Nations Development Program.^3^ The HDI encompasses three fundamental dimensions: (1) life expectancy, (2) accessibility of education and literacy, and (3) living conditions and income. The HDI is categorized into four levels: very high human development (0.8–1.0), high human development (0.7–0.79), medium human development (0.55–0.70), and low human development (below 0.55). This classification facilitates the comparison and benchmarking of countries’ progress in terms of life expectancy, education, literacy, and overall living conditions (25).

### 2.4 Summary exposure value

In the GBD study 2019, summary exposure value (SEV) is reported as the risk-weighted prevalence of dietary risk factors attributable to early-onset CRC (24). SEV ranges from 0 to 100, where 0 indicates that a population has the lowest exposure to dietary risk factors, while 100 indicates the population has the highest risk exposure. The temporal change of SEV was measured by annualized rates of change (ARC). An increase in SEV indicates an increase in exposure to dietary risk factors in the population, and vice versa.

### 2.5 Statistical analysis

In this study, the age-specific rate (ASR) and estimated annual percentage change (EAPC) in ASR were utilized to assess the epidemiologic patterns of dietary risks related to early-onset CRC (26). EAPC was widely used to quantify and summarize the temporal change of ASR (27). A regression line was fitted to the natural logarithm of the rates, denoted as y=α+βx+ε , where $y=\ln\left(ASR\right)$ , and x=calendaryear . The EAPC was calculated as $100\times \left(\exp\left(\beta \right)-1\right)$ , and the corresponding 95% confidence interval (CI) could be obtained from the linear regression model. If the EAPC value and the lower limits of its 95% CI were both greater than 0, the ASR was considered to show an increasing trend. Conversely, if both were less than 0, a decreasing trend was reported.

Population-attributable fractions (PAF) were calculated for each dietary risk factor using the intake of each dietary factor, the estimated relative risk, and the level of intake associated with the lowest risk. The burden attributable to dietary factors was then estimated as the PAF multiplied by the overall burden measure of early-onset CRC. Furthermore, we used Pearson correlation analysis to examine the relationship between EAPCs, ASRs, and HDI scores (in 2019) to understand the factors influencing the epidemiologic patterns of early-onset CRC. All statistics Data analysis was done using R software, version 4.1.0 (R Foundation for Statistical Computing). A two-tailed *p* < 0.05 was considered statistically significant.

## 3 Results

### 3.1 The distribution and trend of dietary risks-related early-onset colorectal cancer deaths burden

**Figure 1:**
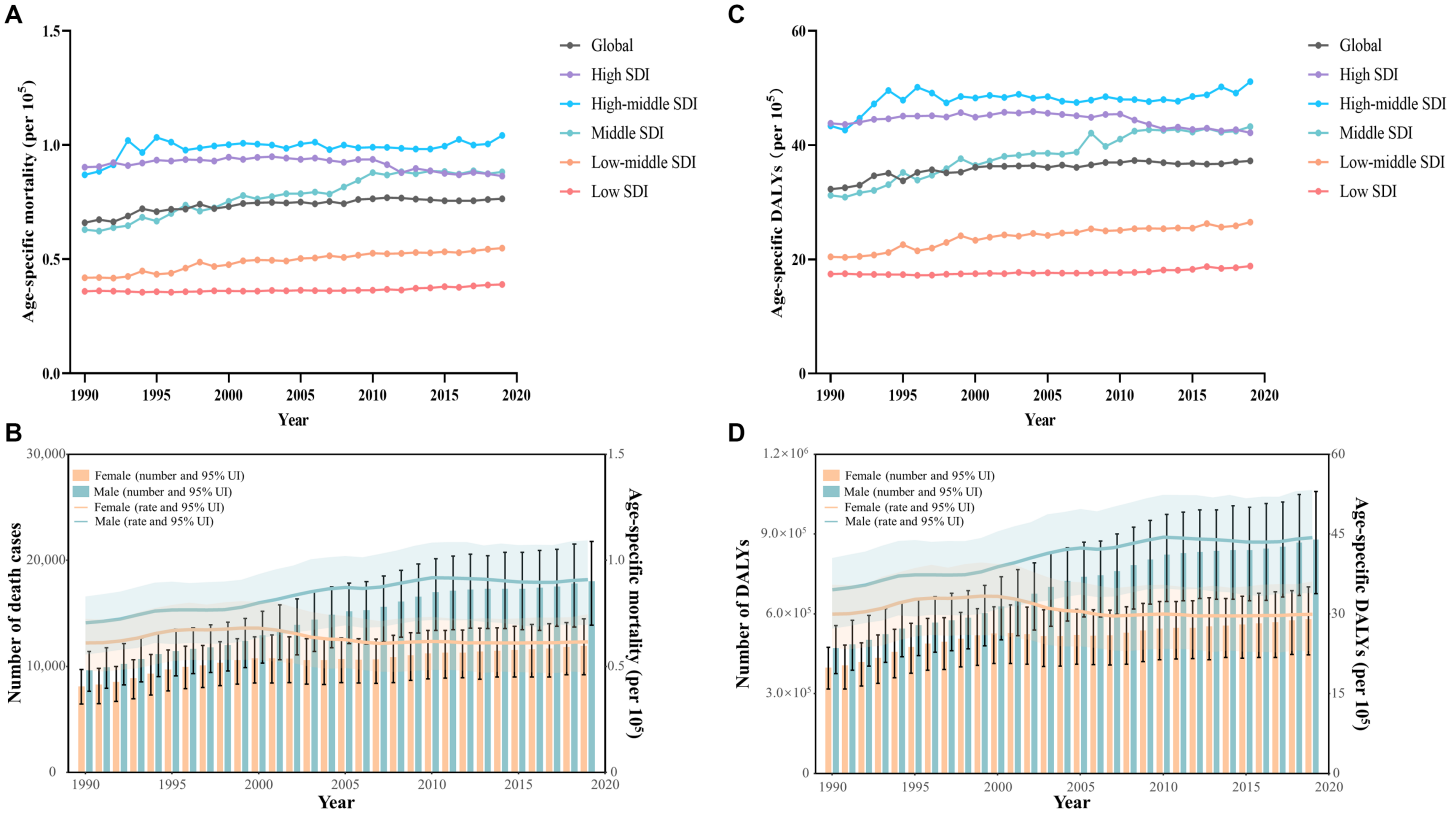
Early-onset colorectal cancer (CRC) burden attributable to dietary risks by SDI quintiles and by sex, from 1990 to 2019. **(A)** Age-specific mortality rate for early-onset CRC due to dietary risks by 5 SDI regions. **(B)** The global number of death cases and age-specific mortality rate for early-onset CRC due to dietary risks by sex. **(C)** Age-specific DALYs rate for early-onset CRC due to dietary risks by 5 SDI regions. **(D)** The global number of DALYs and age-specific DALYs rate for early-onset CRC due to dietary risks by sex.

Globally, there was a 68.2% increase in the number of dietary risks-related early-onset CRC death cases, rising from 17,897 (95% uncertainty interval [UI]: 14,148 to 20,955) in 1990 to 30,096 (95% UI: 23,148 to 36,091) in 2019. The overall age-specific mortality rate exhibited a yearly increase of 0.39 (95% confidence interval [CI]: 0.30 to 0.47) during the same period, reaching 0.76 (95% UI: 0.59 to 0.92) per 10^5^ populations in 2019 (Table 1). In 2019, the highest age-specific mortality rate was observed in high-middle SDI regions (1.04 per 10^5^, 95% UI: 0.77 to 1.28 per 10^5^) (Table 1; Figure 1A). Furthermore, the age-specific mortality rate tends to trend upward across SDI areas (barring the high SDI quintiles), with the most significant increase in middle SDI areas (EAPC = 1.30, 95% CI: 1.18 to 1.42) (Table 1; Figure 1A). Additionally, between 1990 and 2019, Central Latin America experienced the largest increase in the age-specific mortality rate of early-onset CRC due to dietary risks (EAPC = 2.00, 95% CI: 1.94 to 2.06), followed by East Asia (EAPC = 1.84, 95% CI: 1.70 to 1.98) and Tropical Latin America (EAPC = 1.38, 95% CI: 1.16 to 1.59) (Table 1).

**Table 1:** Early-onset colorectal cancer-related deaths burden attributable to dietary risks in 1990 and 2019, and its temporal trends from 1990 to 2019.

Characteristics	1990		2019		1990 ~ 2019	
	Deaths No. (95% UI)	ASR per 100,000 No. (95% UI)	Deaths No. (95% UI)	ASR per 100,000 No. (95% UI)	Percent change in absolute number (%)	EAPC No. (95% CI)
Overall	17,897 (14,148–20,955)	0.66 (0.52–0.77)	30,096 (23,148–36,091)	0.76 (0.59–0.92)	68.2	0.42 (0.33–0.52)
Sex						
Male	9,706 (7,651–11,408)	0.71 (0.56–0.83)	18,095 (13,870–21,779)	0.91 (0.70–1.09)	86.4	0.92 (0.79–1.06)
Female	8,191 (6,454–9,687)	0.61 (0.48–0.72)	12,001 (9,201–14,500)	0.62 (0.47–0.75)	46.5	−0.25 (−0.40−−0.11)
Socio-demographic index						
High	3,884 (2,947–4,647)	0.90 (0.69–1.08)	4,061 (3,039–4,865)	0.86 (0.65–1.04)	4.6	−0.19 (−0.29−−0.09)
High -middle	5,268 (4,097–6,232)	0.87 (0.68–1.03)	7,597 (5,609–9,341)	1.04 (0.77–1.28)	44.2	0.23 (0.08–0.37)
Middle	5,625 (4,584–6,574)	0.62 (0.51–0.73)	11,193 (8,783–13,585)	0.89 (0.70–1.08)	99.0	1.30 (1.18–1.42)
Low-middle	2,268 (1,815–2,693)	0.42 (0.34–0.50)	5,123 (3,889–6,183)	0.55 (0.42–0.66)	125.9	0.95 (0.85–1.04)
Low	843 (652–1,050)	0.36 (0.28–0.45)	2,105 (1,630–2,529)	0.39 (0.30–0.47)	149.7	0.24 (0.18–0.30)
GBD regions						
High-income Asia Pacific	925 (691–1,128)	1.00 (0.74–1.21)	730 (559–877)	0.90 (0.69–1.08)	−21.1	−0.48 (−0.59−−0.37)
Central Asia	294 (225–349)	0.88 (0.67–1.04)	350 (257–427)	0.72 (0.53–0.88)	19.0	−1.43 (−1.66−−1.19)
East Asia	5,635 (4,378–6,809)	0.82 (0.63–0.99)	9,944 (7,267–12,605)	1.33 (0.97–1.69)	76.5	1.84 (1.70–1.98)
South Asia	1,578 (1,234–1,921)	0.30 (0.23–0.36)	3,765 (2,778–4,731)	0.39 (0.29–0.49)	138.6	0.84 (0.75–0.93)
Southeast Asia	1,722 (1,389–2,060)	0.73 (0.59–0.87)	4,119 (3,181–5,097)	1.14 (0.88–1.41)	139.2	1.41 (1.25–1.56)
Australasia	117 (90–140)	1.08 (0.84–1.30)	124 (94–151)	0.92 (0.69–1.12)	6.0	−0.60 (−0.69−−0.51)
Caribbean	105 (79–126)	0.58 (0.44–0.69)	184 (129–239)	0.77 (0.54–1.00)	75.2	0.95 (0.88–1.02)
Central Europe	681 (492–838)	1.12 (0.81–1.37)	597 (411–760)	1.13 (0.78–1.44)	−12.3	−0.39 (−0.54−−0.23)
Eastern Europe	1,288 (963–1,554)	1.17 (0.87–1.41)	1,104 (784–1,408)	1.13 (0.80–1.44)	−14.3	−1.36 (−1.83−−0.88)
Western Europe	1,766 (1,325–2,119)	0.91 (0.69–1.10)	1,464 (1,097–1,763)	0.77 (0.58–0.92)	−17.1	−0.72 (−0.90−−0.53)
Andean Latin America	72 (57–87)	0.38 (0.31–0.47)	174 (121–233)	0.52 (0.36–0.70)	141.7	1.19 (0.99–1.38)
Central Latin America	279 (214–331)	0.34 (0.26–0.41)	791 (560–1,018)	0.60 (0.42–0.77)	183.5	2.00 (1.94–2.06)
Southern Latin America	239 (187–279)	0.97 (0.76–1.14)	398 (307–480)	1.17 (0.90–1.41)	66.5	0.64 (0.57–0.71)
Tropical Latin America	421 (323–502)	0.54 (0.41–0.64)	962 (725–1,177)	0.81 (0.61–0.99)	128.5	1.38 (1.16–1.59)
North Africa and Middle East	736 (541–948)	0.45 (0.33–0.58)	1,823 (1,293–2,290)	0.55 (0.39–0.69)	147.7	0.54 (0.27–0.82)
High-income North America	1,218 (914–1,467)	0.82 (0.61–0.99)	1,508 (1,108–1,838)	0.90 (0.66–1.10)	23.8	0.45 (0.3–0.6)
Oceania	16 (11–20)	0.5 (0.36–0.64)	41 (29–56)	0.61 (0.43–0.83)	156.3	0.73 (0.7–0.76)
Central Sub-Saharan Africa	88 (64–118)	0.36 (0.26–0.48)	233 (157–319)	0.38 (0.25–0.51)	164.8	0.08 (−0.09–0.24)
Eastern Sub-Saharan Africa	320 (239–407)	0.39 (0.29–0.49)	826 (605–1,052)	0.42 (0.30–0.53)	158.1	0.15 (−0.01–0.31)
Southern Sub-Saharan Africa	148 (120–177)	0.57 (0.46–0.68)	278 (215–344)	0.66 (0.51–0.81)	87.8	0.31 (0.09–0.52)
Western Sub-Saharan Africa	248 (189–319)	0.29 (0.22–0.37)	680 (504–872)	0.32 (0.23–0.41)	174.2	0.4 (0.31–0.5)

**Figure 2:**
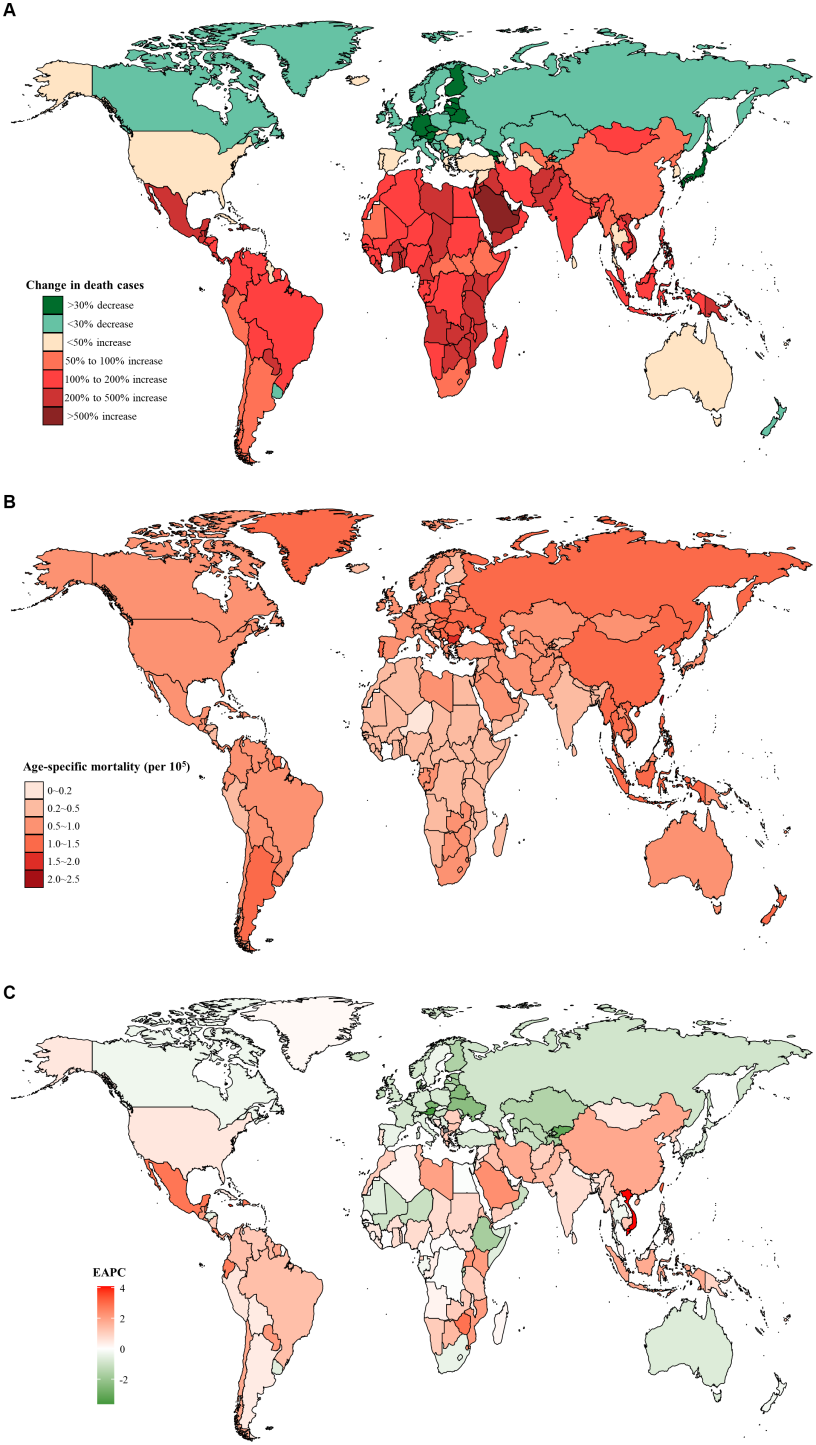
The distribution and trends of early-onset colorectal cancer (CRC) deaths caused by dietary risks in 204 countries and territories, for both sexes combined. **(A)** The percentage change in the number of early-onset CRC deaths caused by dietary risks from 1990 to 2019. **(B)** The age-specific mortality rate of early-onset CRC deaths caused by dietary risks in 2019. **(C)** The estimated annual percentage change of the age-specific mortality rate of early-onset CRC deaths caused by dietary risks from 1990 to 2019.

At the national level, the United Arab Emirates had the largest percentage increase of 819.0% in the number of death cases from 1990 to 2019 (Figure 2A; Supplementary Table S1). In addition, Taiwan (Province of China) had the highest age-specific mortality rate of early-onset CRC due to dietary risks in 2019 (2.13 per 10^5^, 95% UI: 1.45 to 2.97 per 10^5^), followed by Bulgaria and Seychelles (Figure 2B). Moreover, between 1990 and 2019, Vietnam experienced the largest increase in age-specific mortality rate (EAPC = 4.08, 95% CI: 3.84 to 4.33), followed by Lesotho and Jamaica (Figure 2C). However, 70 countries and territories showed a declining age-specific mortality rate, particularly Austria (EAPC = −3.62, 95% CI: −3.83 to −3.41), followed by Kyrgyzstan and Singapore (Figure 2C).

From 1990 to 2019, there was a higher number of death cases and age-specific mortality rate from early-onset CRC attributable to dietary risks observed in males compared to females, and the gap widened with the year (Figure 1B). In 2019, males accounted for 60.1% of dietary risks-associated early-onset CRC death cases (18,095, 95% UI: 13,870 to 21,779), with 39.9% for females (12,001, 95% UI: 9,201 to 14,500) (Table 1). Despite the rising number of death cases for both sexes over the last 30 years, the age-specific mortality rate in females tended to trend downward (EAPC = −0.25, 95% CI: −0.40 to −0.11) (Figure 1B; Table 1).

### 3.2 The distribution and trend of dietary risks-related early-onset colorectal cancer DALYs burden

In 2019, early-onset CRC attributable to dietary risks caused 1,465,755 (95% UI: 1,126,489 to 1,761,661) DALYs worldwide, with an age-specific DALYs rate of 37.3 (95% UI: 28.6 to 44.8) per 10^5^ population (Table 2). Globally, from 1990 to 2019, the overall number of DALYs increased by 67.3% and the age-specific DALYs rate increased by an average of 0.39 (95% CI: 0.30 to 0.47) annually (Table 2). Moreover, all SDI quintiles had seen an increase in the number of DALYs between 1990 and 2019, with the largest increase of 150.2% observed in low SDI areas (Table 2). Furthermore, high SDI areas displayed the least percentage increase of 5.6% in the number of DALYs and a decreasing trend in the age-specific DALYs rate (EAPC = −0.15, 95% CI: −0.24 to −0.06) (Table 2, Figure 1C).

**Table 2:** Early-onset colorectal cancer-related DALYs burden attributable to dietary risks in 1990 and 2019, and its temporal trends from 1990 to 2019.

Characteristics	1990		2019		1990 ~ 2019	
	DALYs No. (95% UI)	ASR per 100,000 No. (95% UI)	DALYs No. (95% UI)	ASR per 100,000 No. (95% UI)	Percent change in absolute number (%)	EAPC No. (95% CI)
Overall	876,027 (694,064–1,027,853)	32.3 (25.6–37.9)	1,465,755 (1,126,489–1,761,661)	37.3 (28.6–44.8)	67.3	0.39 (0.30–0.47)
Sex						
Male	474,777 (374,728–556,686)	34.5 (27.3–40.5)	882,821 (675,572–1,059,758)	44.4 (34.0–53.3)	85.9	0.88 (0.75–1.02)
Female	401,249 (316,868–474,665)	30.0 (23.7–35.5)	582,934 (446,501–701,889)	30.0 (23.0–36.1)	45.3	−0.29 (−0.44−−0.14)
Socio-demographic index						
High	187,687 (142,347–225,590)	43.6 (33.1–52.5)	198,216 (147,141–237,792)	42.2 (31.3–50.6)	5.6	−0.15 (−0.24−−0.06)
High -middle	258,005 (200,138–305,618)	42.6 (33.1–50.5)	372,487 (27,3,815–458,885)	51.1 (37.6–63.0)	44.4	0.23 (0.10–0.36)
Middle	278,765 (227,365–325,927)	30.9 (25.2–36.1)	545,173 (425,520–659,707)	43.3 (33.8–52.3)	95.6	1.19 (1.08–1.30)
Low-middle	110,475 (88,379–131,280)	20.4 (16.4–24.3)	247,314 (187,935–298,529)	26.5 (20.1–32.0)	123.9	0.88 (0.77–0.99)
Low	40,661 (31,448–50,665)	17.5 (13.5–21.8)	101,722 (78,473–122,107)	18.8 (14.5–22.6)	150.2	0.24 (0.19–0.29)
GBD regions						
High-income Asia Pacific	44,572 (33,606–54,589)	48.0 (36.2–58.8)	35,531 (27,241–42,858)	43.8 (33.6–52.8)	−20.3	−0.41 (−0.52−−0.31)
Central Asia	14,884 (11,383–17,595)	44.6 (34.1–52.7)	17,254 (12,728–21,069)	35.3 (26.1–43.2)	15.9	−1.55 (−1.79−−1.31)
East Asia	280,270 (217,801–338,770)	40.6 (31.6–49.1)	490,135 (357,798–619,546)	65.7 (47.9–83.0)	74.9	1.75 (1.62–1.87)
South Asia	76,013 (59,398–92,301)	14.4 (11.2–17.4)	181,198 (134,262–227,015)	18.6 (13.8–23.3)	138.4	0.82 (0.72–0.92)
Southeast Asia	84,939 (68,506–101,388)	35.9 (29.0–42. 9)	198,755 (153,768–244,785)	54.9 (42.5–67.6)	134.0	1.3 (1.14–1.46)
Australasia	5,617 (4,326–6,741)	52.1 (40.1–62.5)	6,155 (4,578–7,470)	45.5 (33.9–55.2)	9.6	−0.48 (−0.57−−0.39)
Caribbean	5,127 (3,863–6,138)	28.1 (21.2–33.7)	8,868 (6,240–11,562)	37.1 (26.1–48.4)	73.0	0.89 (0.81–0.96)
Central Europe	32,549 (23,557–40,057)	53.4 (38.6–65.7)	28,289 (19,568–35,942)	53.7 (37.1–68.2)	−13.1	−0.37 (−0.51−−0.24)
Eastern Europe	62,368 (46,575–75,198)	56.5 (42.2–68.2)	53,423 (37,849–68,099)	54.5 (38.6–69.4)	−14.3	−1.32 (−1.77−−0.87)
Western Europe	84,625 (63,583–101,817)	43.8 (32.9–52.6)	70,861 (53,304–85,002)	37.2 (28.0–44.6)	−16.3	−0.69 (−0.87−−0.51)
Andean Latin America	3,543 (2,826–4,280)	19.0 (15.2–23.0)	8,629 (6,008–11,637)	26.1 (18.1–35.1)	143.6	1.19 (1.00–1.39)
Central Latin America	13,865 (10,700–16,464)	17.0 (13.1–20.2)	38,856 (27,634–49,805)	29.5 (21.0–37.8)	180.2	1.94 (1.88–2.00)
Southern Latin America	11,426 (8,947–13,347)	46.7 (36.5–54.5)	19,219 (14,811–23,108)	56.5 (43.5–67.9)	68.2	0.68 (0.61–0.76)
Tropical Latin America	20,765 (15975–24,660)	26.4 (20.3–31.4)	46,386 (34,852–56,688)	38.9 (29.2–47.6)	123.4	1.30 (1.10–1.50)
North Africa and Middle East	35,960 (26408–46,250)	22.1 (16.3–28.5)	88,579 (62,374–111,249)	26.6 (18.7–33.3)	146.3	0.53 (0.26–0.80)
High-income North America	59,603 (44,523–71,793)	40.1 (30.0–48.3)	73,679 (54,380–89,937)	44.2 (32.6–53.9)	23.6	0.45 (0.32–0.58)
Oceania	774 (559–999)	24.4 (17.6–31.6)	2,032 (1,443–2,742)	29.9 (21.2–40.3)	162.5	0.69 (0.67–0.72)
Central Sub-Saharan Africa	4,269 (3,124–5,671)	17.5 (12.8–23.2)	11,276 (7,573–15,379)	18.1 (12.2–24.7)	164.1	0.05 (−0.12–0.22)
Eastern Sub-Saharan Africa	15,548 (11,635–19,733)	18.7 (14.0–23.8)	40,062 (29,370–51,096)	20.1 (14.8–25.7)	157.7	0.16 (0–0.31)
Southern Sub-Saharan Africa	7,371 (5,973–8,836)	28.3 (22.9–33.9)	13,648 (10,514–16,939)	32.3 (24.9–40.0)	85.2	0.26 (0.01–0.50)
Western Sub-Saharan Africa	11,940 (9,122–15,329)	14.0 (10.7–18.0)	32,922 (24,472–42,277)	15.3 (11.4–19.7)	175.7	0.41 (0.32–0.51)

For GBD regions, from 1990 to 2019, the majority of GBD regions (17/21, 81.0%) showed an increase in the number of DALYs, with the largest increase of 180.2% in Central Latin America (Table 2). The region with the highest age-specific DALYs rate for early-onset CRC caused by dietary risks changed from Eastern Europe (56.5 per 10^5^, 95% UI: 42.2 to 68.2 per 10^5^) in 1990 into East Asia (65.7 per 10^5^, 95% UI: 47.9 to 83.0 per 10^5^) in 2019, while Central Latin America experienced the most significant increase of 1.94 (95% CI: 1.88 to 2.00) per year on average in age-specific DALYs rate between 1990 and 2019 (Table 2). Furthermore, High-income Asia Pacific reported the greatest decrease of 20.3% in the number of DALYs, while Central Asia displayed the most significant decreasing trend in the age-specific DALYs rate (EAPC = −1.55, 95% CI: −1.79 to −1.31) (Table 2).

**Figure 3:**
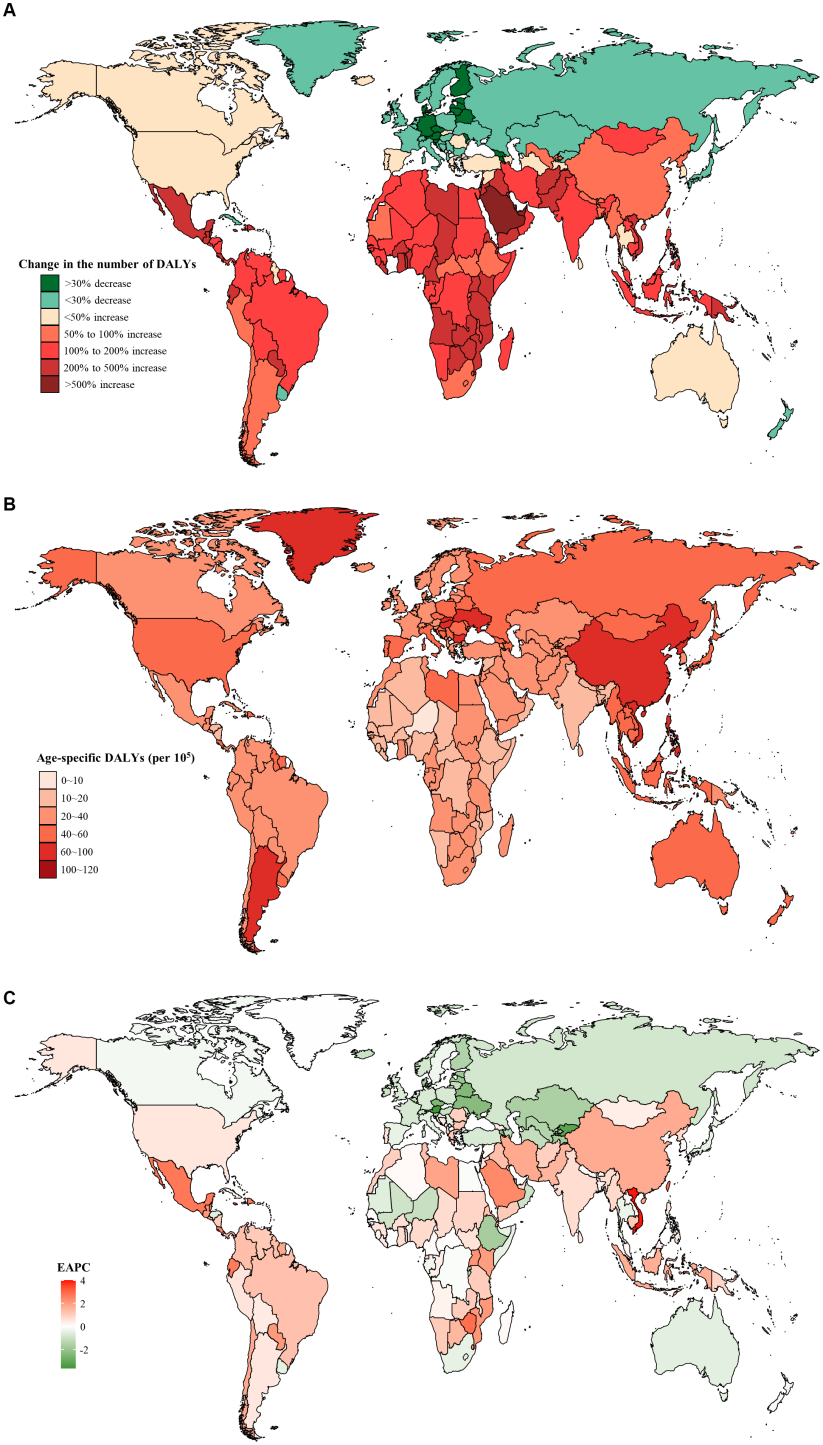
The distribution and trends of early-onset colorectal cancer (CRC)-related disability-adjusted life years (DALYs) attributable to dietary factors for both sexes combined in 204 countries and territories. **(A)** The percentage change in the number of early-onset CRC-related DALYs attributable to dietary risks from 1990 to 2019. **(B)** The age-specific rate of early onset CRC-related DALYs attributable to dietary risks in 2019. **(C)** The estimated annual percentage change of the age-specific rate of early onset CRC-related DALYs attributable to dietary risks from 1990 to 2019.

Moreover, from 1990 to 2019, Austria saw the most significant decrease in both the number of DALYs (decreased by 58.5%) and the age-specific DALYs rate (EAPC = −3.64, 95% CI: −3.86 to −3.42) (Figures 3A,C). Furthermore, the highest age-specific DALYs rate of dietary risks-related early-onset CRC was observed in Ukraine in 1990 (80.9 per 10^5^, 95% UI: 59.8 to 100.2 per 10^5^) and Taiwan (Province of China) in 2019 (104.1 per 10^5^, 95% UI: 71.9 to 144.8 per 10^5^) (Figure 3B).

**Figure 4:**
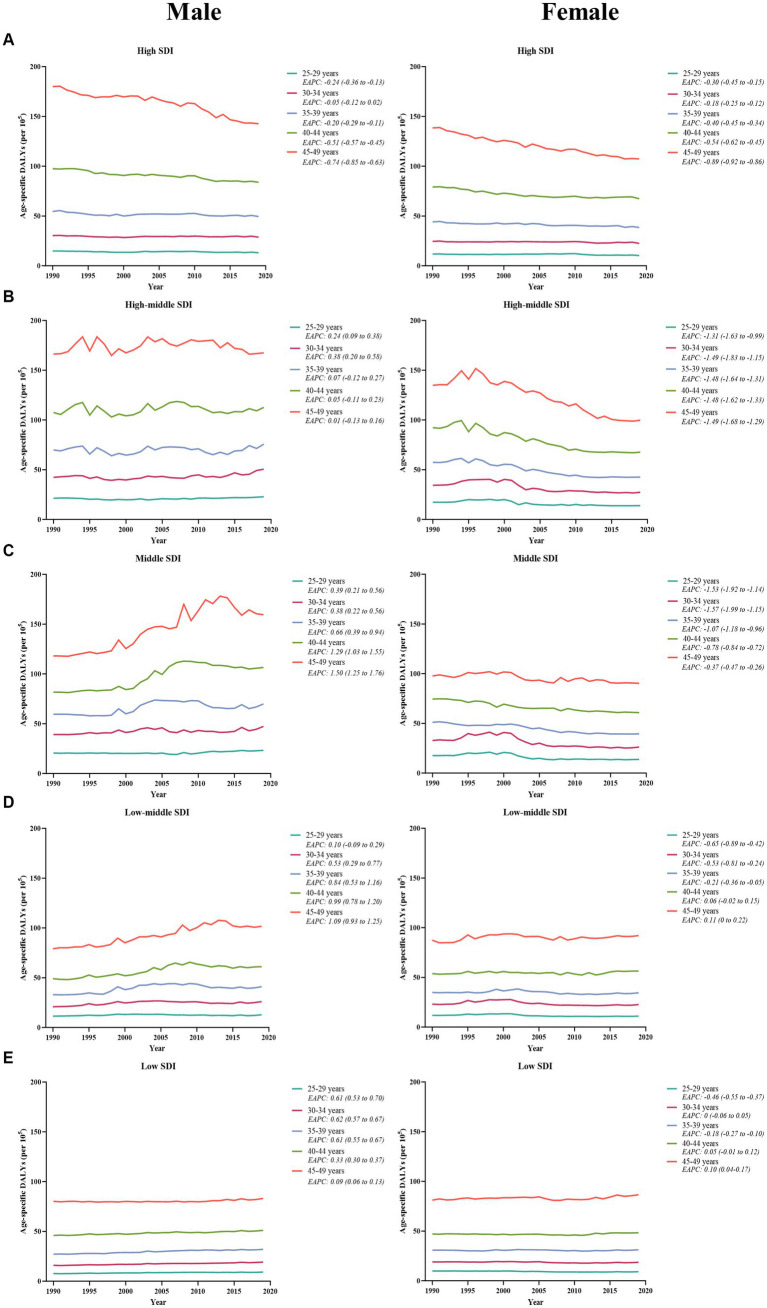
The temporal trend in the age-specific DALYs rate for early-onset colorectal cancer attributable to dietary risks across age groups for males (left panel) and females (right panel) by SDI quintiles, from 1990 to 2019. **(A)** High SDI quintiles. **(B)** High-middle SDI quintiles. **(C)** Middle SDI quintiles. **(D)** Low-middle SDI quintiles. **(E)** Low SDI quintiles.

Between 1990 and 2019, males consistently exhibited a higher number and age-specific rate of DALYs compared to females, and this gap widened over time, particularly after 2000 (Figure 1D). Additionally, the age-specific DALYs rate for early-onset CRC due to dietary risks increased with age for both sexes across all SDI quintiles from 1990 to 2019 (Figure 4). However, the temporal trend in the age-specific DALYs rate across age groups varied by SDI quintiles and sex. In high SDI areas, both male and female adults under 50 years experienced a decrease in age-specific DALYs rate, with the greatest decrease observed in individuals aged 45–49 years old (Figure 4A). Moreover, across all age groups in both high-middle and middle SDI quintiles, dietary risks-attributable early-onset CRC age-specific DALYs rate tended to trend upward among males but displayed a declining trend in females (Figures 4B,C). Furthermore, young male adults had an increasing age-specific DALYs rate across all age groups in low-middle and low SDI quintiles, while the age-specific DALYs rate decreased for young female adults (except the age brackets of 40–44 and 45–49) (Figures 4D,E). Notably, males aged 45–49 years experienced the most pronounced increase in the age-specific DALYs rate burden for early-onset CRC associated with dietary risks in middle and low-middle SDI areas, while the most significant increase was observed in the age group of 30–34 in high-middle and low SDI quintile. Meanwhile, females of 40–45 years of age showed the greatest increase in age-specific DALYs rate burden in low-middle and low SDI areas (Figure 4).

### 3.3 Dietary risk factors for early-onset colorectal cancer burden

**Figure 5:**
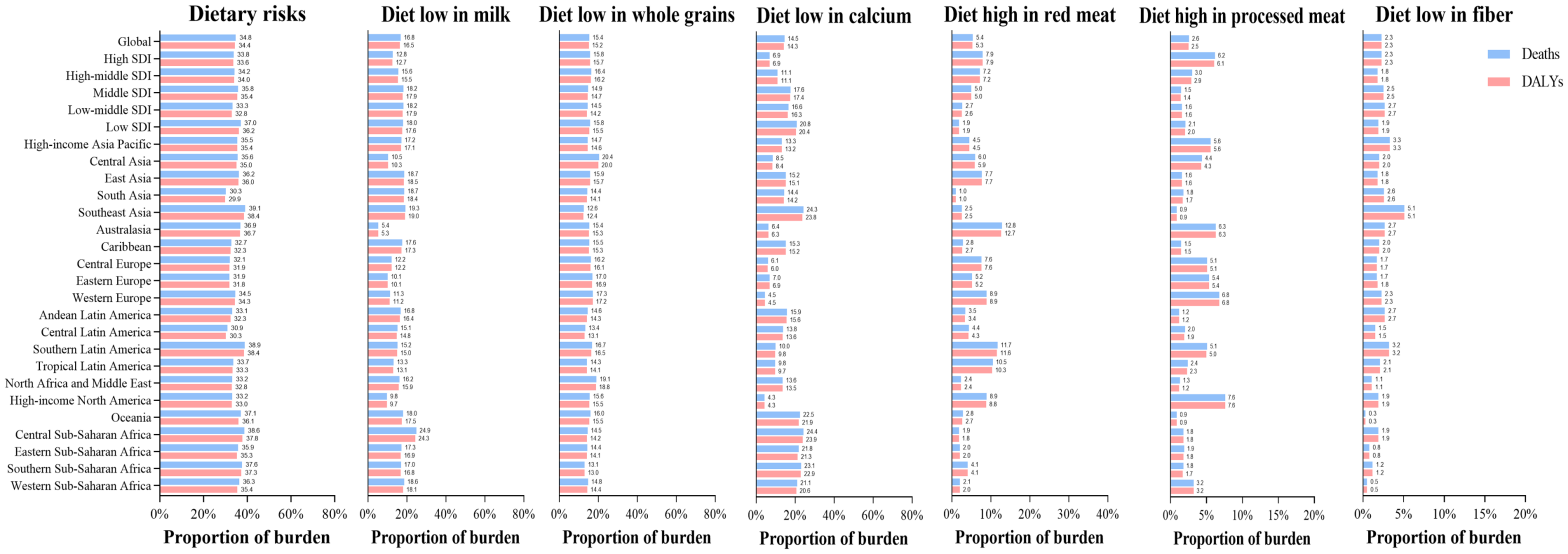
The dietary risk factors for early-onset colorectal cancer burden worldwide, in 5 SDI areas and 21 GBD regions, 2019.

**Figure 6:**
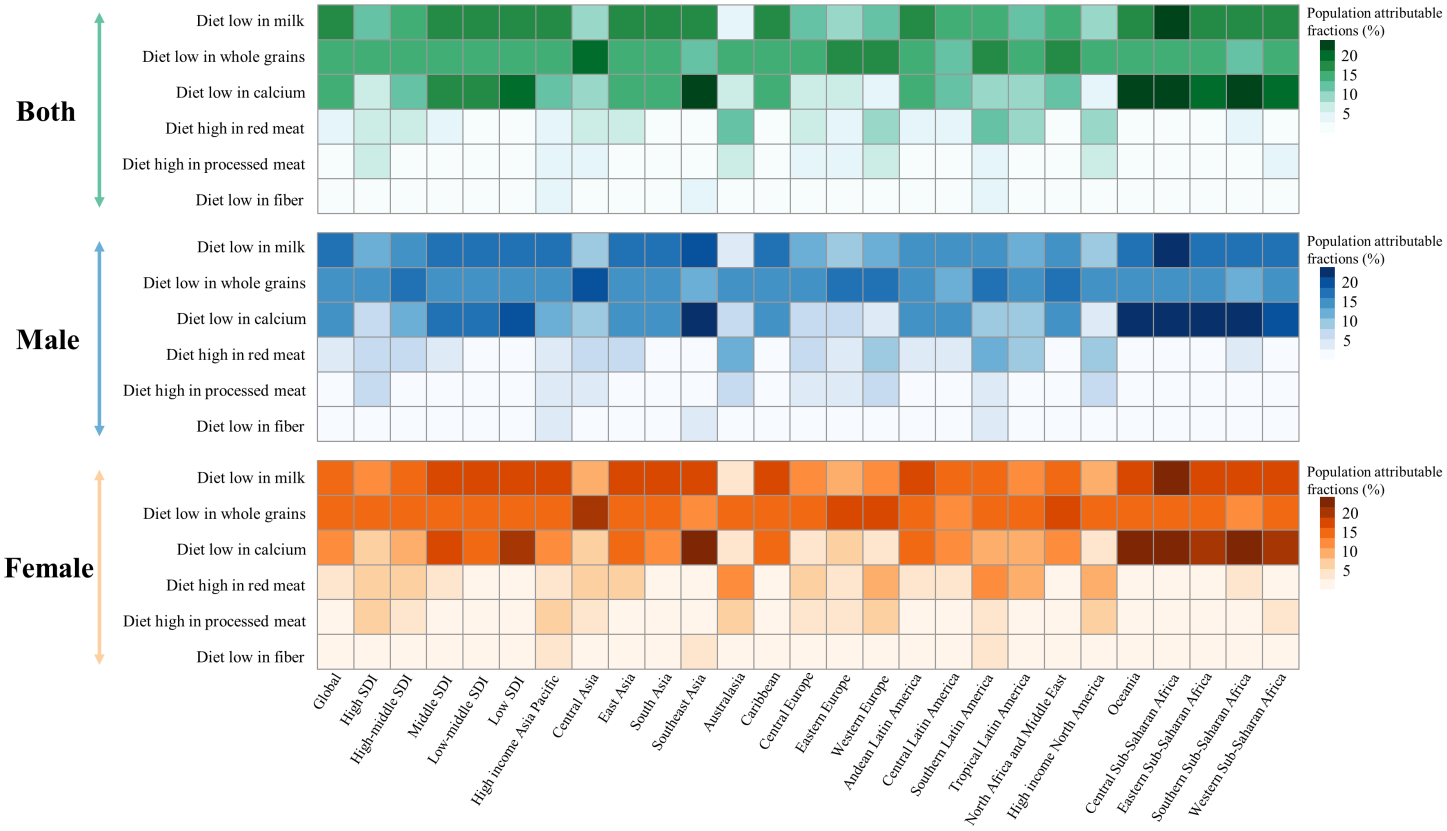
Early-onset colorectal cancer attributable dietary risk factors ranking worldwide, in 5 SDI areas and 21 GBD regions, 2019.

Dietary risks accounted for 34.4% of early-onset CRC DALYs burden worldwide in 2019, mainly consisting of a diet low in milk (16.5%), a diet low in whole grains (15.2%), a diet low in calcium (14.3%), a diet high in red meat (5.3%), a diet high in processed meat (2.5%), and a diet low in fiber (2.3%) (Figure 5). In 2019, the early-onset CRC death burden attributable to dietary factors mirrored the same pattern of the burden of attributable DALYs (Figure 5). Across SDI quintiles, middle SDI areas had the largest number of early-onset CRC DALYs associated with specific dietary factors such as low-milk diet, low-whole grains diet, low-calcium diet, and low-fiber diet. However, the greatest number of DALYs attributable to a diet high in red meat and a diet high in processed meat was observed in high-middle quintiles and high quintiles, respectively (Supplementary Tables S6, S7). In 2019, although a diet low in milk contributed the most to global DALYs for both sexes, a low-whole grains diet was the most important risk factor among most GBD regions (Figures 5, 6). The dietary risk factors were ranked in descending order according to the proportion they contributed to the total number of DALYs for early-onset CRC. Moreover, at the national level, the highest early-onset CRC-related age-specific DALYs rate attributable to a low-milk diet was reported in Seychelles, a low-whole grains diet in Taiwan (Province of China), a low-calcium diet in the Philippines, low-fiber diet in Vietnam, high-red meat diet in Taiwan (Province of China), and high-processed meat diet in Greenland (Supplementary Table S8).

**Figure 7:**
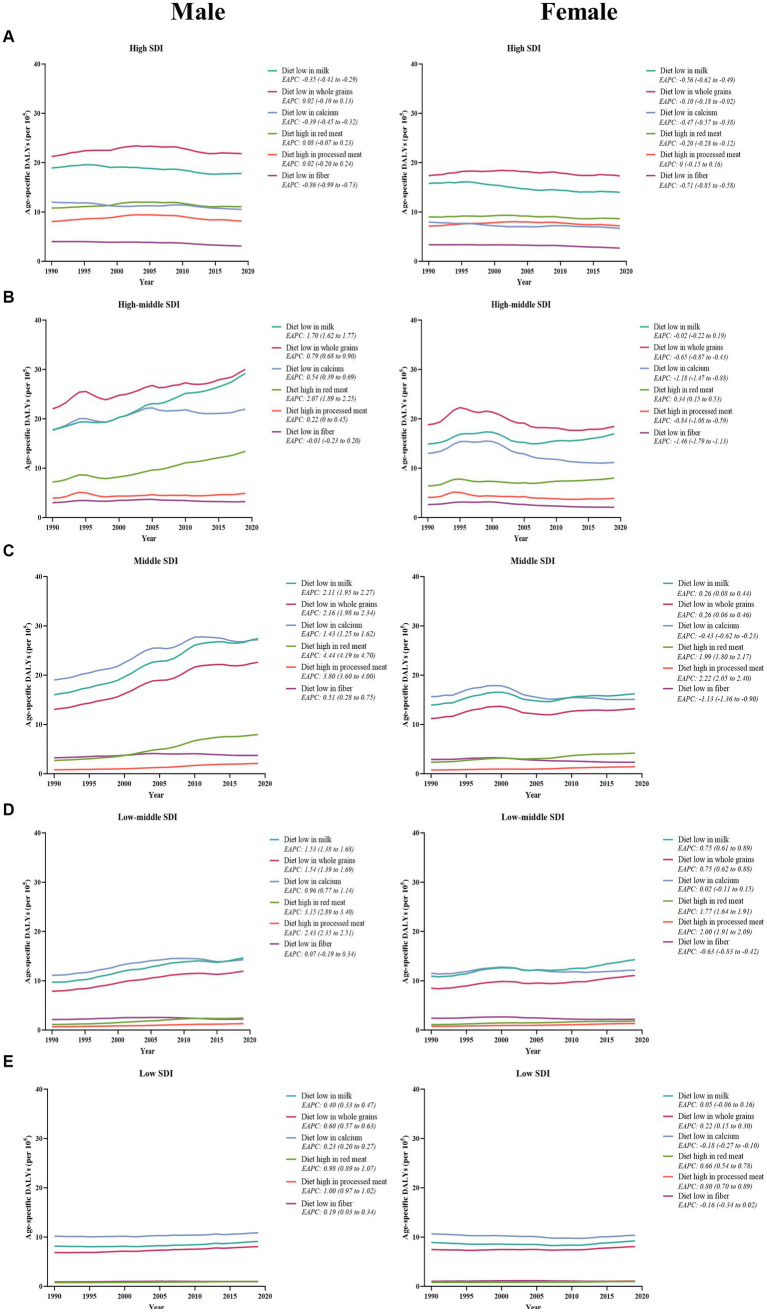
The temporal trend in the age-specific DALYs rate for early-onset colorectal cancer attributable to dietary factors among males (left panel) and females (right panel) between 1990 and 2019, by SDI quintiles. **(A)** High SDI quintiles. **(B)** High-middle SDI quintiles. **(C)** Middle SDI quintiles. **(D)** Low-middle SDI quintiles. **(E)** Low SDI quintiles.

The temporal change in early-onset CRC-related age-specific DALYs rate attributable to dietary risk factors varied by SDI quintiles for both young male adults and young female adults (Figure 7). Between 1990 and 2019, low-milk, low-calcium, and low-fiber diet-related early-onset CRC DALYs decreased in high SDI quintiles for males, while all 6 dietary factors-related DALYs burden were on the decline for females in the same areas (Figure 7A). Additionally, in high-middle SDI quintiles, dietary factors-related DALYs burden (barring low-fiber diet-attributable DALYs) showed an increasing trend among males. In contrast, females had a decreasing dietary factors-related DALYs burden (except high-red meat diet-attributable DALYs) among high-middle SDI areas (Figure 7B). Moreover, the DALYs burden due to all 6 dietary factors and 4 dietary factors (barring low-calcium and low-fiber diet) were on the rise in males and females, respectively, across middle-, low-middle-, and low-SDI areas, with the most significant increase of DALYs attributable to high-red meat diet and high-processed meat diet for both sexes (Figures 7C,D,E). Furthermore, the greatest contributor to dietary factors-related early-onset CRC DALYs burden changed from a low-calcium diet in 1990 to a diet low in milk in 2019 among middle and low-middle SDI regions (Figures 7C,D).

### 3.4 The influential factors for EAPC

**Figure 8:**
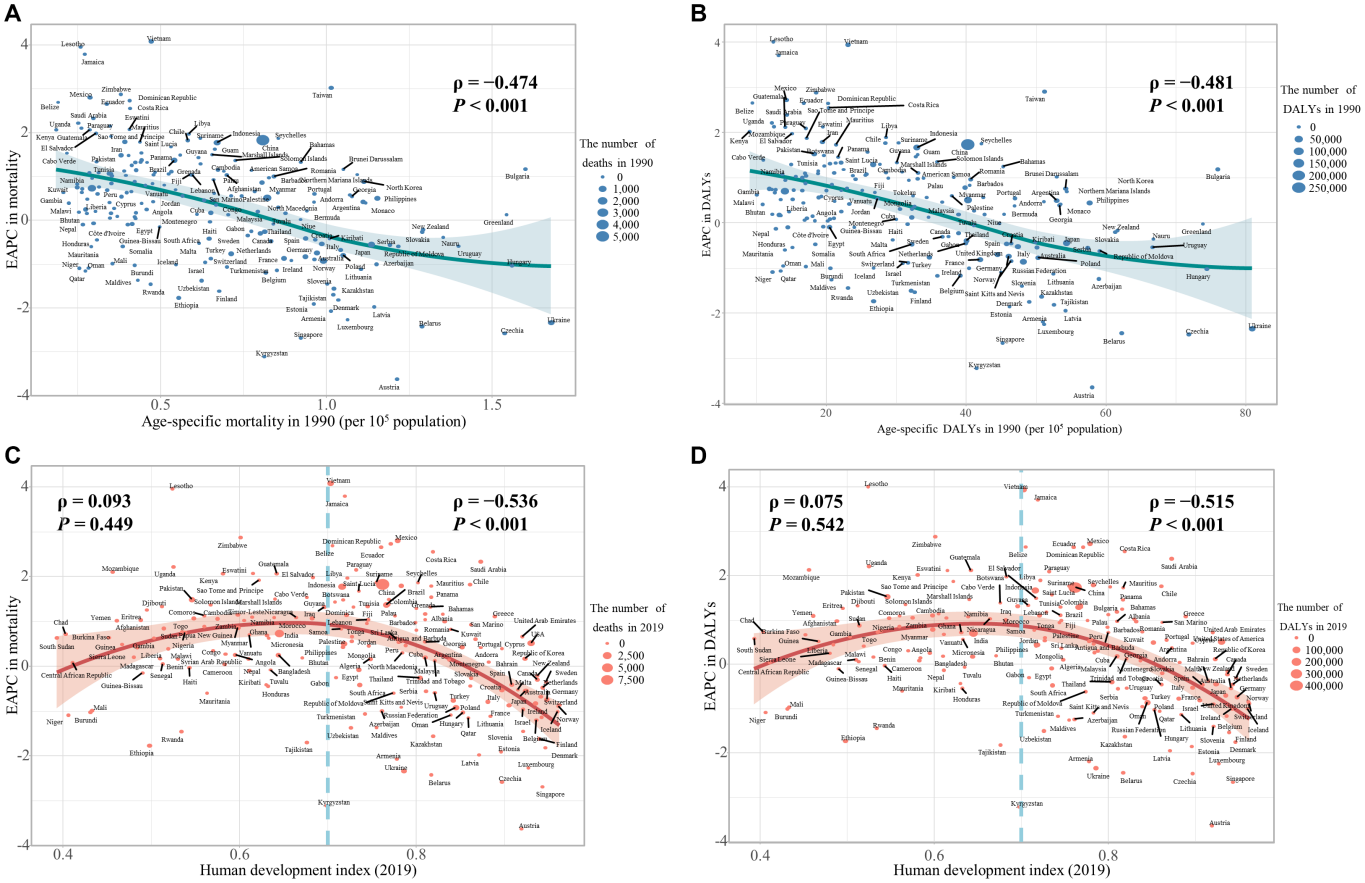
The influential factors for EAPC of age-specific mortality rate and age-specific DALYs rate of colorectal cancer (CRC) are attributable to dietary risks. **(A)** The correlation between EAPC of age-specific mortality rate and age-specific mortality rate in 1990. **(B)** The correlation between EAPC of age-specific DALYs rate and age-specific DALYs rate in 1990. **(C)** The correlation between EAPC of age-specific mortality rate and HDI in 2019. **(D)** The correlation between EAPC of age-specific DALYs rate and HDI in 2019. The size of each circle is proportional to the number of death cases **(A,C)** and DALYs **(B,D)**, respectively. The ρ indices and *p* values were derived from Pearson correlation analysis.

**Figure 9:**
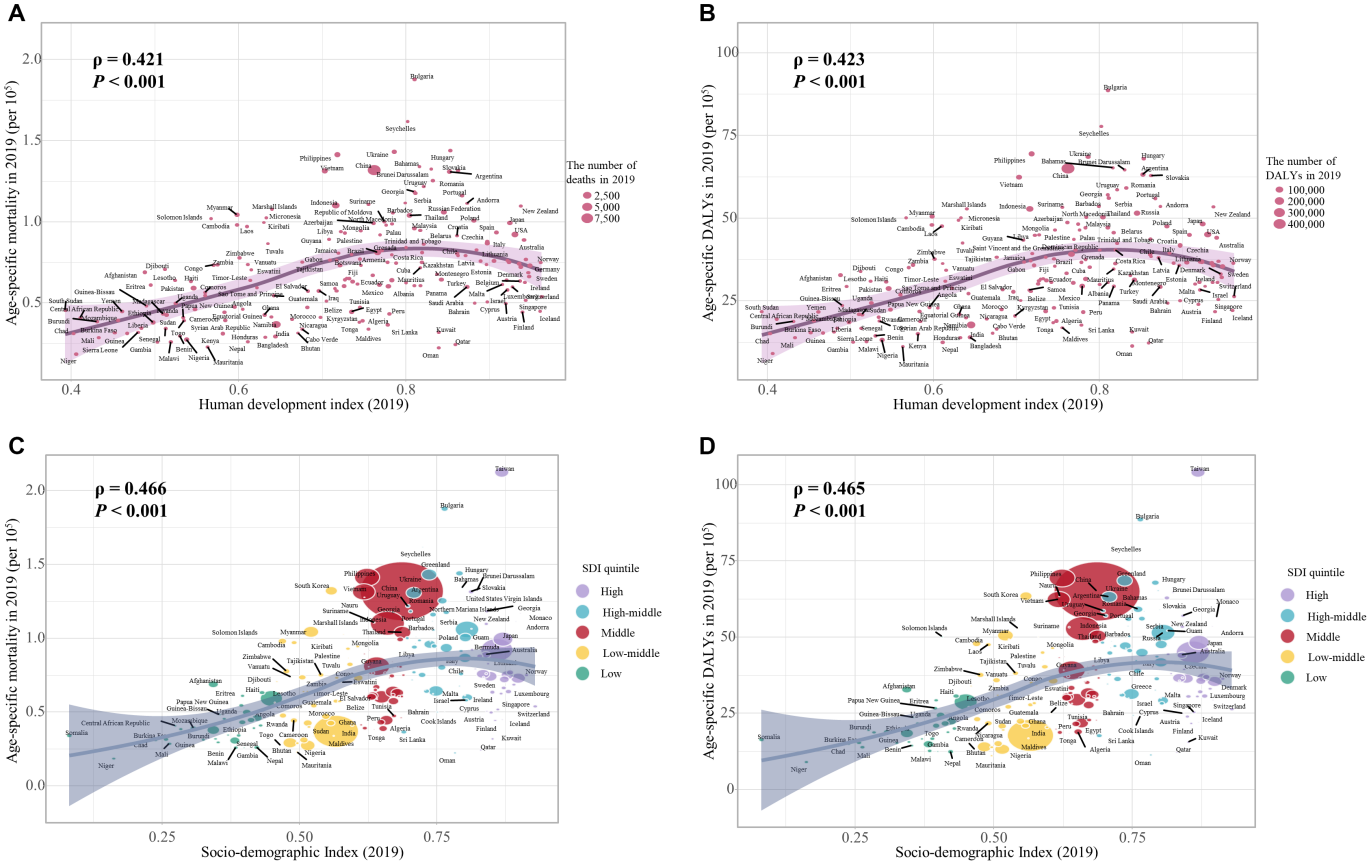
The correlation between the burden of dietary risks-related early-onset colorectal cancer and HDI value and SDI quintile in 2019. **(A)** The correlation between age-specific mortality rate and HDI value; **(B)** The correlation between age-specific DALYs rate and HDI value; **(C)** The correlation between age-specific mortality rate and SDI quintile; **(D)** The correlation between age-specific DALYs rate and SDI quintile. The size of each circle is proportional to the number of death cases **(A, C)** and DALYs **(B, D)**, respectively. The ρ indices and *p* values were derived from Pearson correlation analysis.

Our study has found a significant negative association between the age-specific burden for early-onset CRC due to dietary risks in 1990 and its EAPC between 1990 and 2019. The correlation coefficients (ρ) were − 0.474 (*p* < 0.001) for the death burden (Figure 8A) and − 0.481 (*p* < 0.001) for the DALYs burden (Figure 8B). Additionally, the association between EAPC and HDI scores in 2019 displayed an inverted “U” shape. A positive correlation was observed when the HDI scores were below 0.7, while both EAPC of age-specific mortality rate (ρ = −0.536, *p* < 0.001) and age-specific DALYs rate (ρ = −0.515, *p* < 0.001) were negatively associated with HDI scores when the HDI scores were 0.7 and above (Figures 8C,D). Furthermore, in 2019, the age-specific burden increased with HDI scores (Figure 9).

### 3.5 The summary exposure value and trend for attributable dietary risk factors

Across the globe, all SDI quintiles, and the most of world regions, the age-specific SEV rate of dietary risks related to early-onset CRC was higher in males versus females, and this rate tends to decline in both sexes, although the decrease is slower in males than in females (Table 3). In terms of GBD regions, East Asia had the highest age-specific SEV rate for both males (80.3, 95% UI: 73.4 to 84.6) and females (77.5, 95% UI: 69.0 to 82.6). From 1990 to 2019, males in Eastern Sub-Saharan Africa (ARC = −0.26, 95% UI: −0.43 to −0.01) and females in High-income Asia Pacific (ARC = −0.30, 95% UI: −0.45 to −0.17) experienced the most significant decreasing trends of SEV rate, respectively (Table 3).

**Table 3:** Global and regional age-specific summary exposure value rates of dietary risks attributable to early-onset colorectal cancer in 1990 and 2019, and its temporal trends from 1990 to 2019, by sex.

Characteristics	Male			Female		
	Age-specific SEV rate in 1990 (95% UI)	Age-specific SEV rate in 2019 (95% UI)	ARC (%) (95% UI)	Age-specific SEV rate in 1990 (95% UI)	Age-specific SEV rate in 2019 (95% UI)	ARC (%) (95% UI)
Overall	55.6 (44.0–66.5)	51.1 (38.2–63.5)	−0.08 (−0.14−−0.03)	50.2 (39.4–62.4)	44.3 (33.3–58.4)	−0.12 (−0.17−−0.06)
Socio-demographic index						
High	48.5 (36.5–60.3)	48.4 (32.9–61.8)	0 (−0.12–0.07)	42.4 (32.3–55.3)	37.9 (27.4–51.4)	−0.11 (−0.18−−0.04)
High -middle	63.6 (52.8–71.9)	61.3 (49.8–69.9)	−0.04 (−0.08–0)	55.1 (46.0–64.6)	52.2 (42.5–61.8)	−0.05 (−0.1−−0.02)
Middle	62.0 (51.1–70.9)	53.5 (41.1–64.4)	−0.14 (−0.21−−0.07)	57.4 (46.8–67.1)	48.2 (37.5–59.5)	−0.16 (−0.22−−0.11)
Low-middle	48.5 (34.7–61.9)	47.4 (32.6–61.3)	−0.02 (−0.09–0.02)	43.9 (31.1–59)	40.1 (26.8–56.7)	−0.08 (−0.15−−0.03)
Low	37.8 (23.5–62.0)	36.1 (24.2–59.8)	−0.05 (−0.13–0.07)	38.3 (23.2–63.1)	34.7 (22.1–61.7)	−0.09 (−0.2−−0.01)
GBD regions						
High-income Asia Pacific	69.9 (57.7–76.7)	53.9 (36.8–66.5)	−0.23 (−0.4−−0.11)	59.3 (43.2–70.3)	41.6 (25.8–56.7)	−0.3 (−0.45−−0.17)
Central Asia	66.0 (51.1–76.9)	51.6 (32.1–69.0)	−0.22 (−0.41−−0.08)	43.8 (30.5–60.1)	33.7 (22.6–49.8)	−0.23 (−0.35−−0.13)
East Asia	83.9 (78.1–87.3)	80.3 (73.4–84.6)	−0.04 (−0.09–0)	81.9 (75.4–85.6)	77.5 (69.0–82.6)	−0.05 (−0.12–0)
South Asia	43.6 (28.6–57.8)	47.5 (30.8–60.8)	0.09 (0.02–0.17)	41.1 (27.2–56.4)	38.6 (24.6–54.0)	−0.06 (−0.12−−0.01)
Southeast Asia	54.1 (35.5–67.3)	44.0 (25.8–59.4)	−0.19 (−0.34−−0.08)	47.5 (29.2–63.1)	39.2 (22.6–55.8)	−0.18 (−0.3−−0.08)
Australasia	48.9 (31.2–65.7)	46.2 (28.5–65.8)	−0.05 (−0.25–0.14)	36.1 (27.3–49.9)	34.8 (25.7–49.7)	−0.04 (−0.13–0.08)
Caribbean	29.6 (18.5–45.1)	28.4 (17.0–44.0)	−0.04 (−0.14–0.08)	24.3 (16.7–36.9)	22.7 (15.3–36.3)	−0.06 (−0.13–0.03)
Central Europe	77.8 (68.5–83.2)	75.5 (62.6–82.5)	−0.03 (−0.12–0.03)	56.4 (41.3–68.9)	46.2 (27.4–63.5)	−0.18 (−0.35−−0.06)
Eastern Europe	52.3 (34.6–69.5)	48.8 (31.0–66.0)	−0.07 (−0.23–0.11)	39.9 (28.3–56.5)	36.5 (24.0–53.4)	−0.09 (−0.21–0.05)
Western Europe	45.0 (29.6–59.8)	48.6 (31.2–64.0)	0.08 (−0.02–0.19)	33.8 (25.1–47.4)	34.7 (24.6–48.9)	0.03 (−0.05–0.11)
Andean Latin America	40.9 (22.9–56.2)	40.6 (22.7–57.4)	−0.01 (−0.18–0.18)	32.1 (20.2–48.3)	30.4 (18.9–45.7)	−0.05 (−0.17–0.06)
Central Latin America	48.4 (31.6–62.8)	47.9 (31.1–62.3)	−0.01 (−0.13–0.12)	39.2 (22.8–55.6)	37.7 (21.9–53.9)	−0.04 (−0.14–0.06)
Southern Latin America	53.0 (34.2–68.1)	50.0 (30.4–65.4)	−0.06 (−0.21–0.1)	40.7 (29–56.2)	37.3 (25.7–53.6)	−0.08 (−0.2–0.01)
Tropical Latin America	45.1 (26.4–61.7)	45.1 (26.7–62.1)	0 (−0.21–0.29)	37.8 (24.9–54.1)	37.6 (26.1–52.8)	−0.01 (−0.16–0.16)
North Africa and Middle East	25.1 (16.1–38.9)	24.6 (15.3–38.5)	−0.02 (−0.1–0.07)	21.7 (15.8–30.8)	20.0 (14.5–28.9)	−0.08 (−0.14−−0.02)
High-income North America	36.9 (26.8–50.2)	49.4 (31.5–63.9)	0.34 (0.1–0.61)	37.7 (26.5–53.1)	36.9 (25.4–52.3)	−0.02 (−0.13–0.11)
Oceania	25.5 (19.7–34.9)	25.4 (19.0–37.0)	0 (−0.12–0.17)	25.3 (19.7–34.1)	24.9 (18.5–36.1)	−0.02 (−0.13–0.14)
Central Sub-Saharan Africa	31.3 (19.5–61.4)	31.9 (20.1–63.2)	0.02 (−0.14–0.2)	30.0 (19.2–58.5)	32.3 (20.4–63.9)	0.08 (−0.11–0.27)
Eastern Sub-Saharan Africa	38.9 (22.9–70.1)	28.9 (19.2–56.2)	−0.26 (−0.43−−0.01)	46.3 (23.1–80.4)	34.9 (19.5–73.3)	−0.25 (−0.43−−0.06)
Southern Sub-Saharan Africa	48.8 (26.0–81.8)	41.6 (25.4–74.4)	−0.15 (−0.32–0.04)	43.1 (25.3–78.0)	40.3 (25.0–74.8)	−0.06 (−0.2–0.07)
Western Sub-Saharan Africa	32.9 (19.7–66.6)	31.8 (18.4–65.7)	−0.04 (−0.15–0.08)	32.3 (19.5–65.4)	31.1 (18.2–65.5)	−0.04 (−0.13–0.06)

We further evaluated the age-specific SEV rate and risk-specific trends of each dietary risk factor. Globally, the SEV rate of a diet low in milk has increased for both sexes over the past 30 years (ARC = 0.03, 95% UI: 0.01 to 0.05). All SDI quintiles (barring high-middle SDI) showed a declining trend in the SEV rate, and the low SDI quintile reported the highest SEV rate (Supplementary Table S9). Secondly, the SEV rate of low intake of whole grains showed a slight decrease worldwide for both sexes and only increased in the high SDI quintile, with a larger increase in females compared to males (Supplementary Table S10). Thirdly, between 1990 and 2019, the SEV rate of a diet low in calcium showed a decreasing trend across the globe, all SDI quintiles, and the majority of GBD regions (except Oceania and Central Sub-Saharan Africa), for both sexes (Supplementary Table S11). Fourthly, the SEV rate of a low-fiber diet was slightly higher in females than in males globally and decreased from 1990 to 2019 for both sexes (Supplementary Table S12). Fifthly, the SEV rate of a diet high in red meat tends to trend upward globally and in all SDI quintiles (except the high SDI quintile) (Supplementary Table S13). Sixthly, females had a higher SEV rate of high intake of processed meat than males, and the SEV rate was on the decline in males while remaining stable in females (Supplementary Table S14). It is noteworthy that the highest SEV rate of a diet high in red meat and high in processed meat were both observed in high SDI quintiles (Supplementary Tables S13, S14).

## 4 Discussion

The impact of dietary risks on the burden of non-communicable diseases has been extensively investigated, and it is estimated that one-fifth of global deaths could potentially be prevented by improving diet (28). In the current study, we systematically assessed the global burden of early-onset CRC due to dietary risks at the global, regional, and national levels, as well as the temporal trends over the past three decades. From a global perspective, substantial heterogeneity in dietary risks-related early-onset CRC burdens overall and for each dietary factor was found by world region and nation. However, the age-specific burden for early-onset CRC attributable to dietary risks declined among females during the same period, while it has been on the rise in males. Additionally, the burden of dietary risks-attributable early-onset CRC was significantly greater in males versus females, with the gap widened year by year. Meanwhile, the age-specific SEV rate of dietary risks related to early-onset CRC was higher in males globally compared to females, indicating that dietary risks contribute more to early-onset CRC in males than in females. It was in line with previous reports that diet quality, benchmarked to the Alternative Healthy Eating Index (AHEI), was higher among females than males globally (17, 29). In contrast to higher intakes in males of red and processed meat, bread, and fast food, females tend to avoid high-fat foods and consume higher amounts of milk, fruits and vegetables, and dietary fiber (30–33). Furthermore, there are also differences in CRC survival outcomes between males and females, with females having a significantly lower mortality than males (34). Therefore, gender differences should be taken into account in prevention and screening programs, and prioritizing diet improvement for males is necessary.

In general, the age-specific burden for dietary risks-attributable early-onset CRC increased with SDI quintiles or HDI values. However, the SDI quintile with the highest-burden shifted from a high SDI in 1990 to a high-middle SDI in 2019, while it remained the lowest in the low SDI quintile. One possible explanation is that countries with high SDI are more likely to implement routine and early CRC screening, a well-established intervention for reducing the risk of death from CRC (35, 36). In particular, a common and concerning feature of early-onset CRC is delayed diagnosis (37). Notably, although survival of early-onset CRC has improved in high SDI quintiles, mortality is influenced by both incidence and survival, and a substantial rise in incidence can also offset the improvements in survival to some extent (20). On the other hand, high SDI regions have seen an improvement in dietary quality over time, whereas high-middle and middle SDI countries have undergone a dietary transition to Western diets that aggravate the burden of early-onset CRC (17, 29). In summary, the burden of dietary risks-attributable early-onset in high SDI countries has declined somewhat but continues to be prevalent, thus we should maintain ongoing efforts in the future and can never let down the guard. More importantly, there is an urgent need to promote a balanced diet and initiate early screening to lower the fast-rising burden, especially in high-middle and middle SDI countries. Food is medicine, and adopting a healthy dietary pattern is a highly effective approach to enhance health and reduce healthcare costs for all. The dietary patterns demonstrating the most significant health benefits are predominantly plant-based, comprising non-starchy vegetables, whole fruits, whole grains, legumes, and nuts/seeds, as well as healthy protein sources (higher in legumes, fish, and/or poultry, and lower in processed meats and red meat), and include unsaturated fats (such as mono- and polyunsaturated fats) (38, 39).

What’s more, the prevalence of dietary risk factors and their trends varied substantially by world region. Thus, understanding the trends in early-onset CRC burden due to specific dietary factors provides a more comprehensive view of the epidemiologic transition and offers insights into preventing these risk factors. Increased consumption of calcium and milk may be associated with improved survival among patients with CRC (40–42). Calcium might prevent or lower colonic cytotoxicity by binding with bile and fatty acids, while also potentially limiting the growth and distant metastasis of cancer cells (40). Additionally, dairy product is a primary dietary source of not only calcium and vitamin D but also conjugated linoleic acid, which inhibits cancer cell growth (40). The SEV rates of a diet low in calcium decreased with the increase of the SDI quintile, with low-income settings such as Southeast Asia, Sub-Saharan Africa, and Oceania typically being the most affected. It is worth mentioning that the prevalence of low intake of calcium displayed a declining trend globally for both sexes, which may be a mirror of the achievement of the calcium fortification program started in 1990 (43). However, the overall prevalence of insufficient intake of milk was very high and slightly increased over the past 30 years. Dairy product intake is generally low among people living in Asia (except Central Asia), the Caribbean, Latin America, and Africa. In China, dairy foods contribute only 4.3% of dietary calcium intake due to low dairy consumption (44). Significantly, the price of dairy products in African countries is more than three times that of high-income countries, where dairy consumption is more prevalent (45). These price disparities may influence consumer food preferences. In the middle and low-middle SDI quintile, the DALYs burden of early-onset CRC attributable to a diet low in milk has exceeded that attributable to low intake of calcium by 2019, for both sexes. Altogether, we can draw from the example of calcium fortification and consider milk supplementation to effectively reduce the burden of early-onset CRC, particularly in underdeveloped regions.

The high intake of red and processed meat was most prevalent in regions with high SDI. The number of deaths due to CRC among men in Argentina, who consume almost 300 g of beef daily, is higher than in other countries in Latin America (46, 47). Notably, East Asia experienced the greatest increase in age-specific burden of early-onset CRC attributable to a diet high in red and processed meat. Increasing the daily intake of red and processed meat by 100 g can raise the CRC risk by 12%, due to carcinogenic compounds formed from cooking red and processed meat at high temperatures, which directly contribute to colorectal carcinogenesis (48). To summarize, reducing consumption of processed and red meat, particularly when cooked at high temperatures, maybe a sensible strategy to lower the risk of CRC.

In 2017, insufficient intake of whole grains was the most common risk for all-cause mortality and DALYs among young adults aged 25–50 years worldwide (28). Americans eat less than 30 g of whole grain per day on average while consuming 150–180 g of refined grains (49). Meanwhile, a low-whole grains diet-related burden was on the rise globally, except for High-income Asia Pacific, Central Asia, Australasia, and Europe. Whole grains are rich in anticancer substances such as fiber, antioxidants, phytochemicals, etc. It was reported that each 20 g per day increase in whole grain intake was associated with a 38% reduction in CRC risk-specific mortality (50). In contrast, with a fiber-poor western diet fueling gut inflammation (51), dietary fiber, cereal fiber in particular, can be converted by gut microbiota to short-chain fatty acids that help to drive tumor suppression and improve the prognosis of CRC patients (50). Encouragingly, the SEV rate of a low-fiber diet and corresponding early-onset CRC DALYs burden has been on the decline among most world regions. Notably, some African regions are experiencing a transition from fiber-rich diets to calorie-dense diets which may contribute to the increasing early-onset CRC burden (52). Therefore, dietary improvement campaigns combined with local food traditions might be an effective strategy for promoting increased whole grains and or fiber consumption.

Furthermore, it was proven that an imbalanced diet could lead to obesity and diabetes, which are significant risk factors for early-onset CRC (28, 53, 54). A high intake of processed foods and a high-glycemic load of carbohydrates can create a favorable environment for colon proliferation, which appears to increase the risk of developing CRC (5). East Asia had the greatest increase in excess body weight from 1975 to 2016 and the highest age-specific burden of diet-related early-onset colorectal cancer by 2019 (55). Additionally, insufficient whole-grain or excess refined grain intake and high intake of processed meat were the leading dietary risk factors for type 2 diabetes (T2D) burdens, with the lowest proportional diet-attributable burdens of T2D observed in South Asia and Sub-Saharan Africa (56). Coincidentally, these two regions also had the lowest burden of early-onset CRC attributable to dietary risks, which were on the rise. Many dietary risk factors for early-onset CRC are also common for other chronic diseases, suggesting that modern scientific advances in dietary priorities, such as processing methods, food fortification, and dietary patterns, have benefits beyond the prevention of CRC.

Patients with early-onset cancer are particularly vulnerable to the financial burden associated with cancer care. Individuals with early-onset cancer are often at the beginning of their careers, making it challenging to maintain financial stability and employment. Moreover, they may have limited savings or assets and rely on family support to cover the costs exacerbated by work absences (57). Furthermore, survivors of early-onset CRC also face a higher risk of other long-term health issues, including secondary malignancies and cardiovascular disease (58). Therefore, the substantial and far-reaching impact of early-onset CRC on personal financial hardship, employment, and quality of life should prompt heightened vigilance among policymakers and healthcare providers. Additionally, there was a modest increase in diet quality globally between 1990 and 2018 across all world regions, except in South Asia and Sub-Saharan Africa. High-income countries saw an enhancement in dietary quality by boosting the intake of fruits, vegetables, and whole grains while decreasing the consumption of red or processed meats. Conversely, the consumption of red or processed meats has notably risen over time in Asia, Latin America, and the Caribbean. These results indicate the importance of a dual approach focusing on promoting nutritious foods and minimizing detrimental factors, particularly in Asian, Latin American, and Caribbean nations (29). Furthermore, it is crucial and meaningful to forecast the burden of dietary risk-related early-onset CRC based on the temporal trends in dietary quality among young adults aged 15–49 years (59).

There are some limitations in the current study. Firstly, the accuracy and robustness of the GBD study estimates depended on the quality and quantity of the source data from such as vital registration systems, cancer registration systems, and verbal autopsy systems, which may have been impacted by under-reporting and under-diagnosis in low-income countries with insufficient coverage of health resource (1, 2). Thus, the burden of dietary risks related to early-onset CRC in less well-developed regions may be underestimated due to the absence of high-quality cancer registry data. Secondly, the current study evaluates the burden of early-onset CRC attributable to individual nutrients or foods, rather than dietary patterns. Nonetheless, assessing dietary patterns could provide a more comprehensive understanding of the complexity of dietary intake and its relationship to health outcomes (60). Thirdly, we were unable to further evaluate the distribution and trend for the subsite-specific burden of diet-related early-onset CRC based on anatomical subtypes (proximal colon, distal colon, and rectum). Research has indicated that CRC is a highly heterogeneous condition with variant risk factors and clinical outcomes by anatomical subsites (61). For example, rectal cancer is more common in early-onset CRC and Asian individuals (12). Fourthly, given the emerging data on the clinicopathological differences between early-onset and late-onset CRC (11), it is not evident that the relative risks of dietary risk factors would be the same across these two groups. Previous research has reported that the empirical lifestyle index for hyperinsulinemia (ELIH), a composite measure of diet, BMI, and physical activity, exhibited an even stronger positive association with the risk of early-onset CRC (62). Furthermore, growing evidence suggests that hyperinsulinemia is a plausible mechanism linking diet and CRC (63). These findings indicate that dietary risk factors may play a more prominent role in the development of early-onset compared to late-onset CRC (64).

## 5 Conclusion

In summary, the current study demonstrated that the impact of dietary risks on early-onset CRC is still prevalent globally and has risen from 1990 to 2019. The growing challenge highlights the substantial potential for primary prevention of CRC through dietary modification against the backdrop of modern malnutrition (poor diet quality) and prompts a reevaluation of expanding CRC screening programs to include young adults. Furthermore, understanding the burden of diet-related early-onset CRC in specific geographic and demographic contexts, as well as its temporal changes is crucial for developing targeted prevention strategies and allocating resources to reverse the fast-rising burden.

## Data availability statement

The original contributions presented in the study are included in the article/Supplementary material, further inquiries can be directed to the corresponding authors.

## Author contributions

JS: Writing – review & editing, Writing – original draft, Investigation, Formal analysis, Data curation. YL: Writing – review & editing, Writing – original draft, Visualization, Methodology, Investigation, Formal analysis, Data curation, Conceptualization. XH: Writing – review & editing, Writing – original draft, Supervision, Project administration, Conceptualization.

## Footnotes

^1^http://www.healthdata.org/gbd/about/protocol

^2^http://ghdx.healthdata.org/gbd-results-tool

^3^http://hdr.undp.org/en/data
