# Supplementary material for: The global burden and trends analysis of early-onset colorectal cancer attributable to dietary risk factors in 204 countries and territories, 1990–2019: a secondary analysis for the global burden of disease study 2019
Source: Front Nutr. 2024 May 31;11:1384352. doi: 10.3389/fnut.2024.1384352 (PMC11176521; doi:10.3389/fnut.2024.1384352)
Supplement: Supplementary file 1 [file Table_1.docx]

**Supplementary Table S1. Early-onset colorectal cancer-related burden attributable to dietary risks in 2019, and its temporal change between 1990 and 2019, at national level for both sexes**

| Location | Deaths | | | |  | DALYs | | | |
| --- | --- | --- | --- | --- | --- | --- | --- | --- | --- |
|  | Deaths (in 2019)  No. (95% UI) | Percent change in absolute number (%) | ASR per 10^5^  (in 2019)  No. (95% UI) | EAPC  No. (95% CI) |  | DALYs (in 2019)  No. (95% UI) | Percent change in absolute number (%) | ASR per 10^5^ (in 2019)  No. (95% UI) | EAPC  No. (95% CI) |
| Afghanistan | 125 (67-197) | 276.7 | 0.7 (0.4-1.1) | 1.04 (0.66-1.41) |  | 5,974 (3,211-9,333) | 279.7 | 33 (18-51) | 0.98 (0.67-1.29) |
| Albania | 8 (5-11) | -3.9 | 0.6 (0.4-0.9) | 1.04 (0.82-1.26) |  | 395 (255-572) | -4.3 | 31 (20-44) | 0.97 (0.76-1.18) |
| Algeria | 87 (54-125) | 112.0 | 0.4 (0.2-0.6) | 0.16 (0-0.33) |  | 4,246 (2,617-6,041) | 109.4 | 19 (12-27) | 0.10 (-0.07-0.28) |
| American Samoa | 0 (0-0) | 47.1 | 1.0 (0.7-1.5) | 1.14 (1.04-1.25) |  | 13 (9-20) | 43.5 | 49 (32-71) | 1.01 (0.92-1.11) |
| Andorra | 0 (0-1) | 46.3 | 1.1 (0.7-1.6) | 0.42 (0.29-0.55) |  | 22 (13-32) | 44.4 | 54 (31-77) | 0.38 (0.24-0.51) |
| Angola | 64 (40-93) | 201.5 | 0.5 (0.3-0.7) | 0.26 (0.09-0.43) |  | 3,123 (1,961-4,491) | 201.8 | 23 (14-33) | 0.26 (0.08-0.43) |
| Antigua and Barbuda | 0 (0-0) | 86.1 | 0.6 (0.4-0.8) | 0.81 (0.72-0.90) |  | 14 (10-19) | 81.5 | 30 (20-40) | 0.72 (0.64-0.81) |
| Argentina | 304 (229-370) | 71.8 | 1.3 (1.0-1.6) | 0.43 (0.34-0.51) |  | 14,666 (11,025-17,848) | 74.2 | 63 (48-77) | 0.50 (0.41-0.58) |
| Armenia | 11 (8-14) | -35.9 | 0.8 (0.5-1.0) | -2.06 (-2.39--1.74) |  | 542 (371-705) | -37.7 | 37 (25-48) | -2.19 (-2.49--1.89) |
| Australia | 103 (76-126) | 9.6 | 0.9 (0.7-1.1) | -0.68 (-0.81--0.54) |  | 5,093 (3,749-6,256) | 13.1 | 44 (32-54) | -0.54 (-0.67--0.41) |
| Austria | 20 (14-26) | -58.9 | 0.5 (0.3-0.6) | -3.62 (-3.83--3.41) |  | 968 (698-1,232) | -58.5 | 24 (17-31) | -3.64 (-3.86--3.42) |
| Azerbaijan | 55 (37-74) | 30.7 | 1.0 (0.7-1.3) | -1.00 (-1.33--0.66) |  | 2,710 (1,811-3,604) | 25.1 | 49 (33-65) | -1.24 (-1.62--0.86) |
| Bahamas | 3 (2-4) | 109.1 | 1.3 (0.9-1.9) | 1.33 (1.18-1.48) |  | 134 (91-185) | 107.4 | 65 (44-90) | 1.25 (1.11-1.39) |
| Bahrain | 5 (3-7) | 315.5 | 0.5 (0.3-0.7) | 0.10 (-0.39-0.58) |  | 225 (137-318) | 305.9 | 24 (15-34) | 0.05 (-0.42-0.52) |
| Bangladesh | 253 (154-388) | 83.8 | 0.3 (0.2-0.5) | 0.25 (0.08-0.43) |  | 12,097 (7,321-18,702) | 81.0 | 14 (8-22) | 0.18 (0-0.35) |
| Barbados | 2 (1-2) | 40.6 | 1.1 (0.7-1.6) | 0.97 (0.63-1.32) |  | 75 (50-104) | 37.8 | 54 (36-74) | 0.84 (0.51-1.16) |
| Belarus | 41 (28-58) | -36.5 | 0.9 (0.6-1.3) | -2.42 (-2.86--1.97) |  | 1,990 (1,326-2,782) | -36.7 | 46 (30-64) | -2.44 (-2.88--2.00) |
| Belgium | 32 (23-40) | -21.3 | 0.6 (0.5-0.8) | -1.19 (-1.41--0.98) |  | 1,533 (1,111-1,926) | -21.1 | 31 (22-38) | -1.17 (-1.38--0.96) |
| Belize | 1 (1-1) | 605.9 | 0.5 (0.3-0.7) | 2.70 (2.17-3.22) |  | 56 (39-73) | 603.3 | 25 (17-33) | 2.65 (2.13-3.17) |
| Benin | 17 (11-25) | 211.4 | 0.3 (0.2-0.4) | 0.16 (0.11-0.22) |  | 841 (553-1,214) | 212.1 | 14 (9-21) | 0.16 (0.11-0.22) |
| Bermuda | 0 (0-0) | -14.0 | 1.0 (0.7-1.4) | 0.22 (0.08-0.36) |  | 13 (9-18) | -14.6 | 47 (32-66) | 0.19 (0.05-0.33) |
| Bhutan | 1 (1-2) | 79.2 | 0.3 (0.2-0.5) | 0.19 (0-0.37) |  | 66 (32-108) | 78.4 | 15 (7-25) | 0.20 (0.01-0.38) |
| Bolivia | 35 (22-51) | 146.6 | 0.6 (0.4-0.8) | 0.43 (0.35-0.51) |  | 1,704 (1,058-2,503) | 145.3 | 28 (17-41) | 0.42 (0.34-0.50) |
| Bosnia and Herzegovina | 15 (9-22) | -17.0 | 1.0 (0.6-1.4) | 0.56 (0.39-0.73) |  | 715 (446-1,036) | -19.4 | 47 (29-68) | 0.46 (0.31-0.62) |
| Botswana | 10 (6-16) | 316.7 | 0.8 (0.4-1.2) | 1.49 (1.01-1.96) |  | 492 (270-792) | 315.3 | 37 (20-60) | 1.49 (1.03-1.96) |
| Brazil | 937 (708-1,146) | 126.0 | 0.8 (0.6-1) | 1.37 (1.15-1.59) |  | 45,206 (34,012-55,281) | 121.1 | 39 (29-48) | 1.29 (1.09-1.50) |
| Brunei Darussalam | 4 (2-5) | 129.4 | 1.3 (0.9-1.8) | 1.17 (0.75-1.59) |  | 172 (119-229) | 122.4 | 65 (45-86) | 1.02 (0.59-1.45) |
| Bulgaria | 57 (38-80) | -14.5 | 1.9 (1.3-2.6) | 1.17 (0.85-1.49) |  | 2,686 (1,802-3,748) | -14.9 | 89 (59-124) | 1.19 (0.86-1.52) |
| Burkina Faso | 33 (22-46) | 222.4 | 0.3 (0.2-0.4) | 0.64 (0.40-0.88) |  | 1,577 (1,062-2,226) | 224.9 | 15 (10-22) | 0.67 (0.44-0.90) |
| Burundi | 21 (14-32) | 107.1 | 0.4 (0.2-0.6) | -1.02 (-1.27--0.77) |  | 1,029 (660-1,544) | 106.4 | 19 (12-28) | -1.01 (-1.26--0.75) |
| Cabo Verde | 1 (1-2) | 225.8 | 0.3 (0.2-0.5) | 1.54 (1.33-1.75) |  | 51 (33-76) | 214.6 | 17 (11-25) | 1.39 (1.19-1.59) |
| Cambodia | 86 (61-119) | 178.8 | 1.0 (0.7-1.4) | 1.15 (1.09-1.21) |  | 4,216 (2,967-5,864) | 177.3 | 48 (34-67) | 1.11 (1.04-1.17) |
| Cameroon | 64 (40-98) | 233.1 | 0.4 (0.3-0.7) | 0.05 (-0.01-0.10) |  | 3,128 (1,948-4,747) | 236.7 | 22 (14-33) | 0.08 (0.03-0.14) |
| Canada | 117 (84-149) | -1.0 | 0.7 (0.5-0.9) | -0.29 (-0.42--0.16) |  | 5,775 (4,148-7,327) | 0.7 | 36 (26-45) | -0.23 (-0.34--0.11) |
| Central African Republic | 10 (7-16) | 98.9 | 0.4 (0.3-0.6) | 0.08 (0-0.16) |  | 499 (318-746) | 98.0 | 19 (12-29) | 0.04 (-0.03-0.12) |
| Chad | 21 (15-30) | 225.5 | 0.3 (0.2-0.4) | 0.72 (0.64-0.81) |  | 1,032 (709-1,444) | 225.9 | 15 (10-21) | 0.74 (0.65-0.82) |
| Chile | 74 (57-89) | 78.7 | 0.8 (0.6-1.0) | 1.83 (1.63-2.03) |  | 3,586 (2,761-4,346) | 77.6 | 39 (30-47) | 1.77 (1.57-1.96) |
| China | 9,512 (6,897-12,131) | 76.1 | 1.3 (1.0-1.7) | 1.83 (1.69-1.98) |  | 469,122 (342,528-597,858) | 74.6 | 65 (48-83) | 1.74 (1.61-1.87) |
| Colombia | 153 (100-214) | 103.9 | 0.6 (0.4-0.9) | 1.38 (1.20-1.56) |  | 7,599 (5,025-10,649) | 103.0 | 31 (20-43) | 1.33 (1.16-1.50) |
| Comoros | 2 (1-3) | 164.2 | 0.6 (0.3-0.8) | 1.13 (0.76-1.50) |  | 101 (56-147) | 163.1 | 27 (15-39) | 1.09 (0.72-1.47) |
| Republic of Congo | 20 (12-29) | 166.2 | 0.7 (0.4-1.1) | 0.35 (0.09-0.61) |  | 939 (558-1,398) | 163.1 | 35 (21-52) | 0.31 (0.06-0.56) |
| Cook Islands | 0 (0-0) | -5.1 | 0.5 (0.3-0.7) | 0.80 (0.46-1.15) |  | 2 (1-3) | -6.0 | 24 (13-35) | 0.72 (0.38-1.05) |
| Costa Rica | 20 (13-29) | 222 | 0.8 (0.5-1.1) | 2.56 (2.39-2.73) |  | 1,009 (655-1,422) | 222.2 | 40 (26-57) | 2.55 (2.37-2.73) |
| Côte d'Ivoire | 56 (36-80) | 151.6 | 0.4 (0.3-0.6) | -0.1 (-0.25-0.06) |  | 2,736 (1744-3,912) | 151.5 | 21 (13-30) | -0.08 (-0.23-0.07) |
| Croatia | 17 (10-24) | -27.7 | 0.9 (0.6-1.3) | -0.44 (-0.71--0.17) |  | 814 (496-1,165) | -28.2 | 44 (27-63) | -0.46 (-0.70--0.21) |
| Cuba | 40 (25-55) | 2.4 | 0.7 (0.5-1.0) | 0.47 (0.30-0.63) |  | 1,869 (1,203-2,594) | -0.4 | 35 (23-49) | 0.31 (0.14-0.47) |
| Cyprus | 4 (2-5) | 96.4 | 0.5 (0.4-0.7) | 0.39 (0.17-0.62) |  | 173 (119-222) | 102.2 | 25 (17-32) | 0.48 (0.26-0.7) |
| Czech Republic | 42 (28-59) | -47.3 | 0.9 (0.6-1.2) | -2.57 (-2.84--2.30) |  | 1,998 (1,336-2,794) | -46.7 | 42 (28-58) | -2.46 (-2.71--2.22) |
| North Korea | 184 (113-288) | 55.0 | 1.3 (0.8-2.1) | 0.89 (0.78-0.99) |  | 8,844 (5,427-13,866) | 52.9 | 64 (39-100) | 0.76 (0.68-0.84) |
| Democratic Republic of the Congo | 128 (77-197) | 157.6 | 0.3 (0.2-0.5) | -0.06 (-0.29-0.17) |  | 6,205 (3,783-9,473) | 155.9 | 15 (9-23) | -0.1 (-0.33-0.14) |
| Denmark | 18 (13-22) | -36.4 | 0.7 (0.5-0.9) | -1.81 (-2.06--1.57) |  | 849 (611-1,061) | -35.2 | 33 (24-41) | -1.75 (-2.00--1.50) |
| Djibouti | 5 (3-7) | 342.7 | 0.7 (0.4-1.1) | 1.33 (1.20-1.46) |  | 218 (123-351) | 338.0 | 34 (19-55) | 1.33 (1.20-1.46) |
| Dominica | 0 (0-0) | 37.1 | 0.7 (0.4-0.9) | 1.32 (1.06-1.59) |  | 11 (7-15) | 35.3 | 32 (19-44) | 1.18 (0.92-1.45) |
| Dominican Republic | 48 (30-68) | 221.5 | 0.8 (0.5-1.2) | 2.74 (2.57-2.91) |  | 2,327 (1,478-3,344) | 216.2 | 40 (26-58) | 2.65 (2.48-2.81) |
| Ecuador | 62 (42-86) | 268.9 | 0.7 (0.5-0.9) | 2.66 (2.27-3.05) |  | 3,071 (2,129-4,253) | 269.9 | 33 (23-46) | 2.65 (2.26-3.04) |
| Egypt | 230 (140-339) | 106.7 | 0.4 (0.3-0.7) | -0.07 (-0.41-0.26) |  | 11,232 (6,857-16,504) | 104.8 | 22 (13-32) | -0.09 (-0.46-0.28) |
| El Salvador | 19 (12-27) | 147.0 | 0.6 (0.4-0.8) | 1.99 (1.63-2.34) |  | 930 (592-1,356) | 146.8 | 28 (18-41) | 1.98 (1.62-2.33) |
| Equatorial Guinea | 3 (2-6) | 388.0 | 0.5 (0.2-0.8) | 1.03 (0.85-1.21) |  | 171 (88-290) | 405.3 | 23 (12-39) | 1.15 (0.97-1.32) |
| Eritrea | 21 (14-31) | 275.6 | 0.6 (0.4-0.9) | 1.26 (1.02-1.49) |  | 1,014 (648-1,505) | 277.2 | 29 (19-44) | 1.26 (1.03-1.50) |
| Estonia | 4 (3-6) | -41.2 | 0.7 (0.4-1.1) | -1.9 (-2.26--1.54) |  | 208 (124-303) | -40.7 | 36 (21-52) | -1.85 (-2.20--1.50) |
| Eswatini | 4 (2-7) | 196.0 | 0.7 (0.4-1.1) | 2.08 (1.52-2.64) |  | 208 (113-333) | 199.2 | 34 (19-55) | 2.12 (1.57-2.68) |
| Ethiopia | 200 (135-287) | 63.0 | 0.4 (0.3-0.5) | -1.77 (-2.13--1.4) |  | 9,719 (6,613-13,929) | 64.4 | 19 (13-27) | -1.73 (-2.1--1.37) |
| Fiji | 3 (2-5) | 46.5 | 0.7 (0.5-1.0) | 1.05 (0.76-1.34) |  | 166 (113-232) | 45.2 | 36 (24-50) | 0.99 (0.69-1.29) |
| Finland | 10 (7-13) | -40.6 | 0.4 (0.3-0.6) | -1.61 (-1.8--1.43) |  | 508 (354-652) | -39.7 | 22 (15-28) | -1.54 (-1.71--1.36) |
| France | 216 (157-263) | -15.1 | 0.8 (0.6-0.9) | -0.87 (-1.05--0.68) |  | 10,420 (7,647-12,693) | -14.9 | 37 (27-45) | -0.81 (-0.98--0.65) |
| Gabon | 7 (4-11) | 105.6 | 0.8 (0.5-1.2) | -0.25 (-0.40--0.09) |  | 339 (204-516) | 103.5 | 37 (22-56) | -0.30 (-0.45--0.15) |
| Gambia | 3 (2-4) | 214.4 | 0.3 (0.2-0.4) | 0.64 (0.42-0.85) |  | 139 (90-209) | 213.3 | 13 (8-19) | 0.62 (0.41-0.83) |
| Georgia | 20 (14-26) | -32.2 | 1.2 (0.8-1.6) | 0.55 (0.17-0.92) |  | 944 (654-1,242) | -34.0 | 57 (40-75) | 0.39 (0.02-0.77) |
| Germany | 262 (191-330) | -32.2 | 0.7 (0.5-0.9) | -0.74 (-1.47--0.01) |  | 12,733 (9,195-16,060) | -30.7 | 35 (26-45) | -0.74 (-1.46--0.01) |
| Ghana | 71 (46-101) | 206.4 | 0.4 (0.3-0.6) | 0.69 (0.63-0.76) |  | 3,486 (2,234-4,970) | 207.6 | 21 (13-30) | 0.72 (0.66-0.78) |
| Greece | 34 (25-41) | 26.4 | 0.8 (0.6-0.9) | 1.11 (1.04-1.19) |  | 1,628 (1,210-1,980) | 25.3 | 36 (27-44) | 1.08 (1.02-1.15) |
| Greenland | 0 (0-1) | -23.8 | 1.5 (1.0-2.1) | 0.13 (-0.44-0.71) |  | 19 (12-27) | -24.7 | 70 (47-100) | -0.02 (-0.57-0.53) |
| Grenada | 0 (0-1) | 88.3 | 0.8 (0.6-1.1) | 1.29 (1.16-1.43) |  | 22 (14-29) | 81.4 | 40 (27-54) | 1.13 (0.99-1.27) |
| Guam | 1 (1-1) | 68.6 | 1.1 (0.7-1.4) | 1.63 (1.43-1.83) |  | 42 (28-57) | 65.7 | 52 (35-70) | 1.51 (1.34-1.69) |
| Guatemala | 52 (37-70) | 425.1 | 0.5 (0.4-0.7) | 2.07 (1.73-2.41) |  | 2,605 (1,847-3,515) | 431.9 | 27 (19-37) | 2.12 (1.78-2.46) |
| Guinea | 18 (12-25) | 145.7 | 0.3 (0.2-0.4) | 0.49 (0.44-0.54) |  | 875 (583-1221) | 147.9 | 15 (10-21) | 0.52 (0.48-0.56) |
| Guinea-Bissau | 5 (3-6) | 108.2 | 0.5 (0.3-0.7) | -0.14 (-0.23--0.06) |  | 224 (151-310) | 110.7 | 24 (16-33) | -0.09 (-0.18--0.01) |
| Guyana | 4 (2-5) | 50.0 | 0.9 (0.6-1.2) | 1.44 (1.11-1.77) |  | 180 (117-251) | 48.4 | 43 (28-60) | 1.33 (1.00-1.67) |
| Haiti | 41 (27-62) | 118.3 | 0.6 (0.4-0.9) | -0.03 (-0.23-0.17) |  | 2,015 (1,310-3,023) | 117.7 | 31 (20-46) | -0.03 (-0.23-0.17) |
| Honduras | 15 (8-24) | 138.7 | 0.3 (0.2-0.5) | -0.44 (-0.60--0.28) |  | 720 (403-1,145) | 132.1 | 14 (8-22) | -0.54 (-0.71--0.37) |
| Hungary | 64 (43-87) | -19.5 | 1.4 (1.0-2.0) | -1.03 (-1.39--0.67) |  | 3,017 (2,026-4,086) | -20.3 | 68 (46-92) | -1.02 (-1.36--0.69) |
| Iceland | 1 (1-1) | 1.7 | 0.5 (0.3-0.6) | -1.01 (-1.21--0.8) |  | 36 (26-46) | 2.1 | 22 (16-28) | -1.00 (-1.20--0.80) |
| India | 2,806 (1,992-3,597) | 128.6 | 0.4 (0.3-0.5) | 0.73 (0.63-0.83) |  | 134,847 (95,576-171,586) | 128.2 | 18 (13-23) | 0.71 (0.60-0.82) |
| Indonesia | 1,574 (1,083-2,130) | 148.5 | 1.1 (0.8-1.5) | 1.78 (1.55-2.01) |  | 75,435 (52,175-101,791) | 142.2 | 53 (37-71) | 1.67 (1.44-1.90) |
| Iran | 303 (229-359) | 192.7 | 0.6 (0.5-0.8) | 1.79 (1.39-2.20) |  | 14,962 (11,298-17,723) | 191.2 | 32 (24-37) | 1.79 (1.39-2.20) |
| Iraq | 133 (89-194) | 301.7 | 0.6 (0.4-0.8) | 1.33 (1.14-1.51) |  | 6,422 (4,336-9,336) | 300.9 | 28 (19-40) | 1.31 (1.15-1.48) |
| Ireland | 15 (10-19) | -3.5 | 0.6 (0.4-0.8) | -1.14 (-1.32--0.95) |  | 713 (502-905) | -1.0 | 31 (22-39) | -1.00 (-1.20--0.81) |
| Israel | 25 (17-32) | 65.3 | 0.6 (0.4-0.7) | -0.95 (-1.21--0.69) |  | 1,241 (846-1,573) | 65.4 | 28 (19-36) | -0.89 (-1.14--0.65) |
| Italy | 220 (168-263) | -22.2 | 0.9 (0.7-1.0) | -0.53 (-0.60--0.46) |  | 10,610 (8,091-12,635) | -22 | 42 (32-50) | -0.53 (-0.61--0.44) |
| Jamaica | 12 (8-17) | 292.6 | 0.8 (0.5-1.1) | 3.80 (3.18-4.41) |  | 602 (378-838) | 293.1 | 39 (25-55) | 3.71 (3.08-4.35) |
| Japan | 515 (387-615) | -30.2 | 1.0 (0.7-1.2) | -0.55 (-0.74--0.36) |  | 24,990 (18,968-29,863) | -29.2 | 48 (37-58) | -0.45 (-0.61--0.29) |
| Jordan | 41 (29-53) | 302.6 | 0.6 (0.5-0.8) | 0.56 (0.46-0.66) |  | 1,969 (1,385-2,551) | 303.4 | 31 (22-40) | 0.54 (0.44-0.64) |
| Kazakhstan | 70 (50-88) | -17.0 | 0.8 (0.5-1.0) | -1.55 (-1.85--1.26) |  | 3,374 (2,414-4,265) | -19.2 | 36 (26-46) | -1.63 (-1.89--1.37) |
| Kenya | 81 (53-113) | 334.4 | 0.3 (0.2-0.4) | 2.07 (1.90-2.24) |  | 3,929 (2,542-5,459) | 329 | 15 (10-21) | 2.02 (1.85-2.19) |
| Kiribati | 1 (0-1) | 62.6 | 1.0 (0.7-1.4) | -0.41 (-0.57--0.25) |  | 30 (21-42) | 63.1 | 49 (34-69) | -0.43 (-0.59--0.27) |
| Kuwait | 9 (6-13) | 255.8 | 0.3 (0.2-0.4) | 0.83 (0.47-1.19) |  | 454 (290-619) | 251.7 | 16 (10-21) | 0.74 (0.39-1.09) |
| Kyrgyzstan | 14 (10-18) | -18.5 | 0.4 (0.3-0.5) | -3.11 (-3.37--2.84) |  | 687 (477-888) | -20.9 | 20 (14-26) | -3.22 (-3.49--2.95) |
| Laos | 38 (23-55) | 132.2 | 1.0 (0.6-1.4) | 0.17 (0.07-0.26) |  | 1,854 (1,122-2,683) | 134.1 | 48 (29-69) | 0.18 (0.08-0.28) |
| Latvia | 6 (4-9) | -55.8 | 0.8 (0.5-1.1) | -1.97 (-2.37--1.57) |  | 312 (198-443) | -55.3 | 38 (24-54) | -1.94 (-2.34--1.55) |
| Lebanon | 21 (13-29) | 129.9 | 0.8 (0.5-1.1) | 1.04 (0.76-1.32) |  | 1,007 (607-1,448) | 134.3 | 38 (23-54) | 1.11 (0.83-1.40) |
| Lesotho | 7 (4-11) | 243.8 | 0.6 (0.4-1.0) | 3.96 (3.63-4.29) |  | 355 (217-537) | 246.8 | 31 (19-47) | 4.01 (3.68-4.34) |
| Liberia | 9 (5-13) | 202.9 | 0.3 (0.2-0.5) | 0.41 (-0.16-1.00) |  | 412 (237-647) | 201.8 | 17 (10-26) | 0.45 (-0.12-1.02) |
| Libya | 39 (24-58) | 241.3 | 0.9 (0.6-1.4) | 1.97 (1.58-2.36) |  | 1,843 (1,146-2,735) | 233.9 | 45 (28-67) | 1.89 (1.52-2.27) |
| Lithuania | 10 (6-14) | -50.4 | 0.8 (0.5-1.2) | -1.11 (-1.5--0.72) |  | 478 (306-669) | -50.2 | 39 (25-55) | -1.13 (-1.52--0.74) |
| Luxembourg | 2 (1-2) | -12.9 | 0.6 (0.4-0.8) | -2.26 (-2.47--2.05) |  | 90 (64-116) | -11.2 | 29 (21-38) | -2.24 (-2.44--2.04) |
| Madagascar | 57 (38-84) | 153.5 | 0.4 (0.3-0.6) | 0.18 (0.03-0.32) |  | 2,771 (1,852-4,051) | 148.0 | 21 (14-31) | 0.1 (-0.05-0.26) |
| Malawi | 23 (15-34) | 146.4 | 0.3 (0.2-0.4) | 0.39 (0.28-0.51) |  | 1,143 (723-1,668) | 145.5 | 13 (8-19) | 0.41 (0.30-0.53) |
| Malaysia | 168 (112-235) | 134.8 | 1.0 (0.6-1.3) | 0.20 (-0.07-0.47) |  | 8,239 (5,553-11,461) | 135.3 | 47 (32-66) | 0.19 (-0.09-0.47) |
| Maldives | 1 (1-1) | 163.6 | 0.3 (0.2-0.5) | -1.36 (-1.79--0.93) |  | 54 (37-72) | 172.5 | 17 (12-23) | -1.26 (-1.70--0.81) |
| Mali | 28 (18-40) | 115.9 | 0.3 (0.2-0.4) | -0.98 (-1.16--0.81) |  | 1,340 (868-1,953) | 117.3 | 14 (9-20) | -0.97 (-1.15--0.79) |
| Malta | 1 (1-2) | 2.8 | 0.6 (0.4-0.8) | -0.52 (-0.69--0.35) |  | 59 (42-76) | 5.2 | 30 (21-39) | -0.38 (-0.52--0.23) |
| Marshall Islands | 0 (0-0) | 147.5 | 1.1 (0.7-1.5) | 1.50 (1.38-1.62) |  | 16 (10-23) | 142.4 | 53 (34-76) | 1.47 (1.36-1.58) |
| Mauritania | 4 (3-7) | 68.1 | 0.2 (0.1-0.4) | -0.60 (-0.72--0.48) |  | 216 (126-345) | 67.9 | 11 (6-18) | -0.6 (-0.72--0.48) |
| Mauritius | 5 (3-6) | 75.6 | 0.7 (0.5-1.0) | 1.79 (1.54-2.04) |  | 225 (144-311) | 73.2 | 35 (22-48) | 1.73 (1.51-1.96) |
| Mexico | 400 (276-528) | 228.2 | 0.6 (0.4-0.8) | 2.81 (2.67-2.94) |  | 19,543 (13,596-25,633) | 221.8 | 29 (21-39) | 2.72 (2.59-2.86) |
| Micronesia (Federated States of) | 1 (0-1) | 36.1 | 1.0 (0.3-1.6) | 0.63 (0.55-0.72) |  | 27 (8-42) | 33.8 | 50 (14-78) | 0.57 (0.49-0.64) |
| Monaco | 0 (0-0) | 8.3 | 1.2 (0.7-1.7) | 0.4 (0.25-0.55) |  | 8 (5-12) | 9.4 | 56 (36-83) | 0.42 (0.27-0.56) |
| Mongolia | 17 (12-24) | 153.3 | 0.9 (0.7-1.3) | 0.46 (0.17-0.74) |  | 840 (590-1,177) | 151.1 | 46 (33-65) | 0.36 (0.06-0.67) |
| Montenegro | 2 (1-3) | 3.2 | 0.7 (0.4-0.9) | 0.14 (-0.16-0.45) |  | 97 (62-131) | 1.1 | 33 (21-44) | 0.08 (-0.19-0.35) |
| Morocco | 91 (57-142) | 111.9 | 0.5 (0.3-0.7) | 1.07 (1.00-1.14) |  | 4,296 (2,708-6,758) | 108.6 | 22 (14-35) | 1.00 (0.93-1.07) |
| Mozambique | 52 (32-78) | 257.7 | 0.4 (0.2-0.6) | 2.10 (1.78-2.43) |  | 2,522 (1,570-3,721) | 260.7 | 19 (12-28) | 2.13 (1.82-2.44) |
| Myanmar | 302 (199-432) | 85.8 | 1.0 (0.7-1.5) | 0.86 (0.63-1.1) |  | 14,627 (9,562-20,905) | 82.2 | 51 (33-72) | 0.79 (0.55-1.02) |
| Namibia | 5 (3-8) | 177.1 | 0.4 (0.3-0.6) | 1.09 (0.74-1.44) |  | 249 (153-385) | 176.7 | 20 (12-31) | 1.07 (0.71-1.43) |
| Nauru | 0 (0-0) | 6.9 | 1.3 (0.7-1.9) | -0.38 (-0.47--0.30) |  | 4 (2-5) | 7.5 | 63 (36-93) | -0.36 (-0.45--0.27) |
| Nepal | 42 (26-64) | 79.6 | 0.3 (0.2-0.4) | -0.09 (-0.44-0.25) |  | 2,021 (1,247-3,047) | 78.1 | 12 (8-19) | -0.13 (-0.47-0.21) |
| Netherlands | 58 (41-73) | -14.5 | 0.8 (0.6-1.0) | -0.47 (-0.6--0.34) |  | 2,776 (1,989-3,506) | -14.2 | 37 (26-47) | -0.46 (-0.58--0.33) |
| New Zealand | 22 (17-27) | -6.4 | 1.1 (0.8-1.3) | -0.21 (-0.35--0.06) |  | 1,062 (824-1,289) | -4.5 | 53 (41-65) | -0.16 (-0.30--0.02) |
| Nicaragua | 14 (10-19) | 160.3 | 0.4 (0.3-0.5) | 1.09 (0.84-1.34) |  | 686 (475-933) | 155.0 | 20 (14-27) | 1.03 (0.76-1.29) |
| Niger | 18 (11-27) | 106.6 | 0.2 (0.1-0.3) | -1.09 (-1.22--0.95) |  | 859 (541-1,303) | 109.0 | 9 (6-14) | -1.09 (-1.22--0.95) |
| Nigeria | 278 (186-405) | 173.6 | 0.3 (0.2-0.4) | 0.75 (0.58-0.93) |  | 13,355 (8,880-19,383) | 174.9 | 13 (9-19) | 0.76 (0.59-0.92) |
| Niue | 0 (0-0) | -21.6 | 1.0 (0.5-1.6) | 0.02 (-0.09-0.13) |  | 0 (0-1) | -22.2 | 47 (24-76) | -0.02 (-0.12-0.09) |
| Macedonia | 11 (7-16) | 28.1 | 1.1 (0.7-1.5) | 0.30 (0.03-0.58) |  | 545 (344-766) | 26.8 | 50 (32-71) | 0.27 (0.01-0.52) |
| Northern Mariana Islands | 0 (0-0) | -19.1 | 1.3 (0.8-1.8) | 1.05 (0.72-1.37) |  | 12 (8-17) | -24.1 | 58 (38-83) | 0.69 (0.40-0.99) |
| Norway | 19 (13-23) | -10.4 | 0.8 (0.5-0.9) | -0.89 (-1.11--0.66) |  | 907 (655-1,138) | -9.8 | 36 (26-46) | -0.84 (-1.08--0.6) |
| Oman | 7 (4-11) | 160.2 | 0.2 (0.1-0.4) | -1.08 (-1.37--0.8) |  | 356 (213-557) | 168.2 | 11 (7-18) | -0.96 (-1.22--0.69) |
| Pakistan | 663 (460-884) | 251.6 | 0.6 (0.4-0.8) | 1.49 (1.20-1.78) |  | 32,167 (22,428-42,845) | 255.1 | 29 (20-38) | 1.53 (1.23-1.82) |
| Palau | 0 (0-0) | 53.2 | 1.0 (0.6-1.4) | 1.12 (1.04-1.21) |  | 4 (3-6) | 48.0 | 46 (29-66) | 0.99 (0.91-1.08) |
| Palestine | 24 (18-29) | 241.4 | 0.9 (0.7-1.2) | 0.67 (0.46-0.87) |  | 1141 (889-1,419) | 240.8 | 45 (35-56) | 0.60 (0.39-0.82) |
| Panama | 13 (8-18) | 134.8 | 0.6 (0.4-0.9) | 1.54 (1.30-1.79) |  | 619 (406-877) | 132.0 | 29 (19-42) | 1.46 (1.23-1.69) |
| Papua New Guinea | 28 (18-40) | 231.6 | 0.5 (0.4-0.8) | 0.89 (0.85-0.93) |  | 1,361 (898-1,946) | 229.4 | 27 (18-38) | 0.85 (0.82-0.89) |
| Paraguay | 24 (15-35) | 269.8 | 0.7 (0.4-0.9) | 2.16 (2.00-2.31) |  | 1,180 (743-1,699) | 266.1 | 32 (20-46) | 2.09 (1.95-2.24) |
| Peru | 77 (48-114) | 89.3 | 0.4 (0.3-0.6) | 0.54 (0.32-0.77) |  | 3,853 (2,387-5,686) | 90.9 | 22 (13-32) | 0.56 (0.35-0.78) |
| Philippines | 827 (628-1,070) | 130.1 | 1.4 (1.1-1.8) | 0.51 (0.31-0.70) |  | 40,597 (30,976-52,305) | 126.1 | 69 (53-89) | 0.44 (0.25-0.64) |
| Poland | 183 (127-240) | -8.0 | 1.0 (0.7-1.3) | -0.79 (-1.03--0.55) |  | 8,664 (5,990-11,336) | -9.1 | 48 (33-62) | -0.77 (-0.97--0.56) |
| Portugal | 54 (40-69) | 13.1 | 1.2 (0.8-1.5) | 0.61 (0.29-0.92) |  | 2,625 (1,893-3,297) | 11.8 | 56 (40-70) | 0.55 (0.23-0.86) |
| Puerto Rico | 15 (9-21) | 11.5 | 0.9 (0.6-1.3) | 0.57 (0.38-0.75) |  | 704 (429-1,018) | 11.2 | 44 (27-64) | 0.56 (0.39-0.74) |
| Qatar | 5 (3-8) | 561.5 | 0.2 (0.1-0.4) | -1.15 (-1.53--0.76) |  | 264 (148-380) | 571.8 | 12 (7-18) | -1.06 (-1.42--0.70) |
| South Korea | 196 (143-251) | 16.1 | 0.8 (0.6-1.0) | 0.17 (-0.28-0.63) |  | 9,587 (,7028-12,297) | 15.0 | 37 (27-47) | 0.12 (-0.32-0.56) |
| Moldova | 18 (13-24) | -31.2 | 1.0 (0.7-1.3) | -0.59 (-0.91--0.28) |  | 886 (602-1160) | -31.8 | 48 (33-63) | -0.62 (-0.93--0.31) |
| Romania | 109 (71-147) | 13.7 | 1.3 (0.8-1.7) | 1.00 (0.73-1.27) |  | 5125 (3354-6906) | 11.2 | 59 (39-80) | 0.97 (0.71-1.22) |
| Russia | 726 (509-947) | -1.3 | 1.1 (0.7-1.4) | -0.92 (-1.5--0.33) |  | 35,293 (24,819-45,913) | -1.3 | 52 (36-67) | -0.86 (-1.40--0.31) |
| Rwanda | 27 (16-40) | 80.0 | 0.4 (0.3-0.6) | -1.45 (-1.89--1.02) |  | 1,299 (799-1,933) | 79.0 | 20 (12-30) | -1.44 (-1.88--1.00) |
| Saint Kitts and Nevis | 0 (0-0) | 40.9 | 0.8 (0.2-1.3) | -0.91 (-1.17--0.65) |  | 12 (4-19) | 31.7 | 37 (11-62) | -1.09 (-1.36--0.82) |
| Saint Lucia | 1 (1-1) | 111.9 | 0.8 (0.6-1.0) | 1.68 (1.45-1.90) |  | 36 (25-47) | 107.8 | 38 (27-50) | 1.57 (1.36-1.78) |
| Saint Vincent and the Grenadines | 1 (0-1) | 76.4 | 0.9 (0.6-1.2) | 1.5 (1.35-1.65) |  | 25 (17-32) | 69.1 | 44 (31-56) | 1.30 (1.13-1.46) |
| Samoa | 1 (0-1) | 65.3 | 0.6 (0.3-0.9) | 0.84 (0.65-1.03) |  | 29 (17-43) | 63.8 | 28 (16-42) | 0.77 (0.58-0.96) |
| San Marino | 0 (0-0) | 51.5 | 0.8 (0.4-1.4) | 0.93 (0.81-1.05) |  | 6 (3-10) | 52.5 | 38 (22-65) | 0.95 (0.84-1.06) |
| Sao Tome and Principe | 1 (0-1) | 264.1 | 0.6 (0.4-0.9) | 1.92 (1.77-2.07) |  | 31 (18-47) | 260.2 | 29 (17-44) | 1.88 (1.73-2.03) |
| Saudi Arabia | 146 (88-216) | 509.8 | 0.6 (0.4-0.9) | 2.33 (2.27-2.4) |  | 7,123 (4,258-10,498) | 513.6 | 29 (17-43) | 2.39 (2.32-2.45) |
| Senegal | 25 (16-36) | 122.6 | 0.3 (0.2-0.5) | 0.06 (-0.19-0.31) |  | 1,227 (757-1,734) | 122.7 | 17 (10-24) | 0.06 (-0.19-0.31) |
| Serbia | 47 (30-68) | -14.0 | 1.2 (0.7-1.7) | -0.56 (-0.91--0.20) |  | 2,247 (1,418-3,239) | -14.7 | 56 (35-81) | -0.55 (-0.87--0.23) |
| Seychelles | 1 (1-1) | 177 | 1.6 (1.1-2.1) | 1.87 (1.61-2.14) |  | 42 (30-55) | 169.7 | 78 (55-101) | 1.75 (1.49-2.02) |
| Sierra Leone | 13 (9-19) | 166.6 | 0.3 (0.2-0.5) | 0.21 (0.01-0.40) |  | 650 (422-928) | 169.6 | 16 (10-22) | 0.25 (0.06-0.44) |
| Singapore | 16 (11-20) | -9.1 | 0.5 (0.4-0.7) | -2.68 (-2.89--2.47) |  | 782 (562-1,004) | -8.9 | 25 (18-33) | -2.66 (-2.86--2.45) |
| Slovakia | 35 (22-50) | 0.5 | 1.3 (0.9-1.9) | -0.26 (-0.46--0.06) |  | 1,656 (1,081-2,368) | 1.1 | 63 (41-90) | -0.2 (-0.39--0.01) |
| Slovenia | 7 (4-10) | -32.9 | 0.8 (0.5-1.1) | -1.45 (-1.78--1.12) |  | 329 (213-480) | -32.3 | 37 (24-54) | -1.40 (-1.70--1.10) |
| Solomon Islands | 3 (2-5) | 204.6 | 1 (0.5-1.5) | 1.37 (1.31-1.43) |  | 165 (86-243) | 203.0 | 50 (26-74) | 1.36 (1.3-1.41) |
| Somalia | 32 (18-61) | 169.7 | 0.3 (0.2-0.6) | -0.59 (-0.88--0.3) |  | 1,550 (884-2,943) | 171.1 | 16 (9-31) | -0.47 (-0.75--0.19) |
| South Africa | 192 (151-238) | 56.4 | 0.6 (0.5-0.8) | -0.41 (-0.64--0.19) |  | 9,511 (7,453-11,851) | 55.0 | 31 (24-39) | -0.47 (-0.73--0.21) |
| South Sudan | 19 (11-32) | 76.8 | 0.5 (0.3-0.8) | 0.64 (0.36-0.91) |  | 918 (531-1,525) | 74.2 | 22 (13-36) | 0.59 (0.33-0.84) |
| Spain | 193 (144-243) | 6.1 | 0.9 (0.7-1.2) | -0.41 (-0.52--0.30) |  | 9,298 (6,892-11,724) | 4.6 | 45 (33-57) | -0.46 (-0.57--0.35) |
| Sri Lanka | 40 (27-57) | 40.6 | 0.4 (0.2-0.5) | 0.6 (0.45-0.76) |  | 1,930 (1,313-2,737) | 38.5 | 18 (12-25) | 0.52 (0.37-0.68) |
| Sudan | 89 (50-141) | 178.0 | 0.4 (0.2-0.7) | 0.85 (0.71-0.98) |  | 4,344 (2,426-6,861) | 180.7 | 21 (12-33) | 0.88 (0.75-1.00) |
| Suriname | 3 (2-4) | 162.2 | 1.1 (0.8-1.5) | 1.87 (1.64-2.1) |  | 154 (111-201) | 158 | 53 (38-70) | 1.77 (1.55-2) |
| Sweden | 30 (21-37) | -7.2 | 0.7 (0.5-0.8) | -0.35 (-0.46--0.23) |  | 1,439 (1,031-1,789) | -6.4 | 32 (23-40) | -0.31 (-0.42--0.19) |
| Switzerland | 22 (16-27) | -8.8 | 0.5 (0.4-0.7) | -0.87 (-1.1--0.63) |  | 1,053 (764-1,312) | -8.4 | 26 (19-33) | -0.88 (-1.11--0.65) |
| Syria | 29 (19-43) | 44.4 | 0.4 (0.3-0.6) | 0.28 (-0.31-0.86) |  | 1,395 (893-2,073) | 37.7 | 19 (12-28) | 0.15 (-0.40-0.70) |
| Taiwan | 248 (170-347) | 117.6 | 2.1 (1.5-3.0) | 3.03 (2.76-3.29) |  | 12,169 (8,400-16,920) | 111.2 | 104 (72-145) | 2.91 (2.65-3.17) |
| Tajikistan | 37 (27-50) | 52.0 | 0.8 (0.5-1.0) | -1.7 (-1.98--1.41) |  | 1,898 (1,381-2,527) | 49.8 | 38 (28-51) | -1.82 (-2.12--1.51) |
| Thailand | 371 (246-540) | 44.3 | 1.0 (0.7-1.5) | -0.25 (-0.88-0.38) |  | 17,974 (12,084-26,099) | 42.0 | 50 (34-73) | -0.40 (-1.05-0.27) |
| Timor-Leste | 4 (1-6) | 126.0 | 0.6 (0.2-1.0) | 1.1 (0.77-1.43) |  | 202 (55-305) | 122.4 | 31 (8-47) | 1.05 (0.71-1.38) |
| Togo | 15 (9-23) | 205.0 | 0.4 (0.2-0.6) | 0.77 (0.68-0.86) |  | 743 (456-1,082) | 201.8 | 19 (12-28) | 0.73 (0.64-0.81) |
| Tokelau | 0 (0-0) | 13.6 | 0.8 (0.4-1.3) | 0.62 (0.46-0.77) |  | 0 (0-0) | 12.5 | 39 (22-62) | 0.60 (0.48-0.73) |
| Tonga | 0 (0-0) | 44.3 | 0.4 (0.2-0.6) | 0.96 (0.91-1.01) |  | 9 (6-13) | 44.3 | 18 (12-27) | 0.94 (0.89-1.00) |
| Trinidad and Tobago | 6 (4-9) | 37.5 | 0.9 (0.5-1.3) | 0.13 (-0.17-0.43) |  | 294 (179-429) | 37.4 | 42 (26-62) | 0.13 (-0.16-0.42) |
| Tunisia | 29 (17-42) | 128.1 | 0.5 (0.3-0.7) | 1.34 (1.22-1.47) |  | 1,383 (806-2,012) | 125.1 | 23 (13-33) | 1.29 (1.18-1.41) |
| Turkey | 295 (182-407) | 38.0 | 0.6 (0.4-0.9) | -0.76 (-1.13--0.39) |  | 14,322 (8,956-19,842) | 37.8 | 31 (20-43) | -0.76 (-1.14--0.38) |
| Turkmenistan | 16 (11-22) | 32.2 | 0.6 (0.4-0.8) | -1.06 (-1.75--0.37) |  | 808 (568-1,107) | 27.9 | 31 (22-42) | -1.17 (-1.89--0.45) |
| Tuvalu | 0 (0-0) | 38.1 | 0.9 (0.5-1.3) | -0.08 (-0.21-0.05) |  | 3 (2-4) | 38.6 | 43 (26-63) | -0.06 (-0.18-0.05) |
| Uganda | 94 (62-134) | 404.9 | 0.5 (0.3-0.7) | 2.22 (1.92-2.52) |  | 4,606 (3,023-6,586) | 407.4 | 24 (16-35) | 2.22 (1.90-2.53) |
| Ukraine | 298 (196-401) | -28.9 | 1.4 (0.9-1.9) | -2.33 (-3.15--1.5) |  | 14,255 (9,394-19,110) | -29.3 | 69 (45-92) | -2.34 (-3.17--1.51) |
| United Arab Emirates | 45 (26-69) | 819 | 0.6 (0.4-1.0) | 0.66 (-0.14-1.47) |  | 2,136 (1,221-3,285) | 804.3 | 31 (18-47) | 0.72 (-0.04-1.49) |
| UK | 226 (162-278) | -15.1 | 0.7 (0.5-0.9) | -0.82 (-1.05--0.59) |  | 11,105 (7,958-13,604) | -12.3 | 36 (26-45) | -0.70 (-0.93--0.47) |
| Tanzania | 123 (80-183) | 210.5 | 0.5 (0.3-0.7) | 1.07 (0.93-1.22) |  | 5,970 (3,866-8,905) | 208.6 | 22 (15-33) | 1.04 (0.91-1.18) |
| USA | 1,390 (1,010-1,694) | 26.5 | 0.9 (0.7-1.1) | 0.52 (0.36-0.67) |  | 67,885 (49,486-83,074) | 26.1 | 45 (33-55) | 0.51 (0.37-0.65) |
| United States Virgin Islands | 1 (0-1) | -6.8 | 1.3 (0.7-1.9) | 0.59 (0.49-0.69) |  | 26 (15-39) | -8.5 | 59 (34-89) | 0.47 (0.37-0.57) |
| Uruguay | 20 (15-25) | -3.3 | 1.2 (0.9-1.5) | -0.59 (-0.66--0.52) |  | 967 (709-1,205) | -2.3 | 59 (43-73) | -0.54 (-0.6--0.47) |
| Uzbekistan | 109 (76-142) | 79.3 | 0.6 (0.4-0.8) | -1.37 (-1.91--0.82) |  | 5,452 (3,793-7,092) | 73.5 | 30 (21-39) | -1.50 (-2.04--0.96) |
| Vanuatu | 1 (1-2) | 180.3 | 0.7 (0.5-1.1) | 0.7 (0.57-0.84) |  | 53 (32-77) | 179.3 | 36 (22-53) | 0.68 (0.54-0.81) |
| Venezuela | 106 (70-149) | 158.4 | 0.7 (0.5-1.0) | 1.15 (0.85-1.45) |  | 5,146 (3,413-7,271) | 154.3 | 36 (24-51) | 1.11 (0.80-1.41) |
| Vietnam | 697 (465-983) | 350.8 | 1.3 (0.9-1.9) | 4.08 (3.84-4.33) |  | 33,098 (21,854-46,535) | 336.7 | 62 (41-88) | 3.94 (3.72-4.17) |
| Yemen | 70 (43-105) | 264.6 | 0.4 (0.3-0.7) | 1.02 (0.89-1.15) |  | 3,397 (2,089-5,040) | 268.2 | 22 (13-32) | 1.10 (0.96-1.24) |
| Zambia | 67 (43-95) | 259.5 | 0.7 (0.5-1.1) | 1.06 (0.98-1.15) |  | 3,240 (2,101-4,636) | 258.7 | 36 (23-51) | 1.06 (0.98-1.13) |
| Zimbabwe | 59 (37-84) | 234.5 | 0.8 (0.5-1.1) | 2.88 (2.54-3.22) |  | 2,833 (1,776-4,077) | 231.9 | 38 (24-54) | 2.88 (2.54-3.23) |

**Supplementary Table S2. Early-onset colorectal cancer-related DALYs burden attributable to diet low in milk in 1990 and 2019, and its temporal trends from 1990 to 2019**

| Characteristics | 1990 | |  | 2019 | |  | 1990~2019 | |
| --- | --- | --- | --- | --- | --- | --- | --- | --- |
|  | DALYs  No. (95% UI) | ASR per 100,000  No. (95% UI) |  | DALYs  No. (95% UI) | ASR per 100,000  No. (95% UI) |  | Percent change in absolute number (%) | EAPC  No. (95% CI) |
| Overall | 384,768 (253,225-519,876) | 14.2 (9.3-19.2) |  | 704,982 (472,583-940,554) | 17.9 (12.0-23.9) |  | 83.2 | 0.75 (0.69-0.80) |
| Sex |  |  |  |  |  |  |  |  |
| Male | 205,385 (133,739-278,618) | 14.9 (9.7-20.3) |  | 419,819 (280,010-564,384) | 21.1 (14.1-28.4) |  | 104.4 | 1.26 (1.13-1.40) |
| Female | 179,383 (117,103-243,661) | 13.4 (8.8-18.2) |  | 285,163 (184,134-380,071) | 14.7 (9.5-19.5) |  | 59.0 | 0.04 (−0.10-0.19) |
| Socio-demographic index |  |  |  |  |  |  |  |  |
| High | 74,801 (41,955-107,504) | 17.4 (9.8-25.0) |  | 75,053 (43,038-108,458) | 16.0 (9.2-23.1) |  | 0.3 | −0.43 (−0.48-−0.38) |
| High -middle | 98,816 (59,863-137,271) | 16.3 (9.9-22.7) |  | 169,276 (109,209-230,606) | 23.2 (15.0-31.7) |  | 71.3 | 1.00 (0.92-1.08) |
| Middle | 135,342 (92,605-178,781) | 15 (10.3-19.8) |  | 275,893 (187,379-364,380) | 21.9 (14.9-28.9) |  | 103.8 | 1.33 (1.24-1.43) |
| Low-middle | 55,753 (37,721-75,196) | 10.32 (7.0-13.9) |  | 134,852 (90,514-180,569) | 14.5 (9.7-19.4) |  | 141.9 | 1.14 (1.05-1.23) |
| Low | 19,849 (13,055-27,565) | 8.54 (5.6-11.9) |  | 49,492 (32,133-67,636) | 9.2 (6.0-12.5) |  | 149.3 | 0.22 (0.13-0.31) |
| GBD regions |  |  |  |  |  |  |  |  |
| High-income Asia Pacific | 23,364 (15,164-31,755) | 25.2 (16.3-34.2) |  | 17,153 (10,938-23,346) | 21.1 (13.5-28.8) |  | −26.6 | −0.82 (−0.94-−0.69) |
| Central Asia | 4,148 (2,070−6,240) | 12.4 (6.2-18.7) |  | 5,110 (2,578-7,851) | 10.5 (5.3-16.1) |  | 23.2 | −1.55 (−1.88-−1.23) |
| East Asia | 134,236 (89,975-180,627) | 19.4 (13.0-26.2) |  | 252,424 (166,296-346,386) | 33.8 (22.3-46.4) |  | 88.0 | 2.01 (1.89-2.13) |
| South Asia | 43,062 (29,120-57,930) | 8.1 (5.5-10.9) |  | 111,473 (73,949-148,698) | 11.4 (7.6-15.3) |  | 158.9 | 1.05 (0.93-1.16) |
| Southeast Asia | 43,289 (30,844-56,501) | 18.3 (13.1-23.9) |  | 98,078 (67,625-131,382) | 27.1 (18.7-36.3) |  | 126.6 | 1.15 (1.03-1.28) |
| Australasia | 1,444 (559-2,331) | 13.4 (5.2-21.6) |  | 894 (272-1,754) | 6.6 (2.0-13.0) |  | −38.1 | −2.97 (−3.34-−2.6) |
| Caribbean | 2,622 (1,721-3,505) | 14.4 (9.4-19.2) |  | 4,770 (2,983-6,829) | 20.0 (12.5-28.6) |  | 81.9 | 1.11 (1.05-1.17) |
| Central Europe | 12,496 (6,084-18,677) | 20.5 (10.0-30.6) |  | 10,783 (5,681-16,302) | 20.5 (10.8-30.9) |  | −13.7 | −0.56 (−0.73-−0.39) |
| Eastern Europe | 13,558 (4,645-22,775) | 12.3 (4.2-20.6) |  | 16,890 (7,205-27,193) | 17.2 (7.4-27.7) |  | 24.6 | 0.34 (−0.17-0.84) |
| Western Europe | 29,872 (14,672-44,894) | 15.4 (7.6-23.2) |  | 23,131 (10,888-35,330) | 12.1 (5.7-18.5) |  | −22.6 | −1.03 (−1.17-−0.89) |
| Andean Latin America | 1,665 (1,087-2,289) | 8.9 (5.8-12.3) |  | 4,381 (2,594-6,397) | 13.2 (7.8-19.3) |  | 163.1 | 1.53 (1.33-1.73) |
| Central Latin America | 6,655 (4,110-9,177) | 8.2 (5.0-11.3) |  | 18,906 (10,918-27,447) | 14.4 (8.3-20.8) |  | 184.1 | 1.98 (1.92-2.05) |
| Southern Latin America | 4,219 (2,469-5,938) | 17.2 (10.1-24.2) |  | 7,491 (4,371-10,512) | 22.0 (12.8-30.9) |  | 77.6 | 0.84 (0.79-0.88) |
| Tropical Latin America | 8,939 (5,049-12,778) | 11.4 (6.4-16.3) |  | 18,332 (9,397-27,366) | 15.4 (7.9-23.0) |  | 105.1 | 0.95 (0.78-1.13) |
| North Africa and Middle East | 16,028 (9,865-23,124) | 9.9 (6.1-14.2) |  | 43,020 (26,519-60,857) | 12.9 (8.0-18.2) |  | 168.4 | 0.84 (0.58-1.09) |
| High-income North America | 19,438 (8,729-29,969) | 13.1 (5.9-20.2) |  | 21,670 (9,127-35,024) | 13.0 (5.5-21.0) |  | 11.5 | 0.01 (−0.07-0.09) |
| Oceania | 373 (245-522) | 11.8 (7.7-16.5) |  | 986 (616-1,444) | 14.5 (9.1-21.2) |  | 164.3 | 0.70 (0.68-0.73) |
| Central Sub-Saharan Africa | 2,858 (1,997-3,951) | 11.7 (8.2-16.2) |  | 7,261 (4,691-10,484) | 11.7 (7.6-16.9) |  | 154.1 | −0.09 (−0.31-0.13) |
| Eastern Sub-Saharan Africa | 7,249 (4,514-10,383) | 8.7 (5.4-12.5) |  | 19,239 (11,931-27,567) | 9.7 (6.0-13.9) |  | 165.4 | 0.28 (0.12-0.44) |
| Southern Sub-Saharan Africa | 3,458 (2,233-4,798) | 13.3 (8.6-18.4) |  | 6,171 (3,768-8,877) | 14.6 (8.9-21.0) |  | 78.5 | 0.11 (−0.12-0.33) |
| Western Sub-Saharan Africa | 5,796 (3,777-8,080) | 6.8 (4.4-9.5) |  | 16,820 (10,828-23,746) | 7.8 (5.0-11.1) |  | 190.2 | 0.67 (0.57-0.77) |

**Supplementary Table S3. Early-onset colorectal cancer-related DALYs burden attributable to a diet low in** **whole grains in 1990 and 2019, and its temporal trends from 1990 to 2019**

| Characteristics | 1990 | |  | 2019 | |  | 1990~2019 | |
| --- | --- | --- | --- | --- | --- | --- | --- | --- |
|  | DALYs  No. (95% UI) | ASR per 100,000  No. (95% UI) |  | DALYs  No. (95% UI) | ASR per 100,000  No. (95% UI) |  | Percent change in absolute number (%) | EAPC  No. (95% CI) |
| Overall | 377,401 (148,273-493,906) | 13.9 (5.5-18.2) |  | 647,577 (250,407-858,385) | 16.5 (6.4-21.8) |  | 71.6 | 0.44 (0.36-0.53) |
| Sex |  |  |  |  |  |  |  |  |
| Male | 203,492 (79,476-265,936) | 14.8 (5.8-19.4) |  | 386,740 (150,174-521,565) | 19.4 (7.5-26.2) |  | 90.1 | 0.92 (0.80-1.05) |
| Female | 173,909 (68,445-227,035) | 13.0 (5.1-17.0) |  | 260,837 (101,289-350,610) | 13.4 (5.2-18.0) |  | 50.0 | −0.21 (−0.37-−0.06) |
| Socio-demographic index |  |  |  |  |  |  |  |  |
| High | 83,202 (30,994-110,326) | 19.3 (7.2-25.7) |  | 92,360 (34,137-121,867) | 19.7 (7.3-25.9) |  | 11.0 | −0.03 (−0.13-0.07) |
| High -middle | 123,582 (50,710-157,446) | 20.4 (8.4-26) |  | 177,792 (69,644-233,823) | 24.4 (9.6-32.1) |  | 43.9 | 0.19 (0.05-0.33) |
| Middle | 109,537 (41,403-145,251) | 12.1 (4.6-16.1) |  | 226,146 (87,858-306,608) | 17.9 (7-24.3) |  | 106.5 | 1.37 (1.25-1.49) |
| Low-middle | 44,242 (17,255-59,668) | 8.2 (3.2-11) |  | 107,371 (41,319-145,195) | 11.5 (4.4-15.6) |  | 142.7 | 1.15 (1.06-1.24) |
| Low | 16,651 (6,717-22,967) | 7.2 (2.9-9.9) |  | 43,543 (17,211-58,505) | 8.1 (3.2-10.8) |  | 161.5 | 0.41 (0.36-0.46) |
| GBD regions |  |  |  |  |  |  |  |  |
| High-income Asia Pacific | 18,254 (6,775-25,059) | 19.7 (7.3-27) |  | 14,658 (5,416-19,534) | 18.1 (6.7-24.1) |  | −19.7 | −0.46 (−0.57-−0.35) |
| Central Asia | 8,044 (3,987-9,911) | 24.1 (12-29.7) |  | 9,874 (4,771-12,568) | 20.2 (9.8-25.7) |  | 22.7 | −1.22 (−1.44-−1.01) |
| East Asia | 114,094 (42,325-152,292) | 16.5 (6.1-22.1) |  | 214,737 (80,281-300,391) | 28.8 (10.8-40.2) |  | 88.2 | 1.95 (1.81-2.09) |
| South Asia | 32,156 (12,509-43,620) | 6.1 (2.4-8.2) |  | 85,777 (34,457-116,916) | 8.8 (3.5-12) |  | 166.8 | 1.19 (1.09-1.29) |
| Southeast Asia | 26,742 (9,985-37,658) | 11.3 (4.2-15.9) |  | 64,356 (24,600-94,867) | 17.8 (6.8-26.2) |  | 140.7 | 1.39 (1.23-1.55) |
| Australasia | 2,259 (839-3,028) | 20.9 (7.8-28.1) |  | 2,571 (938-3,487) | 19 (6.9-25.8) |  | 13.8 | −0.37 (−0.47-−0.27) |
| Caribbean | 2,396 (905-3,133) | 13.1 (5-17.2) |  | 4,195 (1,560-5,934) | 17.5 (6.5-24.8) |  | 75.1 | 0.98 (0.92-1.04) |
| Central Europe | 17,143 (6,708-21,945) | 28.1 (11-36) |  | 14,280 (5,332-19,332) | 27.1 (10.1-36.7) |  | −16.7 | −0.58 (−0.74-−0.43) |
| Eastern Europe | 32,222 (14,250-40,804) | 29.2 (12.9-37) |  | 28,422 (10,742-37,551) | 29.0 (11.0-38.3) |  | −11.8 | −1.10 (−1.50-−0.69) |
| Western Europe | 40,447 (15,986-51,936) | 20.9 (8.3-26.9) |  | 35,462 (14,045-45,418) | 18.6 (7.4-23.8) |  | −12.3 | −0.54 (−0.72-−0.35) |
| Andean Latin America | 1,493 (564-1,987) | 8 (3-10.7) |  | 3,817 (1,354-5,503) | 11.5 (4.1-16.6) |  | 155.7 | 1.40 (1.20-1.60) |
| Central Latin America | 5,680 (2,119-7,597) | 7 (2.6-9.3) |  | 16,852 (6,274-23,521) | 12.8 (4.8-17.9) |  | 196.7 | 2.14 (2.08-2.20) |
| Southern Latin America | 5,239 (2,277-6,585) | 21.4 (9.3-26.9) |  | 8,234 (3,083-10,744) | 24.2 (9.1-31.6) |  | 57.2 | 0.39 (0.29-0.48) |
| Tropical Latin America | 8,955 (3,375-11,817) | 11.4 (4.3-15) |  | 19,667 (7,298-26,350) | 16.5 (6.1-22.1) |  | 119.6 | 1.27 (1.09-1.44) |
| North Africa and Middle East | 20,264 (9,643-27,228) | 12.5 (5.9-16.8) |  | 50,761 (23,083-67,315) | 15.2 (6.9-20.2) |  | 150.5 | 0.59 (0.31-0.87) |
| High-income North America | 26,817 (9,867-35,389) | 18 (6.6-23.8) |  | 34,549 (12,925-45,339) | 20.7 (7.8-27.2) |  | 28.8 | 0.52 (0.37-0.67) |
| Oceania | 335 (128-465) | 10.6 (4-14.7) |  | 874 (316-1,286) | 12.9 (4.6-18.9) |  | 160.9 | 0.67 (0.64-0.70) |
| Central Sub-Saharan Africa | 1,587 (600-2,366) | 6.5 (2.5-9.7) |  | 4,238 (1,503-6,367) | 6.8 (2.4-10.2) |  | 167.0 | 0.09 (−0.08-0.26) |
| Eastern Sub-Saharan Africa | 6,046 (2,385-8,544) | 7.3 (2.9-10.3) |  | 16,061 (5,744-22,939) | 8.1 (2.9-11.5) |  | 165.6 | 0.28 (0.13-0.43) |
| Southern Sub-Saharan Africa | 2,521 (960-3,551) | 9.7 (3.7-13.6) |  | 4,750 (1,768-6,771) | 11.2 (4.2-16) |  | 88.4 | 0.31 (0.07-0.56) |
| Western Sub-Saharan Africa | 4,706 (1,798-6,714) | 5.5 (2.1-7.9) |  | 13,442 (5,104-18,788) | 6.3 (2.4-8.7) |  | 185.6 | 0.56 (0.47-0.65) |

**Supplementary Table S4. Early-onset colorectal cancer-related DALYs burden attributable to a diet** **low in calcium in 1990 and 2019, and its temporal trends from 1990 to 2019**

| Characteristics | 1990 | |  | 2019 | |  | 1990~2019 | |
| --- | --- | --- | --- | --- | --- | --- | --- | --- |
|  | DALYs  No. (95% UI) | ASR per 100,000  No. (95% UI) |  | DALYs  No. (95% UI) | ASR per 100,000  No. (95% UI) |  | Percent change in absolute number (%) | EAPC  No. (95% CI) |
| Overall | 378,672 (293,975-494,374) | 14.0 (10.8-18.2) |  | 610,252 (448,848-809,957) | 15.5 (11.4-20.6) |  | 61.2 | 0.28 (0.17-0.39) |
| Sex |  |  |  |  |  |  |  |  |
| Male | 210,326 (162,854-274,355) | 15.3 (11.9-20.0) |  | 376,421 (274,187-501,382) | 18.9 (13.8-25.2) |  | 79.0 | 0.79 (0.62-0.95) |
| Female | 168,346 (126,114-222,086) | 12.6 (9.4-16.6) |  | 233,830 (171,534-313,738) | 12.0 (8.8-16.1) |  | 38.9 | −0.45 (−0.62-−0.28) |
| Socio-demographic index |  |  |  |  |  |  |  |  |
| High | 43,032 (26,014-65,074) | 10 (6-15.1) |  | 40,605 (24,020-61,522) | 8.6 (5.1-13.1) |  | −5.6 | −0.41 (−0.48-−0.33) |
| High -middle | 93,275 (65,868-130,052) | 15.4 (10.9-21.5) |  | 121,511 (78,236-178,800) | 16.7 (10.7-24.5) |  | 30.3 | −0.10 (−0.27-0.06) |
| Middle | 156,717 (125,660-195,370) | 17.4 (13.9-21.7) |  | 267,181 (202,688-345,913) | 21.2 (16.1-27.4) |  | 70.5 | 0.68 (0.55-0.81) |
| Low-middle | 61,216 (48,320-77,424) | 11.3 (8.9-14.3) |  | 123,248 (92,189-160,482) | 13.2 (9.9-17.2) |  | 101.3 | 0.51 (0.38-0.64) |
| Low | 24,226 (18,494-31,349) | 10.4 (8-13.5) |  | 57,281 (44,539-72,245) | 10.6 (8.2-13.4) |  | 136.4 | 0.03 (-0.03-0.08) |
| GBD regions |  |  |  |  |  |  |  |  |
| High-income Asia Pacific | 18,432 (12,434-25,755) | 19.8 (13.4-27.7) |  | 13,280 (8,780-18,681) | 16.4 (10.8-23) |  | −28 | −0.67 (−0.79-−0.55) |
| Central Asia | 4,326 (2,786-6,374) | 13 (8.4-19.1) |  | 4,142 (2,459-6,408) | 8.5 (5-13.1) |  | −4.3 | −2.62 (−3.02-−2.23) |
| East Asia | 152,119 (119,224-195,940) | 22 (17.3-28.4) |  | 205,682 (139,136-285,309) | 27.6 (18.6-38.2) |  | 35.2 | 0.85 (0.68-1.02) |
| South Asia | 40,718 (31,477-52,315) | 7.7 (5.9-9.9) |  | 86,170 (61,176-117,295) | 8.8 (6.3-12) |  | 111.6 | 0.46 (0.35-0.57) |
| Southeast Asia | 54,007 (41,116-67,257) | 22.8 (17.4-28.5) |  | 123,430 (95,172-156,368) | 34.1 (26.3-43.2) |  | 128.5 | 1.22 (1.06-1.38) |
| Australasia | 972 (476-1,714) | 9 (4.4-15.9) |  | 1,062 (559-1,808) | 7.9 (4.1-13.4) |  | 9.3 | −0.46 (−0.61-−0.31) |
| Caribbean | 2,389 (1,759-3,117) | 13.1 (9.6-17.1) |  | 4,170 (2,913-5,738) | 17.4 (12.2-24) |  | 74.6 | 0.79 (0.69-0.9) |
| Central Europe | 7,570 (4,017-12,745) | 12.4 (6.6-20.9) |  | 5,351 (2,724-9,129) | 10.2 (5.2-17.3) |  | −29.3 | −1.29 (−1.54-−1.04) |
| Eastern Europe | 14,143 (7,705-23,424) | 12.8 (7-21.2) |  | 11,675 (5,892-20,276) | 11.9 (6-20.7) |  | −17.5 | −1.84 (−2.46-−1.22) |
| Western Europe | 13,183 (6,572-22,885) | 6.8 (3.4-11.8) |  | 9,334 (4,510-16,171) | 4.9 (2.4-8.5) |  | −29.2 | −1.21 (−1.34-−1.08) |
| Andean Latin America | 1,911 (1,457-2,444) | 10.3 (7.8-13.1) |  | 4,153 (2,758-5,857) | 12.5 (8.3-17.7) |  | 117.3 | 0.72 (0.51-0.93) |
| Central Latin America | 7,046 (5,335-9,064) | 8.6 (6.5-11.1) |  | 17,389 (11,648-24,886) | 13.2 (8.8-18.9) |  | 146.8 | 1.44 (1.35-1.52) |
| Southern Latin America | 3,269 (2,065-4,817) | 13.3 (8.4-19.7) |  | 4,919 (2,894-7,693) | 14.5 (8.5-22.6) |  | 50.5 | 0.53 (0.42-0.64) |
| Tropical Latin America | 9,056 (6,505-12,107) | 11.5 (8.3-15.4) |  | 13,515 (7,922-20,915) | 11.3 (6.6-17.5) |  | 49.2 | −0.41 (−0.55-−0.27) |
| North Africa and Middle East | 15,649 (11,155-21,764) | 9.6 (6.9-13.4) |  | 36,353 (25,437-50,292) | 10.9 (7.6-15.1) |  | 132.3 | 0.30 (0.06-0.55) |
| High-income North America | 8,958 (4,313-15,769) | 6 (2.9-10.6) |  | 9,514 (4,590-16,552) | 5.7 (2.8-9.9) |  | 6.2 | 0.44 (0.19-0.69) |
| Oceania | 456 (329-613) | 14.4 (10.4-19.4) |  | 1,230 (883-1,737) | 18.1 (13-25.5) |  | 169.7 | 0.82 (0.78-0.85) |
| Central Sub-Saharan Africa | 2,725 (1,978-3,715) | 11.2 (8.1-15.2) |  | 7,126 (4,749-10,026) | 11.5 (7.6-16.1) |  | 161.5 | −0.01 (−0.18-0.17) |
| Eastern Sub-Saharan Africa | 9,796 (7,142-12,909) | 11.8 (8.6-15.6) |  | 24,250 (18,256-31,709) | 12.2 (9.2-15.9) |  | 147.6 | −0.02 (−0.17-0.13) |
| Southern Sub-Saharan Africa | 4,625 (3,735-5,696) | 17.7 (14.3-21.8) |  | 8,379 (6,545-10,614) | 19.8 (15.5-25.1) |  | 81.2 | 0.14 (−0.11-0.38) |
| Western Sub-Saharan Africa | 7,323 (5,505-9,682) | 8.6 (6.5-11.4) |  | 19,128 (14,095-25,309) | 8.9 (6.6-11.8) |  | 161.2 | 0.18 (0.09-0.27) |

**Supplementary Table S5. Early-onset colorectal cancer-related DALYs burden attributable to** **a diet low in fiber in 1990 and 2019, and its temporal trends from 1990 to 2019**

| Characteristics | 1990 | |  | 2019 | |  | 1990~2019 | |
| --- | --- | --- | --- | --- | --- | --- | --- | --- |
|  | DALYs  No. (95% UI) | ASR per 100,000  No. (95% UI) |  | DALYs  No. (95% UI) | ASR per 100,000  No. (95% UI) |  | Percent change in absolute number (%) | EAPC  No. (95% CI) |
| Overall | 75,186 (31,131-131,071) | 2.8 (1.1-4.8) |  | 96,951 (39,222-176,401) | 2.5 (1-4.5) |  | 28.9 | −0.57 (−0.76-−0.38) |
| Sex |  |  |  |  |  |  |  |  |
| Male | 36,193 (13,843-55,657) | 2.6 (1.0-4.0) |  | 60,141 (19,757-94,797) | 3.0 (1.0-4.8) |  | 66.2 | −0.13 (−0.34-0.09) |
| Female | 33,716 (12,964-52,096) | 2.5 (1.0-3.9) |  | 48,112 (16,781-74,486) | 2.5 (0.9-3.8) |  | 42.7 | −1.11 (−1.34-−0.89) |
| Socio-demographic index |  |  |  |  |  |  |  |  |
| High | 15,827 (5,999-29,208) | 3.7 (1.4-6.8) |  | 13,589 (4,925-26,328) | 2.9 (1.0-5.6) |  | −14.1 | −0.79 (−0.92-−0.66) |
| High -middle | 17,013 (6,639-32,197) | 2.8 (1.1-5.3) |  | 19,369 (7,162-39,018) | 2.7 (1.0-5.4) |  | 13.8 | −0.63 (−0.87-−0.39) |
| Middle | 27,769 (11,943-45,935) | 3.1 (1.3-5.1) |  | 38,180 (16,131-66,455) | 3.0 (1.3-5.3) |  | 37.5 | −0.19 (−0.39-0.01) |
| Low-middle | 12,266 (5,641-20,063) | 2.3 (1-3.7) |  | 20,444 (8,507-36,230) | 2.2 (0.9-3.9) |  | 66.7 | −0.28 (−0.49-−0.06) |
| Low | 2,270 (895-4,450) | 1 (0.4-1.9) |  | 5,308 (2,028-9,805) | 1 (0.4-1.8) |  | 133.8 | 0 (−0.16-0.17) |
| GBD regions |  |  |  |  |  |  |  |  |
| High-income Asia Pacific | 3,271 (1,223-6,352) | 3.5 (1.3-6.8) |  | 3,341 (1,245-5,856) | 4.1 (1.5-7.2) |  | 2.1 | 0.69 (0.47-0.91) |
| Central Asia | 1,209 (444-2,225) | 3.6 (1.3-6.7) |  | 981 (343-1,976) | 2.0 (0.7-4.0) |  | −18.9 | −3.44 (−3.98-−2.89) |
| East Asia | 25,154 (9,674-45,183) | 3.6 (1.4-6.5) |  | 23,911 (8,465-49,833) | 3.2 (1.1-6.7) |  | −4.9 | −0.43 (−0.69-−0.17) |
| South Asia | 8,465 (3,465-14,622) | 1.6 (0.7-2.8) |  | 15,644 (6,124-29,363) | 1.6 (0.6-3.0) |  | 84.8 | −0.11 (−0.32-0.1) |
| Southeast Asia | 13,791 (7,059-20,065) | 5.8 (3-8.5) |  | 26,159 (12,038-42,068) | 7.2 (3.3-11.6) |  | 89.7 | 0.53 (0.35-0.70) |
| Australasia | 530 (195-953) | 4.9 (1.8-8.8) |  | 457 (160-866) | 3.4 (1.2-6.4) |  | −13.8 | −1.59 (−1.79-−1.39) |
| Caribbean | 458 (167-849) | 2.5 (0.9-4.7) |  | 543 (198-1,091) | 2.3 (0.8-4.6) |  | 18.6 | −0.56 (−0.78-−0.34) |
| Central Europe | 1,936 (676-4,068) | 3.2 (1.1-6.7) |  | 1,481 (534-3,031) | 2.8 (1.0-5.8) |  | −23.5 | −0.9 (−1.12-−0.67) |
| Eastern Europe | 2,693 (962-5,920) | 2.4 (0.9-5.4) |  | 2,951 (1,039-6,320) | 3.0 (1.1-6.4) |  | 9.6 | −0.87 (−1.59-−0.15) |
| Western Europe | 5,801 (2,128-11,474) | 3 (1.1-5.9) |  | 4,753 (1,712-9,787) | 2.5 (0.9-5.1) |  | −18.1 | −0.74 (−0.89-−0.59) |
| Andean Latin America | 348 (126-632) | 1.9 (0.7-3.4) |  | 707 (278-1,246) | 2.1 (0.8-3.8) |  | 103.2 | 0.56 (0.39-0.74) |
| Central Latin America | 774 (308-1,419) | 0.9 (0.4-1.7) |  | 1,869 (711-3,731) | 1.4 (0.5-2.8) |  | 141.5 | 1.57 (1.48-1.66) |
| Southern Latin America | 1,025 (375-1,801) | 4.2 (1.5-7.4) |  | 1,584 (577-2,928) | 4.7 (1.7-8.6) |  | 54.5 | 0.64 (0.48-0.80) |
| Tropical Latin America | 1,842 (698-3,301) | 2.3 (0.9-4.2) |  | 2,944 (1,096-5,835) | 2.5 (0.9-4.9) |  | 59.8 | −0.27 (−0.42-−0.11) |
| North Africa and Middle East | 1,088 (442-2,458) | 0.7 (0.3-1.5) |  | 2,983 (1,232-5,939) | 0.9 (0.4-1.8) |  | 174.2 | 0.99 (0.81-1.16) |
| High-income North America | 5,620 (2,116-10,249) | 3.8 (1.4-6.9) |  | 4,222 (1,532-8,548) | 2.5 (0.9-5.1) |  | −24.9 | −1.17 (−1.27-−1.06) |
| Oceania | 12 (6-27) | 0.4 (0.2-0.9) |  | 19 (11-40) | 0.3 (0.2-0.6) |  | 58.3 | −1.19 (−1.37-−1.01) |
| Central Sub-Saharan Africa | 172 (58-389) | 0.7 (0.2-1.6) |  | 558 (192-1,145) | 0.9 (0.3-1.8) |  | 224.4 | 0.70 (0.51-0.89) |
| Eastern Sub-Saharan Africa | 461 (185-1,039) | 0.6 (0.2-1.3) |  | 945 (413-1,840) | 0.5 (0.2-0.9) |  | 105.0 | −0.82 (−0.96-−0.68) |
| Southern Sub-Saharan Africa | 204 (79-481) | 0.8 (0.3-1.8) |  | 429 (164-927) | 1 (0.4-2.2) |  | 110.3 | 0.69 (0.33-1.05) |
| Western Sub-Saharan Africa | 332 (138-709) | 0.4 (0.2-0.8) |  | 471 (254-885) | 0.2 (0.1-0.4) |  | 41.9 | −2.15 (−2.53-−1.77) |

**Supplementary Table S6. Early-onset colorectal cancer-related DALYs burden attributable to a diet** **high in red meat in 1990 and 2019, and its temporal trends from 1990 to 2019**

| Characteristics | 1990 | |  | 2019 | |  | 1990~2019 | |
| --- | --- | --- | --- | --- | --- | --- | --- | --- |
|  | DALYs  No. (95% UI) | ASR per 100,000  No. (95% UI) |  | DALYs  No. (95% UI) | ASR per 100,000  No. (95% UI) |  | Percent change in absolute number (%) | EAPC  No. (95% CI) |
| Overall | 114,373 (31,317-216,748) | 4.2 (1.2-8) |  | 226,827 (68,817-404,990) | 5.8 (1.7-10.3) |  | 98.3 | 1.12 (1.05-1.2) |
| Sex |  |  |  |  |  |  |  |  |
| Male | 62,023 (16,439-118,288) | 4.5 (1.2-8.6) |  | 141,618 (42,907-249,575) | 7.1 (2.2-12.5) |  | 128.3 | 1.74 (1.56-1.93) |
| Female | 52,350 (14,401-98,445) | 3.9 (1.1-7.4) |  | 85,209 (24,818-155,674) | 4.4 (1.3-8.0) |  | 62.8 | 0.22 (0.12-0.33) |
| Socio-demographic index |  |  |  |  |  |  |  |  |
| High | 42,606 (14,794-71,442) | 9.9 (3.4-16.6) |  | 46,453 (15,728-77,044) | 9.9 (3.3-16.4) |  | 9.0 | -0.03 (-0.15-0.08) |
| High -middle | 41,114 (10,938-76,178) | 6.8 (1.8-12.6) |  | 78,571 (25,796-132,488) | 10.8 (3.5-18.2) |  | 91.1 | 1.35 (1.19-1.51) |
| Middle | 22,718 (3,416-54,512) | 2.5 (0.4-6) |  | 76,548 (20,929-143,242) | 6.1 (1.7-11.4) |  | 236.9 | 3.44 (3.29-3.6) |
| Low-middle | 6,021 (1,239-13,581) | 1.1 (0.2-2.5) |  | 19,834 (5,358-39,696) | 2.1 (0.6-4.3) |  | 229.4 | 2.51 (2.34-2.68) |
| Low | 1,869 (255-4,820) | 0.8 (0.1-2.1) |  | 5,347 (771-13,529) | 1.0 (0.1-2.5) |  | 186.1 | 0.82 (0.71-0.92) |
| GBD regions |  |  |  |  |  |  |  |  |
| High-income Asia Pacific | 4,121 (399-10,693) | 4.4 (0.4-11.5) |  | 4,569 (974-9,500) | 5.6 (1.2-11.7) |  | 10.9 | 0.68 (0.5-0.86) |
| Central Asia | 2,257 (642-4,192) | 6.8 (1.9-12.6) |  | 2,892 (864-5,387) | 5.9 (1.8-11) |  | 28.1 | -0.75 (-1.18--0.31) |
| East Asia | 28,348 (4,408-66,983) | 4.1 (0.6-9.7) |  | 104,591 (33,372-181,771) | 14.0 (4.5-24.4) |  | 269 | 4.74 (4.57-4.91) |
| South Asia | 2,206 (655-4,716) | 0.4 (0.1-0.9) |  | 6,217 (1,532-14,123) | 0.6 (0.2-1.4) |  | 181.8 | 1.35 (1.25-1.46) |
| Southeast Asia | 3,745 (423-9,686) | 1.6 (0.2-4.1) |  | 12,672 (1,574-30,963) | 3.5 (0.4-8.6) |  | 238.4 | 2.64 (2.54-2.75) |
| Australasia | 1,972 (983-2,895) | 18.3 (9.1-26.8) |  | 2,127 (1,013-3,124) | 15.7 (7.5-23.1) |  | 7.9 | -0.47 (-0.62--0.33) |
| Caribbean | 410 (40-1,055) | 2.2 (0.2-5.8) |  | 746 (74-1,950) | 3.1 (0.3-8.2) |  | 82 | 1.31 (1.23-1.39) |
| Central Europe | 6,446 (1,759-11,802) | 10.6 (2.9-19.3) |  | 6,704 (2,070-11,857) | 12.7 (3.9-22.5) |  | 4 | 0.59 (0.44-0.75) |
| Eastern Europe | 12,126 (3,525-21,431) | 11 (3.2-19.4) |  | 8,747 (1,927-17,943) | 8.9 (2-18.3) |  | -27.9 | -2.01 (-2.49--1.54) |
| Western Europe | 23,180 (8,634-36,825) | 12 (4.5-19) |  | 18,380 (6,465-29,593) | 9.6 (3.4-15.5) |  | -20.7 | -0.94 (-1.12--0.76) |
| Andean Latin America | 257 (29-653) | 1.4 (0.2-3.5) |  | 912 (134-2,161) | 2.8 (0.4-6.5) |  | 254.9 | 2.7 (2.55-2.86) |
| Central Latin America | 1,619 (255-3,645) | 2 (0.3-4.5) |  | 5,495 (1,108-11,925) | 4.2 (0.8-9.1) |  | 239.4 | 2.75 (2.67-2.83) |
| Southern Latin America | 3,129 (1,538-4,679) | 12.8 (6.3-19.1) |  | 5,791 (2,755-8,659) | 17.0 (8.1-25.4) |  | 85.1 | 0.9 (0.8-0.99) |
| Tropical Latin America | 3,249 (774-6,450) | 4.1 (1-8.2) |  | 14,396 (6,286-22,096) | 12.1 (5.3-18.5) |  | 343.1 | 3.78 (3.12-4.45) |
| North Africa and Middle East | 2,623 (249-7,187) | 1.6 (0.2-4.4) |  | 6,508 (593-17,540) | 2.0 (0.2-5.3) |  | 148.1 | 0.6 (0.25-0.95) |
| High-income North America | 16,233 (5,902-26,058) | 10.9 (4-17.5) |  | 19,723 (6,731-32,067) | 11.8 (4-19.2) |  | 21.5 | 0.33 (0.18-0.48) |
| Oceania | 67 (9-170) | 2.1 (0.3-5.4) |  | 153 (16-414) | 2.3 (0.2-6.1) |  | 128.4 | 0.02 (-0.06-0.1) |
| Central Sub-Saharan Africa | 217 (38-542) | 0.9 (0.2-2.2) |  | 549 (83-1,425) | 0.9 (0.1-2.3) |  | 153 | 0.09 (-0.13-0.31) |
| Eastern Sub-Saharan Africa | 794 (83-2,135) | 1 (0.1-2.6) |  | 2,296 (224-6,194) | 1.2 (0.1-3.1) |  | 189.2 | 0.69 (0.51-0.87) |
| Southern Sub-Saharan Africa | 768 (120-1,744) | 2.9 (0.5-6.7) |  | 1,489 (281-3,369) | 3.5 (0.7-8.0) |  | 93.9 | 0.71 (0.45-0.97) |
| Western Sub-Saharan Africa | 606 (58-1,629) | 0.7 (0.1-1.9) |  | 1,869 (180-5,099) | 0.9 (0.1-2.4) |  | 208.4 | 0.92 (0.8-1.04) |

**Supplementary Table S7. Early-onset colorectal cancer-related DALYs burden attributable to a diet** **high in processed meat in 1990 and 2019, and its temporal trends from 1990 to 2019**

| Characteristics | 1990 | |  | 2019 | |  | 1990~2019 | |
| --- | --- | --- | --- | --- | --- | --- | --- | --- |
|  | DALYs  No. (95% UI) | ASR per 100,000  No. (95% UI) |  | DALYs  No. (95% UI) | ASR per 100,000  No. (95% UI) |  | Percent change in absolute number (%) | EAPC  No. (95% CI) |
| Overall | 69,909 (27,013-107,074) | 2.6 (1-3.9) |  | 108,254 (37,041-167,675) | 2.8 (0.9-4.3) |  | 54.8 | 0.05 (−0.04-0.14) |
| Sex |  |  |  |  |  |  |  |  |
| Male | 36,193 (13,843-55,657) | 2.6 (1.0-4.0) |  | 60,141 (19,757-94,797) | 3.0 (1.0-4.8) |  | 66.2 | 0.35 (0.26-0.45) |
| Female | 33,716 (12,964-52,096) | 2.5 (1.0-3.9) |  | 48,112 (16,781-74,486) | 2.5 (0.9-3.8) |  | 42.7 | −0.31 (−0.42-−0.20) |
| Socio-demographic index |  |  |  |  |  |  |  |  |
| High | 32,652 (11,607-50,302) | 7.6 (2.7-11.7) |  | 36,032 (13,958-57,961) | 7.7 (3-12.3) |  | 10.4 | 0.01 (−0.17-0.20) |
| High -middle | 24,307 (9,131-39,605) | 4 (1.5-6.5) |  | 32,069 (8,858-50,494) | 4.4 (1.2-6.9) |  | 31.9 | −0.27 (−0.50-−0.04) |
| Middle | 7,118 (2,587-11,258) | 0.8 (0.3-1.2) |  | 22,194 (5,539-37,248) | 1.8 (0.4-3) |  | 211.8 | 3.10 (2.94-3.25) |
| Low-middle | 3,899 (1,621-6,047) | 0.7 (0.3-1.1) |  | 12,257 (4,553-18,966) | 1.3 (0.5-2) |  | 214.4 | 2.21 (2.16-2.26) |
| Low | 1,909 (437-3,326) | 0.8 (0.2-1.4) |  | 5,663 (1,293-9,631) | 1 (0.2-1.8) |  | 196.6 | 0.89 (0.84-0.95) |
| GBD regions |  |  |  |  |  |  |  |  |
| High-income Asia Pacific | 7,087 (2,184-10,746) | 7.6 (2.4-11.6) |  | 5,644 (1,815-8,624) | 7.0 (2.2-10.6) |  | −20.4 | −0.43 (−0.56-−0.29) |
| Central Asia | 1,692 (347-2,653) | 5.1 (1-8) |  | 2,125 (520-3,407) | 4.4 (1.1-7.0) |  | 25.6 | −1.04 (−1.29-−0.79) |
| East Asia | 6,012 (2,131-10,210) | 0.9 (0.3-1.5) |  | 21,286 (3,109-39,361) | 2.9 (0.4-5.3) |  | 254.1 | 4.67 (4.44-4.89) |
| South Asia | 3,107 (1,702-4,632) | 0.6 (0.3-0.9) |  | 10,559 (6,049-15,439) | 1.1 (0.6-1.6) |  | 239.8 | 2.13 (2.05-2.22) |
| Southeast Asia | 1,382 (609-2,118) | 0.6 (0.3-0.9) |  | 4,480 (1,516-7,458) | 1.2 (0.4-2.1) |  | 224.2 | 2.59 (2.47-2.72) |
| Australasia | 898 (272-1,354) | 8.3 (2.5-12.5) |  | 1,051 (348-1,596) | 7.8 (2.6-11.8) |  | 17.0 | −0.21 (−0.32-−0.10) |
| Caribbean | 214 (32-382) | 1.2 (0.2-2.1) |  | 410 (59-781) | 1.7 (0.2-3.3) |  | 91.6 | 1.45 (1.4-1.51) |
| Central Europe | 4,055 (901-6,345) | 6.6 (1.5-10.4) |  | 4,501 (1,266-7,094) | 8.5 (2.4-13.5) |  | 11.0 | 0.81 (0.71-0.92) |
| Eastern Europe | 11,944 (3,855-22,201) | 10.8 (3.5-20.1) |  | 9,055 (2,765-14,242) | 9.2 (2.8-14.5) |  | −24.2 | −1.66 (−2.15-−1.18) |
| Western Europe | 15,765 (5,704-24,572) | 8.2 (2.9-12.7) |  | 14,050 (5,184-21,968) | 7.4 (2.7-11.5) |  | −10.9 | −0.42 (−0.65-−0.19) |
| Andean Latin America | 89 (30-145) | 0.5 (0.2-0.8) |  | 324 (58-614) | 1.0 (0.2-1.9) |  | 264.0 | 2.87 (2.63-3.10) |
| Central Latin America | 721 (118-1,240) | 0.9 (0.1-1.5) |  | 2,487 (427-4,422) | 1.9 (0.3-3.4) |  | 244.9 | 2.82 (2.75-2.89) |
| Southern Latin America | 1,136 (239-1,839) | 4.6 (1-7.5) |  | 2,507 (695-3,920) | 7.4 (2.0-11.5) |  | 120.7 | 1.70 (1.54-1.86) |
| Tropical Latin America | 960 (132-1,716) | 1.2 (0.2-2.2) |  | 3,260 (400-5,608) | 2.7 (0.3-4.7) |  | 239.6 | 3.15 (3.02-3.29) |
| North Africa and Middle East | 1,090 (220-2,070) | 0.7 (0.1-1.3) |  | 3,359 (579-6,131) | 1.0 (0.2-1.8) |  | 208.2 | 1.46 (1.13-1.79) |
| High-income North America | 11,635 (4,242-18,079) | 7.8 (2.9-12.2) |  | 16,919 (6,345-28,778) | 10.1 (3.8-17.3) |  | 45.4 | 0.97 (0.74-1.20) |
| Oceania | 18 (4-34) | 0.6 (0.1-1.1) |  | 49 (11-96) | 0.7 (0.2-1.4) |  | 172.2 | 0.72 (0.69-0.75) |
| Central Sub-Saharan Africa | 209 (25-416) | 0.9 (0.1-1.7) |  | 522 (65-1,048) | 0.8 (0.1-1.7) |  | 149.8 | −0.04 (−0.33-0.24) |
| Eastern Sub-Saharan Africa | 715 (94-1,348) | 0.9 (0.1-1.6) |  | 2,081 (274-3,895) | 1.0 (0.1-2.0) |  | 191.0 | 0.71 (0.54-0.89) |
| Southern Sub-Saharan Africa | 307 (44-554) | 1.2 (0.2-2.1) |  | 636 (83-1,158) | 1.5 (0.2-2.7) |  | 107.2 | 0.83 (0.59-1.07) |
| Western Sub-Saharan Africa | 874 (119-1,577) | 1 (0.1-1.9) |  | 2945 (503-5,229) | 1.4 (0.2-2.4) |  | 237.0 | 1.22 (1.13-1.30) |

**Supplementary Table S8. Early-onset colorectal cancer-related DALYs burden attributable to individual dietary risk factors in 2019 at national level, both sexes**

| Location | A diet low in milk | | A diet low in whole grains | | A diet low in calcium | | A diet low in fiber | | A high in red meat | | A high in processed meat | |  |
| --- | --- | --- | --- | --- | --- | --- | --- | --- | --- | --- | --- | --- | --- |
|  | No. (95% UI) | ASR per 10^5^  No. (95% UI) | No. (95% UI) | ASR per 10^5^  No. (95% UI) | No. (95% UI) | ASR per 105  No. (95% UI) | No. (95% UI) | ASR per 10^5^  No. (95% UI) | No. (95% UI) | ASR per 10^5^  No. (95% UI) | No. (95% UI) | ASR per 10^5^  No. (95% UI) | |
| Afghanistan | 2333  (1060-3949) | 12.8 (5.8-21.7) | 2911  (1374-4855) | 16 (7.5-26.7) | 3080  (1591-4897) | 16.9 (8.7-26.9) | 573 (207-1194) | 3.1 (1.1-6.6) | 365 (24-1134) | 2 (0.1-6.2) | 127 (22-294) | 0.7 (0.1-1.6) | |
| Albania | 11 (2-38) | 0.8 (0.2-2.9) | 242 (117-352) | 18.7 (9-27.2) | 27 (13-49) | 2.1 (1-3.8) | 15 (5-37) | 1.2 (0.4-2.9) | 76 (18-154) | 5.9 (1.4-11.9) | 89 (31-165) | 6.9 (2.4-12.7) | |
| Algeria | 2045  (1002-3334) | 9.1 (4.5-14.9) | 2736  (1216-3890) | 12.2 (5.4-17.3) | 1334  (696-2260) | 6 (3.1-10.1) | 119 (40-285) | 0.5 (0.2-1.3) | 341 (27-1008) | 1.5 (0.1-4.5) | 168 (27-351) | 0.7 (0.1-1.6) | |
| American Samoa | 7 (4-11) | 25.6 (15.3-38.8) | 6 (2-10) | 22.4 (8.2-35.1) | 7 (5-10) | 25.8 (16.9-37.4) | 0 (0-0) | 0.4 (0.2-0.8) | 1 (0-4) | 5.2 (0.6-13.6) | 0 (0-1) | 1.6 (0.3-3.3) | |
| Andorra | 7 (3-12) | 17.1 (6.3-30.1) | 11 (4-17) | 27.3 (10.1-42.1) | 2 (1-4) | 4.3 (1.7-8.7) | 1 (0-3) | 2.6 (0.7-6.4) | 7 (2-12) | 16.1 (5.7-29.3) | 4 (1-8) | 10.9 (3.6-18.8) | |
| Angola | 1528  (894-2323) | 11.3 (6.6-17.2) | 1253  (460-1925) | 9.3 (3.4-14.2) | 1892  (1206-2704) | 14 (8.9-20) | 72 (24-182) | 0.5 (0.2-1.3) | 210 (15-634) | 1.6 (0.1-4.7) | 156 (18-328) | 1.2 (0.1-2.4) | |
| Antigua and Barbuda | 6 (4-10) | 13.4 (7.4-20.4) | 8 (3-11) | 16.5 (7.1-22.5) | 5 (3-7) | 9.5 (5.4-15.1) | 2 (1-4) | 5 (2.2-7.8) | 1 (0-3) | 2.4 (0.2-6.7) | 1 (0-1) | 1.1 (0.2-2.3) | |
| Argentina | 5650  (3108-8176) | 24.4 (13.4-35.3) | 6278  (2345-8266) | 27.1 (10.1-35.6) | 3441  (1865-5650) | 14.8 (8-24.4) | 1311  (495-2373) | 5.7 (2.1-10.2) | 4787  (2376-7099) | 20.6 (10.2-30.6) | 1767  (348-2869) | 7.6 (1.5-12.4) | |
| Armenia | 158 (56-269) | 10.7 (3.8-18.2) | 348 (167-459) | 23.5 (11.3-31.1) | 106 (52-188) | 7.2 (3.5-12.7) | 30 (9-69) | 2 (0.6-4.7) | 73 (10-165) | 4.9 (0.7-11.1) | 54 (7-96) | 3.6 (0.5-6.5) | |
| Australia | 672 (179-1385) | 5.8 (1.6-12) | 2148  (787-2906) | 18.6 (6.8-25.2) | 764 (344-1363) | 6.6 (3-11.8) | 395 (137-740) | 3.4 (1.2-6.4) | 1798  (852-2648) | 15.6 (7.4-23) | 885 (289-1355) | 7.7 (2.5-11.7) | |
| Austria | 314 (132-507) | 7.8 (3.3-12.6) | 451 (165-616) | 11.2 (4.1-15.3) | 143 (59-255) | 3.6 (1.5-6.3) | 43 (14-101) | 1.1 (0.3-2.5) | 287 (102-474) | 7.1 (2.5-11.8) | 186 (58-283) | 4.6 (1.4-7) | |
| Azerbaijan | 1159  (614-1784) | 20.8 (11-32.1) | 1623  (783-2228) | 29.2 (14.1-40) | 767 (412-1266) | 13.8 (7.4-22.8) | 95 (30-225) | 1.7 (0.5-4.1) | 254 (21-706) | 4.6 (0.4-12.7) | 361 (78-620) | 6.5 (1.4-11.2) | |
| Bahamas | 74 (46-110) | 35.9 (22.3-53.5) | 70 (27-100) | 34.2 (13.4-48.6) | 42 (23-68) | 20.7 (11.4-33.4) | 18 (7-30) | 8.6 (3.2-14.6) | 18 (3-43) | 9 (1.3-21.2) | 5 (1-11) | 2.6 (0.4-5.4) | |
| Bahrain | 129 (73-196) | 14 (7.9-21.2) | 140 (53-202) | 15.1 (5.7-21.9) | 72 (37-122) | 7.8 (4-13.2) | 4 (2-10) | 0.4 (0.2-1.1) | 23 (2-63) | 2.5 (0.2-6.8) | 10 (2-20) | 1.1 (0.2-2.2) | |
| Bangladesh | 5194  (2833-8611) | 6 (3.3-10) | 3346  (1160-5963) | 3.9 (1.3-6.9) | 7376  (4395-11499) | 8.6 (5.1-13.3) | 2359  (1049-4034) | 2.7 (1.2-4.7) | 274 (47-756) | 0.3 (0.1-0.9) | 1019  (140-2169) | 1.2 (0.2-2.5) | |
| Barbados | 45 (28-65) | 32 (20.1-46.2) | 39 (14-55) | 27.9 (10.2-39.6) | 27 (15-42) | 19.1 (10.9-30) | 8 (3-15) | 5.4 (1.8-10.6) | 8 (1-22) | 5.5 (0.5-15.4) | 4 (1-8) | 2.7 (0.4-5.5) | |
| Belarus | 617 (230-1075) | 14.2 (5.3-24.7) | 1078  (447-1569) | 24.7 (10.3-36) | 275 (117-514) | 6.3 (2.7-11.8) | 30 (13-74) | 0.7 (0.3-1.7) | 561 (177-1003) | 12.9 (4.1-23) | 348 (118-586) | 8 (2.7-13.4) | |
| Belgium | 527 (233-828) | 10.5 (4.7-16.5) | 735 (275-983) | 14.7 (5.5-19.6) | 173 (78-308) | 3.5 (1.6-6.1) | 104 (35-219) | 2.1 (0.7-4.4) | 456 (161-747) | 9.1 (3.2-14.9) | 307 (111-492) | 6.1 (2.2-9.8) | |
| Belize | 35 (23-48) | 15.6 (10.1-21.6) | 29 (11-41) | 13.1 (4.8-18.1) | 24 (15-36) | 10.7 (6.7-15.8) | 3 (1-6) | 1.1 (0.3-2.7) | 4 (0-12) | 1.9 (0.2-5.3) | 3 (0-5) | 1.3 (0.2-2.4) | |
| Benin | 433 (245-683) | 7.4 (4.2-11.7) | 326 (117-524) | 5.6 (2-9) | 517 (340-755) | 8.9 (5.8-13) | 6 (4-12) | 0.1 (0.1-0.2) | 36 (4-98) | 0.6 (0.1-1.7) | 74 (9-152) | 1.3 (0.2-2.6) | |
| Bermuda | 7 (4-10) | 24.7 (15.5-35.8) | 6 (2-9) | 22.9 (8.5-33.3) | 4 (2-6) | 14.1 (7.7-22.9) | 1 (1-3) | 5.2 (2-9.5) | 3 (1-6) | 10.6 (2.8-20.3) | 1 (0-1) | 1.9 (0.3-3.8) | |
| Bhutan | 31 (14-54) | 7.1 (3.1-12.3) | 25 (9-45) | 5.8 (2-10.2) | 35 (16-58) | 8.1 (3.8-13.3) | 6 (2-13) | 1.4 (0.4-3) | 3 (0-9) | 0.7 (0.1-2) | 7 (1-16) | 1.7 (0.3-3.6) | |
| Bolivia | 782 (424-1206) | 12.8 (6.9-19.7) | 654 (238-1029) | 10.7 (3.9-16.8) | 988 (605-1433) | 16.2 (9.9-23.5) | 115 (33-238) | 1.9 (0.5-3.9) | 201 (29-473) | 3.3 (0.5-7.7) | 36 (9-74) | 0.6 (0.1-1.2) | |
| Bosnia and Herzegovina | 299 (122-494) | 19.7 (8-32.5) | 404 (151-595) | 26.6 (10-39.2) | 165 (73-298) | 10.9 (4.8-19.6) | 8 (5-17) | 0.6 (0.3-1.1) | 74 (6-212) | 4.9 (0.4-13.9) | 138 (38-245) | 9.1 (2.5-16.1) | |
| Botswana | 252 (125-443) | 19 (9.4-33.5) | 228 (80-382) | 17.2 (6-28.9) | 226 (117-382) | 17.1 (8.8-28.9) | 28 (9-68) | 2.1 (0.6-5.1) | 52 (4-148) | 4 (0.3-11.2) | 22 (3-50) | 1.7 (0.2-3.8) | |
| Brazil | 17815  (9177-26567) | 15.4 (7.9-23) | 19167  (7152-25656) | 16.6 (6.2-22.2) | 13052  (7616-20208) | 11.3 (6.6-17.5) | 2921  (1088-5780) | 2.5 (0.9-5) | 14075  (6186-21586) | 12.2 (5.4-18.7) | 3204  (392-5501) | 2.8 (0.3-4.8) | |
| Brunei Darussalam | 95 (60-135) | 35.7 (22.6-50.6) | 81 (29-113) | 30.4 (11-42.6) | 60 (35-93) | 22.5 (13.1-35) | 22 (8-40) | 8.4 (3.2-14.9) | 15 (1-42) | 5.7 (0.4-15.6) | 19 (3-34) | 7 (1-12.8) | |
| Bulgaria | 1108  (579-1782) | 36.6 (19.1-58.8) | 1228  (455-1799) | 40.5 (15-59.4) | 590 (282-1056) | 19.5 (9.3-34.8) | 218 (75-438) | 7.2 (2.5-14.4) | 682 (222-1220) | 22.5 (7.3-40.3) | 446 (123-743) | 14.7 (4.1-24.5) | |
| Burkina Faso | 750 (433-1113) | 7.3 (4.2-10.9) | 640 (241-951) | 6.2 (2.4-9.3) | 926 (606-1309) | 9 (5.9-12.8) | 16 (8-38) | 0.2 (0.1-0.4) | 109 (8-327) | 1.1 (0.1-3.2) | 130 (16-255) | 1.3 (0.2-2.5) | |
| Burundi | 511 (297-817) | 9.3 (5.4-14.9) | 446 (154-721) | 8.1 (2.8-13.1) | 642 (412-971) | 11.7 (7.5-17.7) | 13 (5-31) | 0.2 (0.1-0.6) | 24 (5-60) | 0.4 (0.1-1.1) | 47 (6-104) | 0.9 (0.1-1.9) | |
| Cabo Verde | 30 (17-46) | 9.7 (5.6-15.1) | 24 (9-38) | 7.9 (2.9-12.4) | 21 (13-34) | 6.9 (4.1-11.2) | 2 (1-5) | 0.7 (0.2-1.7) | 4 (0-12) | 1.4 (0.1-4) | 5 (1-10) | 1.7 (0.2-3.4) | |
| Cambodia | 1968  (1239-2961) | 22.4 (14.1-33.7) | 1153  (426-1883) | 13.1 (4.9-21.4) | 2650  (1864-3683) | 30.2 (21.2-41.9) | 834 (422-1299) | 9.5 (4.8-14.8) | 246 (20-730) | 2.8 (0.2-8.3) | 73 (23-140) | 0.8 (0.3-1.6) | |
| Cameroon | 1578  (887-2489) | 11 (6.2-17.3) | 1214  (415-1957) | 8.4 (2.9-13.6) | 1869  (1172-2804) | 13 (8.2-19.5) | 28 (14-59) | 0.2 (0.1-0.4) | 193 (15-577) | 1.3 (0.1-4) | 277 (36-572) | 1.9 (0.2-4) | |
| Canada | 2010  (848-3152) | 12.4 (5.2-19.5) | 2741  (1018-3701) | 16.9 (6.3-22.9) | 1237  (607-2173) | 7.7 (3.8-13.4) | 270 (86-632) | 1.7 (0.5-3.9) | 1393  (384-2552) | 8.6 (2.4-15.8) | 982 (284-1543) | 6.1 (1.8-9.5) | |
| Central African Republic | 233 (130-379) | 9.1 (5.1-14.8) | 195 (69-310) | 7.6 (2.7-12.1) | 289 (180-439) | 11.3 (7-17.1) | 18 (6-44) | 0.7 (0.2-1.7) | 59 (9-147) | 2.3 (0.3-5.7) | 22 (3-48) | 0.8 (0.1-1.9) | |
| Chad | 445 (263-667) | 6.5 (3.8-9.8) | 510 (253-745) | 7.5 (3.7-10.9) | 550 (367-782) | 8 (5.4-11.4) | 19 (7-50) | 0.3 (0.1-0.7) | 64 (5-190) | 0.9 (0.1-2.8) | 70 (8-138) | 1 (0.1-2) | |
| Chile | 1578  (1041-2165) | 17.2 (11.3-23.5) | 1482  (547-1960) | 16.1 (5.9-21.3) | 1314  (915-1833) | 14.3 (10-19.9) | 215 (74-447) | 2.3 (0.8-4.9) | 713 (210-1230) | 7.8 (2.3-13.4) | 591 (216-929) | 6.4 (2.3-10.1) | |
| China | 241940  (158262-332973) | 33.6 (22-46.2) | 205978  (77548-288718) | 28.6 (10.8-40.1) | 196595 (131935-275002) | 27.3 (18.3-38.2) | 22233  (7666-46938) | 3.1 (1.1-6.5) | 100845  (32362-176499) | 14 (4.5-24.5) | 20156  (2966-37531) | 2.8 (0.4-5.2) | |
| Colombia | 3277  (1620-5327) | 13.2 (6.5-21.4) | 3522  (1302-5251) | 14.1 (5.2-21.1) | 3166  (1927-4841) | 12.7 (7.7-19.4) | 618 (207-1290) | 2.5 (0.8-5.2) | 914 (105-2338) | 3.7 (0.4-9.4) | 442 (51-875) | 1.8 (0.2-3.5) | |
| Comoros | 47 (24-77) | 12.6 (6.3-20.5) | 38 (13-62) | 10.3 (3.4-16.5) | 65 (36-97) | 17.3 (9.5-26) | 4 (1-10) | 1.2 (0.3-2.7) | 4 (0-12) | 1.1 (0.1-3.1) | 5 (1-11) | 1.4 (0.2-3) | |
| Republic of Congo | 647 (397-986) | 24.2 (14.8-36.9) | 340 (121-547) | 12.7 (4.5-20.5) | 578 (343-879) | 21.6 (12.8-32.9) | 78 (27-155) | 2.9 (1-5.8) | 57 (4-179) | 2.1 (0.1-6.7) | 44 (5-96) | 1.6 (0.2-3.6) | |
| Cook Islands | 1 (1-2) | 13.5 (6.8-21) | 1 (0-1) | 11.7 (4.2-18.1) | 1 (0-1) | 11.4 (6.1-17.6) | 0 (0-0) | 0.2 (0.1-0.2) | 0 (0-1) | 3.1 (0.4-7.9) | 0 (0-0) | 0.9 (0.1-1.9) | |
| Costa Rica | 415 (190-682) | 16.6 (7.6-27.3) | 546 (212-796) | 21.9 (8.5-31.9) | 295 (150-504) | 11.8 (6-20.2) | 128 (47-242) | 5.1 (1.9-9.7) | 109 (10-299) | 4.4 (0.4-12) | 61 (7-124) | 2.4 (0.3-5) | |
| Côte d'Ivoire | 1471  (866-2250) | 11.3 (6.6-17.2) | 1095  (401-1685) | 8.4 (3.1-12.9) | 1634 (1052-2362) | 12.5 (8.1-18.1) | 17 (11-27) | 0.1 (0.1-0.2) | 174 (12-533) | 1.3 (0.1-4.1) | 245 (33-477) | 1.9 (0.3-3.7) | |
| Croatia | 310 (125-509) | 16.6 (6.7-27.3) | 464 (171-679) | 24.9 (9.2-36.5) | 177 (79-319) | 9.5 (4.2-17.1) | 99 (34-191) | 5.3 (1.8-10.3) | 126 (18-303) | 6.8 (1-16.2) | 40 (6-82) | 2.1 (0.3-4.4) | |
| Cuba | 1146  (690-1709) | 21.6 (13-32.2) | 1005  (368-1436) | 18.9 (6.9-27) | 697 (393-1135) | 13.1 (7.4-21.4) | 24 (13-54) | 0.5 (0.2-1) | 231 (23-602) | 4.3 (0.4-11.3) | 116 (15-233) | 2.2 (0.3-4.4) | |
| Cyprus | 62 (28-96) | 8.9 (4.1-13.9) | 88 (35-120) | 12.8 (5-17.4) | 40 (19-69) | 5.8 (2.8-10.1) | 20 (8-35) | 2.9 (1.1-5) | 27 (6-55) | 3.9 (0.8-8) | 26 (6-43) | 3.8 (0.9-6.3) | |
| Czech Republic | 942 (484-1487) | 19.6 (10.1-31) | 938 (346-1371) | 19.5 (7.2-28.6) | 363 (160-672) | 7.6 (3.3-14) | 182 (63-377) | 3.8 (1.3-7.8) | 455 (110-871) | 9.5 (2.3-18.1) | 339 (84-572) | 7.1 (1.7-11.9) | |
|  |  |  |  |  |  |  |  |  |  |  |  |  | |
| North Korea | 4303  (2299-7277) | 30.9 (16.5-52.3) | 3271  (1108-5526) | 23.5 (8-39.7) | 5531  (3253-8901) | 39.7 (23.4-64) | 671 (223-1416) | 4.8 (1.6-10.2) | 469 (38-1453) | 3.4 (0.3-10.4) | 261 (39-598) | 1.9 (0.3-4.3) | |
| Democratic Republic of the Congo | 4539  (2750-7133) | 10.9 (6.6-17.1) | 2259  (766-3747) | 5.4 (1.8-9) | 4081  (2454-6324) | 9.8 (5.9-15.2) | 377 (119-804) | 0.9 (0.3-1.9) | 153 (28-399) | 0.4 (0.1-1) | 270 (32-584) | 0.6 (0.1-1.4) | |
| Denmark | 257 (99-415) | 10 (3.9-16.2) | 429 (159-568) | 16.7 (6.2-22.2) | 74 (33-128) | 2.9 (1.3-5) | 48 (16-110) | 1.9 (0.6-4.3) | 246 (79-406) | 9.6 (3.1-15.8) | 175 (63-274) | 6.8 (2.5-10.7) | |
| Djibouti | 101 (51-176) | 15.8 (7.9-27.4) | 94 (34-159) | 14.6 (5.3-24.8) | 113 (64-181) | 17.6 (9.9-28.1) | 21 (7-43) | 3.3 (1-6.7) | 16 (1-52) | 2.6 (0.2-8.1) | 11 (1-24) | 1.7 (0.2-3.7) | |
| Dominica | 6 (3-9) | 17 (9.4-26.8) | 8 (4-11) | 22.6 (11.1-32.1) | 2 (1-4) | 6.4 (2.5-12.5) | 0 (0-0) | 0.4 (0.2-1) | 1 (0-3) | 3.3 (0.3-9.4) | 1 (0-1) | 1.7 (0.2-3.4) | |
| Dominican Republic | 1129  (619-1785) | 19.6 (10.7-30.9) | 1003  (373-1541) | 17.4 (6.5-26.7) | 1186  (737-1753) | 20.6 (12.8-30.4) | 191 (64-405) | 3.3 (1.1-7) | 187 (14-550) | 3.2 (0.2-9.5) | 99 (14-210) | 1.7 (0.2-3.6) | |
| Ecuador | 1234  (648-1963) | 13.4 (7.1-21.4) | 1269  (457-1876) | 13.8 (5-20.4) | 1328  (868-1893) | 14.4 (9.4-20.6) | 444 (175-718) | 4.8 (1.9-7.8) | 406 (66-892) | 4.4 (0.7-9.7) | 180 (19-357) | 2 (0.2-3.9) | |
| Egypt | 6769  (3931-10356) | 13.2 (7.6-20.1) | 5697  (2039-8889) | 11.1 (4-17.3) | 5145  (3051-8024) | 10 (5.9-15.6) | 109 (61-221) | 0.2 (0.1-0.4) | 979 (73-2961) | 1.9 (0.1-5.8) | 623 (72-1333) | 1.2 (0.1-2.6) | |
| El Salvador | 480 (255-786) | 14.6 (7.8-23.9) | 463 (171-703) | 14.1 (5.2-21.4) | 437 (258-665) | 13.3 (7.9-20.3) | 21 (8-54) | 0.6 (0.2-1.6) | 63 (6-180) | 1.9 (0.2-5.5) | 51 (6-108) | 1.6 (0.2-3.3) | |
| Equatorial Guinea | 107 (53-186) | 14.3 (7.1-24.8) | 70 (22-128) | 9.4 (3-17.2) | 94 (48-162) | 12.5 (6.4-21.7) | 3 (1-7) | 0.4 (0.1-1) | 16 (1-48) | 2.1 (0.2-6.4) | 12 (1-27) | 1.6 (0.2-3.6) | |
| Eritrea | 489 (278-792) | 14.2 (8-22.9) | 394 (143-642) | 11.4 (4.2-18.6) | 639 (420-940) | 18.5 (12.1-27.2) | 24 (8-67) | 0.7 (0.2-1.9) | 53 (5-147) | 1.5 (0.1-4.3) | 50 (6-108) | 1.4 (0.2-3.1) | |
| Estonia | 84 (37-138) | 14.4 (6.3-23.7) | 111 (41-162) | 19 (7-27.8) | 21 (9-38) | 3.7 (1.6-6.5) | 5 (2-14) | 0.9 (0.3-2.5) | 35 (6-79) | 6 (1-13.6) | 61 (19-119) | 10.5 (3.3-20.4) | |
| Eswatini | 99 (48-173) | 16.4 (7.9-28.5) | 75 (26-131) | 12.3 (4.3-21.6) | 125 (69-200) | 20.6 (11.4-32.9) | 9 (3-22) | 1.6 (0.4-3.7) | 20 (2-57) | 3.3 (0.3-9.4) | 8 (1-19) | 1.4 (0.2-3.2) | |
| Ethiopia | 4344  (2459-6850) | 8.3 (4.7-13) | 4107  (1553-6314) | 7.8 (3-12) | 5970  (4004-8626) | 11.4 (7.6-16.4) | 115 (51-264) | 0.2 (0.1-0.5) | 491 (46-1391) | 0.9 (0.1-2.6) | 442 (58-925) | 0.8 (0.1-1.8) | |
| Fiji | 83 (50-124) | 17.8 (10.7-26.6) | 74 (27-110) | 15.9 (5.7-23.5) | 89 (60-129) | 19 (12.9-27.6) | 3 (1-8) | 0.7 (0.3-1.8) | 19 (2-48) | 4 (0.5-10.3) | 5 (1-10) | 1 (0.2-2.2) | |
| Finland | 41 (7-108) | 1.7 (0.3-4.6) | 269 (100-362) | 11.5 (4.3-15.5) | 37 (17-65) | 1.6 (0.7-2.8) | 26 (9-61) | 1.1 (0.4-2.6) | 137 (37-242) | 5.9 (1.6-10.4) | 103 (32-160) | 4.4 (1.4-6.8) | |
| France | 3491  (1587-5400) | 12.3 (5.6-19) | 5090  (1982-6683) | 17.9 (7-23.5) | 1233  (536-2221) | 4.3 (1.9-7.8) | 828 (288-1630) | 2.9 (1-5.7) | 3144 (1312-4829) | 11.1 (4.6-17) | 1932  (681-3086) | 6.8 (2.4-10.9) | |
| Gabon | 208 (125-327) | 22.5 (13.5-35.4) | 120 (45-194) | 13 (4.8-21) | 192 (115-298) | 20.8 (12.5-32.3) | 9 (3-24) | 1 (0.3-2.6) | 54 (10-117) | 5.8 (1-12.7) | 19 (2-41) | 2.1 (0.2-4.5) | |
| Gambia | 69 (39-109) | 6.2 (3.5-9.8) | 53 (18-84) | 4.7 (1.6-7.5) | 86 (54-129) | 7.7 (4.8-11.6) | 5 (2-12) | 0.5 (0.1-1.1) | 5 (1-15) | 0.5 (0.1-1.3) | 12 (2-23) | 1 (0.1-2.1) | |
| Georgia | 329 (143-530) | 19.9 (8.6-32) | 582 (285-791) | 35.2 (17.3-47.8) | 237 (116-416) | 14.3 (7-25.2) | 49 (16-110) | 2.9 (0.9-6.6) | 80 (6-226) | 4.9 (0.4-13.7) | 123 (24-218) | 7.5 (1.4-13.2) | |
| Germany | 4135  (1728-6491) | 11.5 (4.8-18.1) | 5930  (2227-7907) | 16.5 (6.2-22) | 1432  (636-2501) | 4 (1.8-7) | 845 (279-1826) | 2.4 (0.8-5.1) | 3667  (1243-5969) | 10.2 (3.5-16.6) | 2799  (1091-4662) | 7.8 (3-13) | |
| Ghana | 1991  (1229-2995) | 12 (7.4-18) | 1524  (539-2297) | 9.2 (3.2-13.8) | 1899  (1239-2769) | 11.4 (7.5-16.7) | 20 (14-28) | 0.1 (0.1-0.2) | 179 (17-508) | 1.1 (0.1-3.1) | 361 (57-687) | 2.2 (0.3-4.1) | |
| Greece | 625 (316-938) | 14 (7.1-21) | 930 (445-1180) | 20.8 (10-26.4) | 173 (76-303) | 3.9 (1.7-6.8) | 63 (20-152) | 1.4 (0.5-3.4) | 459 (169-733) | 10.3 (3.8-16.4) | 254 (67-395) | 5.7 (1.5-8.8) | |
| Greenland | 6 (3-11) | 24 (9.7-40) | 9 (3-13) | 33.2 (12.2-49.5) | 2 (1-4) | 7.6 (3.1-14.2) | 1 (0-2) | 3.5 (1.1-8.2) | 5 (2-10) | 20.6 (7.4-37) | 4 (1-7) | 15.3 (5.6-26.9) | |
| Grenada | 13 (9-19) | 24.4 (16.6-34.6) | 12 (5-17) | 22.8 (9.2-31.9) | 8 (5-13) | 15.5 (9.1-23.5) | 1 (0-3) | 2.5 (0.7-5.9) | 2 (0-4) | 2.8 (0.3-7.8) | 1 (0-2) | 1.7 (0.3-3.5) | |
| Guam | 25 (16-34) | 30.4 (19.4-42.5) | 22 (8-31) | 26.7 (9.7-37.8) | 17 (11-26) | 21.2 (13-31.8) | 0 (0-0) | 0.3 (0.3-0.4) | 7 (1-15) | 8.3 (1.3-18.1) | 2 (0-3) | 2.1 (0.4-4.2) | |
| Guatemala | 1323  (801-1971) | 13.8 (8.4-20.6) | 1121  (424-1642) | 11.7 (4.4-17.2) | 1504  (1049-2051) | 15.7 (11-21.4) | 58 (21-134) | 0.6 (0.2-1.4) | 143 (15-402) | 1.5 (0.2-4.2) | 119 (16-238) | 1.2 (0.2-2.5) | |
| Guinea | 418 (246-639) | 7.3 (4.3-11.2) | 319 (114-500) | 5.6 (2-8.7) | 546 (354-780) | 9.5 (6.2-13.6) | 30 (9-74) | 0.5 (0.2-1.3) | 42 (4-116) | 0.7 (0.1-2) | 72 (10-140) | 1.3 (0.2-2.4) | |
| Guinea-Bissau | 100 (60-152) | 10.6 (6.3-16.1) | 75 (27-116) | 7.9 (2.8-12.2) | 143 (95-207) | 15.1 (10.1-21.9) | 14 (5-29) | 1.5 (0.5-3.1) | 14 (1-41) | 1.5 (0.1-4.4) | 16 (2-31) | 1.6 (0.2-3.3) | |
| Guyana | 96 (55-150) | 23.3 (13.2-36.1) | 87 (31-127) | 21 (7.5-30.7) | 82 (50-123) | 19.7 (12-29.6) | 16 (5-33) | 3.9 (1.3-7.9) | 8 (1-22) | 2 (0.3-5.3) | 9 (1-18) | 2.2 (0.3-4.4) | |
| Haiti | 947 (533-1502) | 14.5 (8.1-22.9) | 796 (302-1314) | 12.1 (4.6-20.1) | 1246  (798-1879) | 19 (12.2-28.7) | 127 (43-280) | 1.9 (0.7-4.3) | 100 (9-287) | 1.5 (0.1-4.4) | 61 (10-136) | 0.9 (0.2-2.1) | |
| Honduras | 352 (159-640) | 6.8 (3.1-12.4) | 331 (114-557) | 6.4 (2.2-10.8) | 360 (194-599) | 7 (3.8-11.6) | 26 (7-69) | 0.5 (0.1-1.3) | 52 (4-161) | 1 (0.1-3.1) | 38 (5-88) | 0.7 (0.1-1.7) | |
| Hungary | 1214  (576-1920) | 27.3 (13-43.2) | 1474  (543-2102) | 33.2 (12.2-47.3) | 790 (416-1351) | 17.8 (9.4-30.4) | 213 (68-468) | 4.8 (1.5-10.5) | 496 (106-1064) | 11.2 (2.4-24) | 518 (130-832) | 11.7 (2.9-18.7) | |
| Iceland | 10 (3-16) | 5.9 (2.1-10) | 17 (6-24) | 10.6 (3.9-14.4) | 2 (1-4) | 1.3 (0.6-2.3) | 4 (1-7) | 2.4 (0.9-4.4) | 12 (5-19) | 7.2 (2.9-11.4) | 7 (2-11) | 4.1 (1.5-6.7) | |
| India | 92959  (62907-124683) | 12.2 (8.3-16.4) | 64835  (24153-91108) | 8.5 (3.2-12) | 64943  (44308-89482) | 8.5 (5.8-11.8) | 9939 (3430-21494) | 1.3 (0.5-2.8) | 3736  (1238-7592) | 0.5 (0.2-1) | 6289  (4066-9733) | 0.8 (0.5-1.3) | |
| Indonesia | 37647  (23738-54578) | 26.4 (16.6-38.2) | 24675  (9498-38446) | 17.3 (6.7-26.9) | 49714  (34181-69893) | 34.8 (23.9-48.9) | 7821 (3120-13992) | 5.5 (2.2-9.8) | 2861  (402-7588) | 2 (0.3-5.3) | 1116  (584-1827) | 0.8 (0.4-1.3) | |
| Iran | 6796  (4196-9474) | 14.4 (8.9-20) | 8490  (4126-10623) | 18 (8.7-22.5) | 6764  (4946-8978) | 14.3 (10.5-19) | 353 (141-810) | 0.7 (0.3-1.7) | 958 (90-2539) | 2 (0.2-5.4) | 494 (96-909) | 1 (0.2-1.9) | |
| Iraq | 3037  (1817-4677) | 13.1 (7.9-20.2) | 3005  (1189-4690) | 13 (5.1-20.3) | 3655  (2510-5475) | 15.8 (10.9-23.7) | 334 (108-757) | 1.4 (0.5-3.3) | 238 (30-648) | 1 (0.1-2.8) | 168 (31-357) | 0.7 (0.1-1.5) | |
| Ireland | 131 (37-255) | 5.6 (1.6-11) | 308 (113-433) | 13.2 (4.9-18.6) | 44 (18-79) | 1.9 (0.8-3.4) | 30 (10-75) | 1.3 (0.4-3.2) | 292 (153-443) | 12.5 (6.5-19) | 137 (44-215) | 5.9 (1.9-9.2) | |
| Israel | 565 (274-839) | 12.9 (6.3-19.2) | 787 (352-1005) | 18 (8.1-23) | 163 (73-284) | 3.7 (1.7-6.5) | 29 (12-73) | 0.7 (0.3-1.7) | 196 (34-423) | 4.5 (0.8-9.7) | 209 (46-331) | 4.8 (1.1-7.6) | |
| Italy | 3617  (1772-5453) | 14.3 (7-21.5) | 6031  (2902-7520) | 23.8 (11.4-29.7) | 1462  (711-2512) | 5.8 (2.8-9.9) | 583 (215-1201) | 2.3 (0.8-4.7) | 2406  (781-4067) | 9.5 (3.1-16) | 1845  (660-2821) | 7.3 (2.6-11.1) | |
| Jamaica | 311 (172-490) | 20.3 (11.3-32) | 328 (121-491) | 21.4 (7.9-32.1) | 241 (138-378) | 15.7 (9-24.7) | 29 (9-69) | 1.9 (0.6-4.5) | 45 (4-131) | 2.9 (0.3-8.5) | 31 (4-64) | 2 (0.3-4.2) | |
| Japan | 11786  (7385-16200) | 22.8 (14.3-31.3) | 10532  (3928-13882) | 20.3 (7.6-26.8) | 9991  (6814-13743) | 19.3 (13.2-26.6) | 2023  (764-3692) | 3.9 (1.5-7.1) | 2214  (235-5589) | 4.3 (0.5-10.8) | 4420  (1484-6728) | 8.5 (2.9-13) | |
| Jordan | 918 (542-1342) | 14.5 (8.6-21.2) | 1131  (545-1520) | 17.9 (8.6-24) | 805 (521-1142) | 12.7 (8.2-18) | 115 (38-248) | 1.8 (0.6-3.9) | 136 (11-390) | 2.1 (0.2-6.2) | 64 (11-128) | 1 (0.2-2) | |
| Kazakhstan | 386 (89-863) | 4.2 (1-9.3) | 1995  (1019-2632) | 21.5 (11-28.3) | 304 (140-541) | 3.3 (1.5-5.8) | 171 (56-390) | 1.8 (0.6-4.2) | 933 (318-1506) | 10.1 (3.4-16.2) | 474 (109-777) | 5.1 (1.2-8.4) | |
| Kenya | 2044  (1088-3282) | 7.8 (4.2-12.6) | 1936  (735-2858) | 7.4 (2.8-11) | 1736  (1015-2666) | 6.7 (3.9-10.2) | 91 (38-202) | 0.4 (0.1-0.8) | 350 (33-975) | 1.3 (0.1-3.7) | 276 (40-534) | 1.1 (0.2-2) | |
| Kiribati | 14 (8-21) | 22.6 (13.6-34.1) | 12 (4-18) | 20.1 (6.9-30.1) | 19 (13-27) | 31.5 (21.3-44.9) | 1 (0-2) | 1.3 (0.4-3) | 2 (0-5) | 2.9 (0.2-8.4) | 1 (0-1) | 1.2 (0.2-2.4) | |
| Kuwait | 289 (178-419) | 9.9 (6.1-14.4) | 267 (98-369) | 9.2 (3.4-12.7) | 111 (54-201) | 3.8 (1.9-6.9) | 26 (8-59) | 0.9 (0.3-2) | 76 (11-172) | 2.6 (0.4-5.9) | 22 (4-43) | 0.7 (0.1-1.5) | |
| Kyrgyzstan | 97 (26-198) | 2.9 (0.8-5.9) | 427 (214-558) | 12.7 (6.4-16.6) | 120 (55-219) | 3.6 (1.6-6.5) | 32 (11-74) | 1 (0.3-2.2) | 108 (22-227) | 3.2 (0.7-6.8) | 93 (21-162) | 2.8 (0.6-4.8) | |
| Laos | 1030  (602-1546) | 26.4 (15.4-39.7) | 535 (206-886) | 13.7 (5.3-22.7) | 1176  (735-1706) | 30.2 (18.9-43.8) | 291 (133-476) | 7.5 (3.4-12.2) | 127 (9-385) | 3.3 (0.2-9.9) | 33 (10-67) | 0.9 (0.3-1.7) | |
| Latvia | 101 (40-172) | 12.3 (4.9-21) | 165 (59-235) | 20.1 (7.2-28.7) | 33 (13-60) | 4 (1.6-7.3) | 20 (6-44) | 2.4 (0.7-5.3) | 48 (7-107) | 5.8 (0.9-13) | 90 (29-181) | 11 (3.5-22.1) | |
| Lebanon | 591 (353-902) | 22.1 (13.2-33.8) | 685 (315-975) | 25.7 (11.8-36.5) | 255 (119-466) | 9.5 (4.4-17.5) | 20 (8-60) | 0.8 (0.3-2.2) | 111 (10-300) | 4.1 (0.4-11.2) | 39 (7-81) | 1.5 (0.2-3) | |
| Lesotho | 170 (93-278) | 14.8 (8.2-24.2) | 114 (40-192) | 10 (3.5-16.8) | 243 (145-357) | 21.2 (12.6-31.1) | 2 (1-4) | 0.2 (0.1-0.3) | 27 (2-80) | 2.4 (0.2-7) | 15 (2-33) | 1.3 (0.2-2.9) | |
| Liberia | 215 (116-357) | 8.7 (4.7-14.5) | 144 (50-237) | 5.8 (2-9.6) | 257 (147-410) | 10.4 (5.9-16.6) | 43 (16-79) | 1.7 (0.7-3.2) | 14 (2-41) | 0.6 (0.1-1.7) | 29 (4-62) | 1.2 (0.1-2.5) | |
| Libya | 954 (541-1510) | 23.2 (13.2-36.8) | 1076  (467-1624) | 26.2 (11.4-39.5) | 712 (410-1164) | 17.3 (10-28.3) | 88 (28-207) | 2.1 (0.7-5) | 139 (10-400) | 3.4 (0.2-9.7) | 64 (11-139) | 1.5 (0.3-3.4) | |
| Lithuania | 184 (86-294) | 15.2 (7.1-24.3) | 234 (87-330) | 19.3 (7.2-27.3) | 59 (24-108) | 4.9 (2-8.9) | 13 (4-32) | 1 (0.3-2.7) | 106 (25-201) | 8.8 (2-16.6) | 138 (41-283) | 11.4 (3.3-23.4) | |
| Luxembourg | 21 (7-37) | 6.9 (2.3-12.1) | 42 (16-56) | 13.5 (5.1-18.4) | 6 (3-10) | 1.9 (0.9-3.2) | 6 (2-12) | 1.9 (0.6-4.1) | 30 (12-47) | 9.8 (3.9-15.4) | 21 (8-37) | 6.8 (2.5-12.2) | |
| Madagascar | 1286  (728-1983) | 9.7 (5.5-15) | 965 (349-1598) | 7.3 (2.6-12.1) | 1732  (1145-2465) | 13.1 (8.7-18.7) | 192 (63-410) | 1.5 (0.5-3.1) | 166 (13-485) | 1.3 (0.1-3.7) | 138 (15-299) | 1 (0.1-2.3) | |
| Malawi | 571 (327-907) | 6.3 (3.6-10.1) | 385 (131-628) | 4.3 (1.5-7) | 790 (487-1171) | 8.8 (5.4-13) | 8 (4-13) | 0.1 (0-0.1) | 49 (5-144) | 0.5 (0.1-1.6) | 60 (7-129) | 0.7 (0.1-1.4) | |
| Malaysia | 4178  (2598-6061) | 23.9 (14.9-34.7) | 3271 (1186-4938) | 18.7 (6.8-28.3) | 4528  (3052-6361) | 25.9 (17.5-36.4) | 897 (338-1631) | 5.1 (1.9-9.3) | 485 (42-1385) | 2.8 (0.2-7.9) | 405 (44-811) | 2.3 (0.3-4.6) | |
| Maldives | 46 (34-61) | 14.4 (10.7-19.2) | 26 (10-37) | 8.2 (3-11.7) | 25 (17-36) | 7.9 (5.3-11.4) | 7 (2-12) | 2.1 (0.8-3.6) | 2 (0-5) | 0.7 (0.1-1.6) | 1 (0-2) | 0.4 (0.1-0.6) | |
| Mali | 623 (318-1031) | 6.5 (3.3-10.7) | 609 (222-940) | 6.3 (2.3-9.7) | 625 (376-953) | 6.5 (3.9-9.9) | 19 (8-46) | 0.2 (0.1-0.5) | 137 (13-391) | 1.4 (0.1-4.1) | 139 (18-276) | 1.4 (0.2-2.9) | |
| Malta | 24 (12-36) | 12.2 (6.2-18.4) | 34 (17-46) | 17.5 (8.6-23.1) | 8 (3-15) | 4.2 (1.8-7.7) | 2 (1-6) | 1.2 (0.4-2.9) | 11 (2-21) | 5.5 (1.2-10.9) | 13 (5-22) | 6.4 (2.3-11.1) | |
| Marshall Islands | 8 (4-12) | 25.7 (14.1-39.4) | 7 (2-11) | 22.5 (8.2-34.7) | 10 (6-14) | 31.9 (19.9-46) | 0 (0-0) | 0.7 (0.3-1.6) | 1 (0-3) | 4.1 (0.3-11.2) | 0 (0-1) | 1.4 (0.3-2.9) | |
| Mauritania | 100 (46-173) | 5.2 (2.4-8.9) | 120 (43-198) | 6.2 (2.2-10.2) | 66 (31-123) | 3.4 (1.6-6.3) | 8 (2-19) | 0.4 (0.1-1) | 24 (2-67) | 1.2 (0.1-3.5) | 26 (4-49) | 1.3 (0.2-2.6) | |
| Mauritius | 195 (138-265) | 30 (21.2-40.8) | 121 (45-176) | 18.7 (6.8-27.1) | 82 (46-132) | 12.6 (7-20.4) | 23 (8-45) | 3.5 (1.2-6.9) | 19 (2-55) | 2.9 (0.2-8.5) | 7 (2-13) | 1 (0.3-2) | |
| Mexico | 9616  (5128-14203) | 14.5 (7.7-21.4) | 8277  (3112-11706) | 12.5 (4.7-17.7) | 8441  (5484-12220) | 12.7 (8.3-18.4) | 384 (172-856) | 0.6 (0.3-1.3) | 3567  (878-7173) | 5.4 (1.3-10.8) | 1420  (277-2555) | 2.1 (0.4-3.9) | |
| Micronesia (Federated States of) | 13 (4-22) | 24.3 (6.9-40.9) | 11 (3-19) | 21.2 (5-35.6) | 16 (5-26) | 30.5 (9-48.7) | 0 (0-1) | 0.6 (0.2-1.9) | 2 (0-6) | 3.9 (0.3-11.7) | 1 (0-1) | 1.2 (0.2-2.8) | |
| Monaco | 1 (0-2) | 7.1 (1.7-16.4) | 4 (1-6) | 29 (10.4-43.5) | 0 (0-0) | 0.4 (0.3-0.5) | 0 (0-0) | 0.7 (0.3-1.6) | 3 (1-5) | 21.4 (9.4-35.8) | 2 (1-3) | 12.4 (4.5-22.4) | |
| Mongolia | 179 (62-312) | 9.9 (3.4-17.2) | 386 (179-558) | 21.3 (9.9-30.9) | 196 (107-323) | 10.8 (5.9-17.8) | 105 (45-175) | 5.8 (2.5-9.7) | 277 (139-446) | 15.3 (7.7-24.7) | 80 (17-144) | 4.5 (1-8) | |
| Montenegro | 15 (4-31) | 5.1 (1.4-10.5) | 57 (22-80) | 19.3 (7.4-27) | 2 (1-3) | 0.6 (0.3-1.2) | 3 (1-8) | 1 (0.3-2.7) | 25 (7-46) | 8.3 (2.2-15.5) | 21 (7-33) | 6.9 (2.3-11.3) | |
| Morocco | 1981  (1024-3349) | 10.3 (5.3-17.5) | 2476  (1174-3954) | 12.9 (6.1-20.6) | 1913  (1151-3052) | 10 (6-15.9) | 30 (19-52) | 0.2 (0.1-0.3) | 318 (22-941) | 1.7 (0.1-4.9) | 135 (22-294) | 0.7 (0.1-1.5) | |
| Mozambique | 1200  (670-1919) | 9 (5-14.4) | 976 (345-1536) | 7.3 (2.6-11.5) | 1639  (993-2448) | 12.3 (7.5-18.4) | 62 (20-155) | 0.5 (0.1-1.2) | 98 (11-282) | 0.7 (0.1-2.1) | 122 (14-261) | 0.9 (0.1-2) | |
| Myanmar | 6860  (4066-10721) | 23.7 (14.1-37.1) | 4968  (1825-7993) | 17.2 (6.3-27.6) | 8806  (5656-12693) | 30.5 (19.6-43.9) | 2071  (839-3493) | 7.2 (2.9-12.1) | 920 (67-2713) | 3.2 (0.2-9.4) | 273 (87-534) | 0.9 (0.3-1.8) | |
| Namibia | 107 (54-182) | 8.6 (4.3-14.5) | 96 (34-161) | 7.7 (2.7-12.8) | 143 (85-221) | 11.4 (6.8-17.6) | 18 (6-39) | 1.4 (0.4-3.1) | 23 (2-64) | 1.8 (0.2-5.1) | 9 (1-20) | 0.7 (0.1-1.6) | |
| Nauru | 2 (1-3) | 32.3 (17.1-51.2) | 2 (1-2) | 28.3 (10.3-44.4) | 2 (1-3) | 35.7 (19-52) | 0 (0-0) | 0.5 (0.2-1) | 0 (0-1) | 5.9 (0.6-16.1) | 0 (0-0) | 1.9 (0.3-4) | |
| Nepal | 925 (475-1544) | 5.7 (2.9-9.5) | 756 (274-1263) | 4.7 (1.7-7.8) | 1129  (687-1706) | 7 (4.2-10.5) | 95 (27-218) | 0.6 (0.2-1.3) | 136 (10-407) | 0.8 (0.1-2.5) | 218 (34-426) | 1.3 (0.2-2.6) | |
| Netherlands | 1017  (430-1604) | 13.5 (5.7-21.4) | 1389  (524-1861) | 18.5 (7-24.8) | 163 (71-286) | 2.2 (0.9-3.8) | 193 (62-404) | 2.6 (0.8-5.4) | 862 (314-1390) | 11.5 (4.2-18.5) | 552 (176-833) | 7.3 (2.4-11.1) | |
| New Zealand | 222 (76-372) | 11.2 (3.8-18.8) | 423 (156-561) | 21.3 (7.8-28.3) | 298 (177-463) | 15 (8.9-23.3) | 62 (21-128) | 3.1 (1.1-6.4) | 328 (163-485) | 16.5 (8.2-24.4) | 166 (55-252) | 8.3 (2.8-12.7) | |
| Nicaragua | 335 (191-507) | 9.6 (5.4-14.5) | 291 (109-430) | 8.3 (3.1-12.3) | 380 (262-522) | 10.9 (7.5-14.9) | 34 (10-76) | 1 (0.3-2.2) | 37 (4-98) | 1 (0.1-2.8) | 32 (5-66) | 0.9 (0.1-1.9) | |
| Niger | 430 (226-713) | 4.5 (2.4-7.5) | 378 (136-612) | 4 (1.4-6.5) | 434 (267-680) | 4.6 (2.8-7.2) | 17 (6-40) | 0.2 (0.1-0.4) | 76 (6-223) | 0.8 (0.1-2.3) | 81 (11-171) | 0.9 (0.1-1.8) | |
| Nigeria | 6890  (4076-10519) | 6.8 (4-10.4) | 5411  (2038-8358) | 5.4 (2-8.3) | 7922  (5265-11586) | 7.9 (5.2-11.5) | 128 (67-272) | 0.1 (0.1-0.3) | 677 (65-1881) | 0.7 (0.1-1.9) | 1198  (190-2250) | 1.2 (0.2-2.2) | |
| Niue | 0 (0-0) | 24.1 (11.7-40.3) | 0 (0-0) | 21.1 (7.1-36.3) | 0 (0-0) | 25.6 (14-42.4) | 0 (0-0) | 0.3 (0.2-0.6) | 0 (0-0) | 4.6 (0.5-13.5) | 0 (0-0) | 1.4 (0.2-3.3) | |
| Macedonia | 226 (106-364) | 20.9 (9.7-33.6) | 318 (128-467) | 29.3 (11.8-43) | 130 (60-232) | 12 (5.6-21.4) | 24 (7-58) | 2.2 (0.7-5.4) | 53 (4-153) | 4.9 (0.4-14.1) | 89 (23-154) | 8.2 (2.1-14.2) | |
| Northern Mariana Islands | 7 (4-10) | 32.2 (19.6-50) | 6 (2-9) | 28.2 (10.4-42.7) | 6 (4-9) | 28.3 (17.2-43) | 0 (0-0) | 0.4 (0.3-0.5) | 1 (0-4) | 7.2 (0.8-18.5) | 0 (0-1) | 2 (0.4-4.4) | |
| Norway | 209 (74-353) | 8.4 (3-14.2) | 449 (176-579) | 18 (7.1-23.2) | 98 (48-168) | 4 (1.9-6.8) | 45 (17-98) | 1.8 (0.7-3.9) | 225 (77-375) | 9 (3.1-15.1) | 234 (78-447) | 9.4 (3.1-18) | |
| Oman | 201 (111-338) | 6.4 (3.5-10.8) | 205 (72-332) | 6.5 (2.3-10.6) | 114 (55-206) | 3.6 (1.8-6.6) | 12 (4-28) | 0.4 (0.1-0.9) | 50 (6-135) | 1.6 (0.2-4.3) | 14 (2-29) | 0.4 (0.1-0.9) | |
| Pakistan | 12364  (6395-19103) | 11.1 (5.7-17.1) | 16815  (7790-23437) | 15 (7-21) | 12687  (8193-18787) | 11.4 (7.3-16.8) | 3245  (1208-5808) | 2.9 (1.1-5.2) | 2068  (176-5979) | 1.9 (0.2-5.3) | 3026  (527-5533) | 2.7 (0.5-5) | |
| Palau | 2 (1-3) | 24.7 (14.4-37.6) | 2 (1-3) | 21.4 (7.6-32.1) | 2 (1-3) | 24.6 (15.4-35.7) | 0 (0-0) | 0.3 (0.2-0.5) | 0 (0-1) | 4.9 (0.4-13) | 0 (0-0) | 1.5 (0.3-3.4) | |
| Palestine | 483 (313-663) | 19 (12.3-26.1) | 534 (255-715) | 21.1 (10.1-28.2) | 627 (473-810) | 24.7 (18.7-31.9) | 112 (44-191) | 4.4 (1.8-7.5) | 51 (5-135) | 2 (0.2-5.3) | 24 (5-45) | 0.9 (0.2-1.8) | |
| Panama | 363 (226-536) | 17.3 (10.8-25.5) | 244 (91-367) | 11.6 (4.3-17.5) | 270 (162-403) | 12.9 (7.7-19.2) | 90 (36-161) | 4.3 (1.7-7.6) | 80 (12-189) | 3.8 (0.6-9) | 29 (4-60) | 1.4 (0.2-2.8) | |
| Papua New Guinea | 644 (376-998) | 12.7 (7.4-19.6) | 577 (207-890) | 11.4 (4.1-17.5) | 849 (557-1266) | 16.7 (11-24.9) | 11 (6-23) | 0.2 (0.1-0.5) | 93 (7-272) | 1.8 (0.1-5.3) | 30 (7-65) | 0.6 (0.1-1.3) | |
| Paraguay | 517 (269-844) | 13.9 (7.2-22.7) | 500 (187-751) | 13.5 (5-20.2) | 463 (271-715) | 12.5 (7.3-19.2) | 23 (8-60) | 0.6 (0.2-1.6) | 321 (103-558) | 8.6 (2.8-15) | 55 (7-115) | 1.5 (0.2-3.1) | |
| Peru | 2365  (1403-3582) | 13.3 (7.9-20.1) | 1895  (668-2912) | 10.6 (3.7-16.3) | 1837  (1077-2877) | 10.3 (6-16.1) | 147 (48-360) | 0.8 (0.3-2) | 306 (25-909) | 1.7 (0.1-5.1) | 108 (24-222) | 0.6 (0.1-1.2) | |
| Philippines | 20681  (14107-28160) | 35.4 (24.1-48.2) | 12669  (4893-18538) | 21.7 (8.4-31.7) | 24540  (18156-33005) | 42 (31.1-56.5) | 5942  (2814-9243) | 10.2 (4.8-15.8) | 3195  (366-8102) | 5.5 (0.6-13.9) | 1798  (212-3375) | 3.1 (0.4-5.8) | |
| Poland | 3973  (2105-6019) | 21.8 (11.6-33.1) | 4368  (1622-6009) | 24 (8.9-33) | 1631  (725-2939) | 9 (4-16.1) | 263 (95-615) | 1.4 (0.5-3.4) | 2499  (925-4206) | 13.7 (5.1-23.1) | 1122  (209-1933) | 6.2 (1.1-10.6) | |
| Portugal | 707 (261-1179) | 15.1 (5.6-25.1) | 1255  (466-1651) | 26.8 (9.9-35.2) | 447 (203-820) | 9.5 (4.3-17.5) | 124 (40-296) | 2.6 (0.8-6.3) | 879 (389-1357) | 18.7 (8.3-28.9) | 335 (62-554) | 7.1 (1.3-11.8) | |
| Puerto Rico | 425 (251-650) | 26.8 (15.9-41) | 387 (144-568) | 24.4 (9.1-35.8) | 243 (129-410) | 15.3 (8.1-25.9) | 46 (14-107) | 2.9 (0.9-6.7) | 72 (6-201) | 4.5 (0.4-12.7) | 38 (5-78) | 2.4 (0.3-4.9) | |
| Qatar | 162 (88-256) | 7.5 (4.1-11.8) | 178 (66-261) | 8.2 (3.1-12.1) | 55 (26-100) | 2.6 (1.2-4.7) | 3 (2-5) | 0.1 (0.1-0.2) | 36 (4-93) | 1.7 (0.2-4.3) | 15 (2-30) | 0.7 (0.1-1.4) | |
| South Korea | 4842  (3085-6743) | 18.6 (11.8-25.9) | 3694  (1390-5242) | 14.2 (5.3-20.1) | 2992  (1712-4555) | 11.5 (6.6-17.5) | 1229  (462-2062) | 4.7 (1.8-7.9) | 2175  (675-3789) | 8.4 (2.6-14.5) | 1128  (180-1921) | 4.3 (0.7-7.4) | |
| Moldova | 367 (166-561) | 20 (9-30.5) | 451 (170-622) | 24.5 (9.3-33.8) | 268 (144-436) | 14.6 (7.8-23.7) | 37 (12-89) | 2 (0.7-4.8) | 80 (6-224) | 4.4 (0.3-12.2) | 169 (52-271) | 9.2 (2.8-14.8) | |
| Romania | 890 (230-1781) | 10.3 (2.7-20.6) | 2640  (940-3711) | 30.5 (10.9-42.9) | 375 (165-652) | 4.3 (1.9-7.5) | 134 (47-334) | 1.5 (0.5-3.9) | 1591  (499-2779) | 18.4 (5.8-32.1) | 1036  (300-1694) | 12 (3.5-19.6) | |
| Russia | 9766  (3602-16232) | 14.3 (5.3-23.7) | 18279  (6833-24807) | 26.7 (10-36.2) | 7394  (3669-12985) | 10.8 (5.4-19) | 2142  (760-4447) | 3.1 (1.1-6.5) | 6120  (1422-12275) | 8.9 (2.1-17.9) | 6435  (1946-10299) | 9.4 (2.8-15) | |
| Rwanda | 695 (392-1117) | 10.7 (6.1-17.3) | 604 (209-978) | 9.3 (3.2-15.1) | 717 (446-1093) | 11.1 (6.9-16.9) | 8 (5-12) | 0.1 (0.1-0.2) | 64 (7-183) | 1 (0.1-2.8) | 72 (9-152) | 1.1 (0.1-2.3) | |
| Saint Kitts and Nevis | 6 (2-10) | 18.4 (5.3-32.3) | 5 (1-9) | 17.1 (4.7-29.7) | 5 (1-8) | 15.6 (4.6-26.2) | 2 (1-4) | 6.9 (1.9-12.5) | 1 (0-3) | 2.9 (0.2-9.9) | 0 (0-1) | 1.4 (0.1-3.3) | |
| Saint Lucia | 20 (13-27) | 20.8 (13.6-28.7) | 18 (7-25) | 19.4 (7.7-26.7) | 14 (9-20) | 14.7 (9.2-21.5) | 4 (2-7) | 4.3 (1.6-7.7) | 4 (0-10) | 4 (0.4-10.3) | 1 (0-3) | 1.6 (0.2-3) | |
| Saint Vincent and the Grenadines | 14 (9-19) | 24 (15.8-33.1) | 12 (5-17) | 21.7 (8.1-30) | 11 (7-16) | 19.2 (13-27.2) | 2 (1-4) | 3.4 (1.1-6.9) | 2 (0-5) | 3.4 (0.3-9.5) | 1 (0-2) | 2 (0.3-4) | |
| Samoa | 15 (7-23) | 14.3 (7.2-22.5) | 14 (5-22) | 13.5 (4.8-21.1) | 16 (8-24) | 14.9 (8.1-22.9) | 0 (0-0) | 0.2 (0.1-0.2) | 3 (0-8) | 2.8 (0.3-7.5) | 1 (0-2) | 0.9 (0.1-1.9) | |
| San Marino | 2 (1-4) | 12.9 (4.9-25.6) | 3 (1-5) | 20 (7.4-35.6) | 0 (0-1) | 2.8 (0.9-6.2) | 0 (0-1) | 2.1 (0.6-5.2) | 2 (1-4) | 11.1 (3.3-23.9) | 1 (0-2) | 7.8 (2.5-15.6) | |
| Sao Tome and Principe | 17 (10-28) | 16.3 (9.1-26) | 13 (5-22) | 12.5 (4.5-20.2) | 19 (11-29) | 17.3 (10.3-27.1) | 0 (0-0) | 0.2 (0.1-0.3) | 1 (0-2) | 0.8 (0.1-2) | 3 (0-5) | 2.5 (0.3-5.1) | |
| Saudi Arabia | 4139  (2320-6602) | 17 (9.5-27.1) | 4037  (1403-6159) | 16.6 (5.8-25.3) | 2787  (1510-4733) | 11.4 (6.2-19.4) | 205 (69-538) | 0.8 (0.3-2.2) | 563 (44-1675) | 2.3 (0.2-6.9) | 306 (47-631) | 1.3 (0.2-2.6) | |
| Senegal | 599 (334-911) | 8.2 (4.6-12.4) | 486 (176-755) | 6.6 (2.4-10.3) | 730 (453-1057) | 9.9 (6.2-14.4) | 16 (7-38) | 0.2 (0.1-0.5) | 72 (6-212) | 1 (0.1-2.9) | 108 (15-211) | 1.5 (0.2-2.9) | |
| Serbia | 954 (438-1540) | 23.8 (10.9-38.5) | 1233  (447-1823) | 30.8 (11.2-45.5) | 599 (290-1100) | 15 (7.3-27.5) | 136 (42-315) | 3.4 (1-7.9) | 255 (24-683) | 6.4 (0.6-17.1) | 313 (58-572) | 7.8 (1.5-14.3) | |
| Seychelles | 35 (26-46) | 65.4 (48.6-84.6) | 19 (7-27) | 34.2 (12.7-49) | 22 (15-31) | 40.9 (28.3-56.4) | 3 (1-6) | 5.6 (1.9-11.7) | 3 (0-7) | 4.8 (0.5-12.8) | 1 (0-2) | 1.8 (0.6-3.1) | |
| Sierra Leone | 293 (171-451) | 7.1 (4.1-10.9) | 222 (80-351) | 5.3 (1.9-8.5) | 403 (255-585) | 9.7 (6.1-14.1) | 78 (32-134) | 1.9 (0.8-3.2) | 20 (3-53) | 0.5 (0.1-1.3) | 44 (5-87) | 1 (0.1-2.1) | |
| Singapore | 429 (275-589) | 14 (8.9-19.2) | 351 (128-483) | 11.4 (4.2-15.7) | 236 (131-377) | 7.7 (4.3-12.2) | 67 (23-135) | 2.2 (0.8-4.4) | 165 (36-315) | 5.4 (1.2-10.2) | 77 (8-137) | 2.5 (0.3-4.4) | |
| Slovakia | 736 (419-1129) | 28 (15.9-42.9) | 752 (268-1119) | 28.6 (10.2-42.5) | 463 (241-775) | 17.6 (9.2-29.5) | 167 (59-320) | 6.3 (2.3-12.2) | 293 (66-607) | 11.1 (2.5-23.1) | 276 (79-461) | 10.5 (3-17.5) | |
| Slovenia | 104 (41-183) | 11.7 (4.5-20.4) | 160 (57-244) | 17.9 (6.4-27.2) | 40 (17-76) | 4.5 (1.9-8.4) | 18 (5-42) | 2.1 (0.6-4.7) | 81 (21-154) | 9 (2.3-17.2) | 76 (28-135) | 8.5 (3.1-15.1) | |
| Solomon Islands | 89 (44-140) | 27 (13.3-42.6) | 72 (28-116) | 22.1 (8.4-35.2) | 99 (51-147) | 30.1 (15.5-44.9) | 1 (1-3) | 0.4 (0.2-0.8) | 9 (1-27) | 2.8 (0.2-8.2) | 4 (1-9) | 1.3 (0.2-2.7) | |
| Somalia | 657 (327-1303) | 6.9 (3.5-13.8) | 574 (192-1139) | 6.1 (2-12) | 909 (523-1769) | 9.6 (5.5-18.7) | 211 (73-437) | 2.2 (0.8-4.6) | 103 (6-345) | 1.1 (0.1-3.6) | 59 (7-149) | 0.6 (0.1-1.6) | |
| South Africa | 4232  (2554-6087) | 13.9 (8.4-20) | 3250  (1227-4737) | 10.7 (4-15.6) | 5724  (4511-7262) | 18.8 (14.8-23.8) | 309 (120-674) | 1 (0.4-2.2) | 1204  (232-2614) | 4 (0.8-8.6) | 481 (58-877) | 1.6 (0.2-2.9) | |
| South Sudan | 481 (234-833) | 11.4 (5.6-19.7) | 384 (136-673) | 9.1 (3.2-16) | 526 (294-882) | 12.5 (7-20.9) | 9 (4-23) | 0.2 (0.1-0.6) | 61 (5-188) | 1.4 (0.1-4.4) | 60 (6-137) | 1.4 (0.2-3.2) | |
| Spain | 2240  (743-3692) | 10.8 (3.6-17.9) | 4129  (1539-5561) | 20 (7.4-26.9) | 2067  (1041-3573) | 10 (5-17.3) | 682 (233-1405) | 3.3 (1.1-6.8) | 2301  (753-3891) | 11.1 (3.6-18.8) | 1743  (639-2711) | 8.4 (3.1-13.1) | |
| Sri Lanka | 1065  (650-1551) | 9.8 (6-14.2) | 792 (290-1208) | 7.3 (2.7-11.1) | 1203  (803-1729) | 11 (7.4-15.9) | 81 (25-192) | 0.7 (0.2-1.8) | 43 (10-104) | 0.4 (0.1-1) | 45 (12-91) | 0.4 (0.1-0.8) | |
| Sudan | 1962  (1017-3329) | 9.4 (4.9-16) | 2227  (1020-3731) | 10.7 (4.9-17.9) | 2187  (1233-3567) | 10.5 (5.9-17.1) | 269 (76-613) | 1.3 (0.4-2.9) | 249 (20-763) | 1.2 (0.1-3.7) | 110 (19-255) | 0.5 (0.1-1.2) | |
| Suriname | 75 (48-107) | 26.2 (16.7-37.3) | 61 (24-89) | 21.3 (8.2-30.8) | 78 (54-106) | 26.9 (18.8-36.9) | 25 (12-41) | 8.8 (4.1-14.2) | 8 (1-22) | 2.8 (0.3-7.5) | 6 (1-13) | 2.2 (0.3-4.5) | |
| Sweden | 273 (94-504) | 6.1 (2.1-11.2) | 739 (286-964) | 16.5 (6.4-21.5) | 93 (43-161) | 2.1 (1-3.6) | 94 (33-197) | 2.1 (0.7-4.4) | 387 (131-644) | 8.6 (2.9-14.4) | 335 (125-577) | 7.5 (2.8-12.9) | |
| Switzerland | 318 (127-503) | 8 (3.2-12.6) | 520 (206-689) | 13 (5.2-17.3) | 87 (35-150) | 2.2 (0.9-3.8) | 114 (41-205) | 2.9 (1-5.1) | 314 (122-492) | 7.9 (3.1-12.3) | 190 (64-293) | 4.8 (1.6-7.3) | |
| Syria | 639 (331-1019) | 8.6 (4.5-13.7) | 811 (366-1228) | 10.9 (4.9-16.5) | 552 (327-870) | 7.4 (4.4-11.7) | 74 (21-174) | 1 (0.3-2.3) | 102 (7-302) | 1.4 (0.1-4.1) | 45 (8-98) | 0.6 (0.1-1.3) | |
| Taiwan | 6181  (3616-9157) | 52.9 (30.9-78.4) | 5488  (1894-8125) | 47 (16.2-69.5) | 3556  (1969-5945) | 30.4 (16.8-50.9) | 1007  (347-1991) | 8.6 (3-17) | 3277  (1095-5831) | 28 (9.4-49.9) | 869 (89-1661) | 7.4 (0.8-14.2) | |
| Tajikistan | 718 (416-1085) | 14.5 (8.4-21.8) | 917 (427-1294) | 18.5 (8.6-26) | 923 (660-1264) | 18.6 (13.3-25.4) | 175 (65-313) | 3.5 (1.3-6.3) | 104 (9-291) | 2.1 (0.2-5.8) | 154 (24-277) | 3.1 (0.5-5.6) | |
| Thailand | 8789  (5375-13111) | 24.6 (15.1-36.7) | 6020  (2165-9330) | 16.9 (6.1-26.1) | 10638  (7022-15321) | 29.8 (19.7-42.9) | 2656  (1153-4665) | 7.4 (3.2-13.1) | 1203 (93-3667) | 3.4 (0.3-10.3) | 353 (86-696) | 1 (0.2-1.9) | |
| Timor-Leste | 137 (35-211) | 21.1 (5.4-32.3) | 69 (18-117) | 10.5 (2.7-18) | 135 (36-204) | 20.7 (5.6-31.3) | 14 (3-29) | 2.1 (0.5-4.5) | 11 (1-34) | 1.7 (0.1-5.3) | 3 (1-7) | 0.5 (0.1-1) | |
| Togo | 366 (209-572) | 9.3 (5.3-14.6) | 279 (95-438) | 7.1 (2.4-11.2) | 481 (297-700) | 12.2 (7.6-17.8) | 6 (3-12) | 0.1 (0.1-0.3) | 29 (3-78) | 0.7 (0.1-2) | 57 (7-117) | 1.5 (0.2-3) | |
| Tokelau | 0 (0-0) | 19.8 (9.9-32.3) | 0 (0-0) | 17.3 (6.1-29.4) | 0 (0-0) | 22.5 (12.2-36.4) | 0 (0-0) | 0.3 (0.1-0.7) | 0 (0-0) | 3.5 (0.3-9.8) | 0 (0-0) | 1.1 (0.2-2.6) | |
| Tonga | 4 (3-7) | 9.1 (5.3-14) | 4 (1-6) | 8 (2.9-12.6) | 5 (3-8) | 10.9 (7.1-16.1) | 0 (0-0) | 0.2 (0.1-0.4) | 1 (0-2) | 1.5 (0.1-4.3) | 0 (0-1) | 0.5 (0.1-1.1) | |
| Trinidad and Tobago | 237 (152-338) | 34.1 (21.9-48.7) | 162 (61-251) | 23.3 (8.7-36.1) | 107 (59-171) | 15.4 (8.4-24.6) | 23 (7-53) | 3.3 (1.1-7.6) | 23 (2-70) | 3.4 (0.3-10.1) | 16 (2-33) | 2.3 (0.3-4.8) | |
| Tunisia | 642 (298-1086) | 10.6 (4.9-18) | 905 (397-1349) | 15 (6.6-22.3) | 421 (203-731) | 7 (3.4-12.1) | 23 (10-58) | 0.4 (0.2-1) | 112 (9-330) | 1.9 (0.1-5.5) | 62 (9-137) | 1 (0.1-2.3) | |
| Turkey | 6178  (2707-9873) | 13.5 (5.9-21.6) | 10288  (5158-14392) | 22.5 (11.3-31.5) | 3190  (1452-5951) | 7 (3.2-13) | 167 (97-347) | 0.4 (0.2-0.8) | 1262 (99-3561) | 2.8 (0.2-7.8) | 673 (99-1339) | 1.5 (0.2-2.9) | |
| Turkmenistan | 167 (53-289) | 6.3 (2-11) | 427 (206-594) | 16.2 (7.8-22.5) | 160 (76-294) | 6.1 (2.9-11.1) | 52 (17-108) | 2 (0.7-4.1) | 219 (87-366) | 8.3 (3.3-13.9) | 103 (26-175) | 3.9 (1-6.6) | |
| Tuvalu | 1 (1-2) | 20.6 (11.5-31.8) | 1 (0-2) | 18.1 (6.5-28.8) | 2 (1-2) | 25.6 (16.2-38) | 0 (0-0) | 0.6 (0.2-1.4) | 0 (0-1) | 3.3 (0.3-9) | 0 (0-0) | 1.1 (0.2-2.4) | |
| Uganda | 2322  (1294-3657) | 12.3 (6.9-19.4) | 1899  (685-2932) | 10.1 (3.6-15.5) | 2705  (1799-3909) | 14.3 (9.5-20.7) | 53 (25-128) | 0.3 (0.1-0.7) | 298 (24-883) | 1.6 (0.1-4.7) | 253 (30-545) | 1.3 (0.2-2.9) | |
| Ukraine | 5771  (2602-9303) | 27.8 (12.5-44.8) | 8106  (3113-11164) | 39 (15-53.7) | 3625  (1781-6320) | 17.4 (8.6-30.4) | 704 (232-1706) | 3.4 (1.1-8.2) | 1797  (216-4582) | 8.6 (1-22) | 1815  (293-3209) | 8.7 (1.4-15.4) | |
| United Arab Emirates | 1259  (668-2059) | 18.1 (9.6-29.6) | 1297  (466-2031) | 18.6 (6.7-29.1) | 630 (286-1148) | 9 (4.1-16.5) | 71 (23-188) | 1 (0.3-2.7) | 239 (19-676) | 3.4 (0.3-9.7) | 125 (15-265) | 1.8 (0.2-3.8) | |
| UK | 4516  (2217-6719) | 14.8 (7.3-22) | 5791  (2159-7462) | 19 (7.1-24.5) | 1375  (588-2493) | 4.5 (1.9-8.2) | 863 (331-1682) | 2.8 (1.1-5.5) | 2018  (559-3857) | 6.6 (1.8-12.7) | 2627  (989-4461) | 8.6 (3.2-14.6) | |
| Tanzania | 2920  (1619-4630) | 11 (6.1-17.4) | 2172  (750-3669) | 8.1 (2.8-13.8) | 3818  (2464-5787) | 14.3 (9.2-21.7) | 82 (32-195) | 0.3 (0.1-0.7) | 335 (30-962) | 1.3 (0.1-3.6) | 336 (38-704) | 1.3 (0.1-2.6) | |
| USA | 19654  (8132-31715) | 13.1 (5.4-21.1) | 31799  (11872-41867) | 21.1 (7.9-27.8) | 8275  (3925-14325) | 5.5 (2.6-9.5) | 3951 (1423-7927) | 2.6 (0.9-5.3) | 18324  (6382-29665) | 12.2 (4.2-19.7) | 15933  (5836-27440) | 10.6 (3.9-18.2) | |
| United States Virgin Islands | 16 (9-25) | 36.6 (21.2-57.8) | 15 (5-22) | 33.4 (11.7-51.7) | 8 (4-15) | 19 (9.4-35.1) | 1 (0-3) | 3.3 (1-8.1) | 3 (0-8) | 6.5 (0.6-18.1) | 1 (0-3) | 3.4 (0.4-7.5) | |
| Uruguay | 263 (97-417) | 15.9 (5.9-25.3) | 474 (184-624) | 28.7 (11.2-37.8) | 163 (75-289) | 9.9 (4.6-17.5) | 58 (18-122) | 3.5 (1.1-7.4) | 290 (119-455) | 17.6 (7.2-27.6) | 149 (45-234) | 9.1 (2.7-14.2) | |
| Uzbekistan | 1916  (889-3001) | 10.6 (4.9-16.6) | 3170  (1516-4261) | 17.5 (8.4-23.6) | 1328  (692-2261) | 7.3 (3.8-12.5) | 273 (90-614) | 1.5 (0.5-3.4) | 845 (165-1770) | 4.7 (0.9-9.8) | 683 (148-1180) | 3.8 (0.8-6.5) | |
| Vanuatu | 24 (13-38) | 16.7 (9-26.2) | 21 (8-34) | 14.7 (5.2-23.2) | 32 (20-49) | 22.2 (13.9-33.7) | 0 (0-1) | 0.3 (0.1-0.6) | 6 (1-14) | 3.8 (0.5-9.6) | 1 (0-3) | 0.9 (0.2-1.9) | |
| Venezuela | 2744  (1650-4067) | 19.1 (11.5-28.3) | 2057  (772-3156) | 14.3 (5.4-21.9) | 2537  (1605-3686) | 17.6 (11.1-25.6) | 510 (176-993) | 3.5 (1.2-6.9) | 531 (58-1392) | 3.7 (0.4-9.7) | 294 (31-590) | 2 (0.2-4.1) | |
| Vietnam | 15319  (9729-23075) | 28.9 (18.3-43.5) | 9953  (3713-16023) | 18.8 (7-30.2) | 19749  (13210-27832) | 37.2 (24.9-52.5) | 5485  (2613-8920) | 10.3 (4.9-16.8) | 3540  (488-8513) | 6.7 (0.9-16.1) | 366 (209-584) | 0.7 (0.4-1.1) | |
| Yemen | 1467  (817-2340) | 9.4 (5.2-15) | 1613  (763-2563) | 10.3 (4.9-16.4) | 1907  (1208-2845) | 12.2 (7.7-18.2) | 273 (93-532) | 1.7 (0.6-3.4) | 155 (14-448) | 1 (0.1-2.9) | 68 (15-146) | 0.4 (0.1-0.9) | |
| Zambia | 1557  (918-2461) | 17.2 (10.2-27.3) | 1075  (392-1738) | 11.9 (4.3-19.3) | 2230  (1432-3256) | 24.7 (15.9-36.1) | 52 (19-135) | 0.6 (0.2-1.5) | 183 (14-537) | 2 (0.2-5.9) | 150 (18-323) | 1.7 (0.2-3.6) | |
| Zimbabwe | 1310  (738-2074) | 17.4 (9.8-27.6) | 987 (344-1586) | 13.1 (4.6-21.1) | 1917  (1203-2747) | 25.5 (16-36.5) | 62 (19-155) | 0.8 (0.3-2.1) | 163 (13-487) | 2.2 (0.2-6.5) | 100 (15-212) | 1.3 (0.2-2.8) | |

**Supplementary Table S9. Global and regional age-specific summary exposure value rates of a diet low in milk attributable to early-onset colorectal cancer in 1990 and 2019, and its temporal trends from 1990 to 2019, by sex**

| Characteristics | Male | | |  | Female | | |
| --- | --- | --- | --- | --- | --- | --- | --- |
|  | Age-specific SEV rate in 1990 (95% UI) | Age-specific SEV rate in 2019 (95% UI) | ARC (%) (95% UI) |  | Age-specific SEV rate in 1990 (95% UI) | Age-specific SEV rate in 2019 (95% UI) | ARC (%) (95% UI) |
| Overall | 82.6 (71.5-91.4) | 84.8 (74.3-93.2) | 0.03 (0.01-0.05) |  | 82.1 (70.8-91.2) | 84.4 (73.7-92.9) | 0.03 (0.01-0.05) |
| Socio-demographic index |  |  |  |  |  |  |  |
| High | 64.3 (44.8-80.4) | 61.6 (44-76.8) | −0.04 (−0.09-0) |  | 63.8 (42.8-80.2) | 60.6 (42.4-76.1) | −0.05 (−0.1-0.01) |
| High -middle | 71.5 (57.7-82.5) | 77.7 (65.2-87.8) | 0.09 (0.05-0.15) |  | 71.9 (57.9-83.3) | 76.9 (63.6-87.5) | 0.07 (0.03-0.12) |
| Middle | 92 (83.5-98.5) | 91.3 (82.1-98.2) | −0.01 (−0.02-0) |  | 91.8 (83.1-98.5) | 90.8 (81.6-97.9) | −0.01 (−0.02-0) |
| Low-middle | 93.6 (85.8-99.3) | 91.8 (82.6-98.6) | −0.02 (−0.04-−0.01) |  | 93.1 (84.8-99.2) | 91.1 (81.4-98.4) | −0.02 (−0.04-−0.01) |
| Low | 93.9 (85.8-99.8) | 92.4 (83-99.4) | −0.02 (−0.04-0) |  | 93 (84-99.6) | 91.3 (80.8-99.1) | −0.02 (−0.04-0) |
| GBD regions |  |  |  |  |  |  |  |
| High-income Asia Pacific | 87.2 (70.4-99.8) | 85.6 (66.5-99.9) | −0.02 (−0.06-0) |  | 84.3 (64-99.7) | 84.4 (64.6-99.8) | 0 (−0.02-0.02) |
| Central Asia | 50.1 (29.9-68.5) | 50.5 (31.2-67.1) | 0.01 (−0.09-0.18) |  | 49.4 (29.5-67.3) | 49.6 (31.1-65.9) | 0 (−0.09-0.18) |
| East Asia | 92.3 (84.2-98.5) | 95.4 (87.6-100) | 0.03 (0-0.09) |  | 92.7 (84.6-98.9) | 95 (86.6-100) | 0.03 (−0.01-0.07) |
| South Asia | 94.7 (87.3-99.8) | 93.2 (84.5-99.5) | −0.02 (−0.03-0) |  | 94.5 (86.7-99.8) | 92.8 (83.7-99.4) | −0.02 (−0.04-0) |
| Southeast Asia | 99.6 (98.6-100) | 98.9 (96.6-100) | −0.01 (−0.02-0) |  | 99.6 (98.6-100) | 98.8 (96.3-100) | −0.01 (−0.02-0) |
| Australasia | 42.8 (19.6-62.9) | 23.5 (7.7-42.6) | −0.45 (−0.73-−0.17) |  | 41.8 (18.9-61.5) | 22.4 (7.8-41.2) | −0.46 (−0.72-−0.19) |
| Caribbean | 88.1 (73.1-99.8) | 90 (76.4-99.9) | 0.02 (0-0.06) |  | 88 (72.7-99.8) | 90.1 (76.8-99.8) | 0.02 (0-0.07) |
| Central Europe | 58.1 (34.1-77.6) | 59.3 (37.1-77.2) | 0.02 (−0.07-0.19) |  | 61.1 (35.1-82) | 65.6 (40.2-85.7) | 0.07 (0-0.24) |
| Eastern Europe | 37.4 (16.2-56.2) | 46 (23.7-65.2) | 0.23 (0.05-0.65) |  | 35.3 (13.9-53.7) | 43.5 (22-63.5) | 0.23 (0.03-0.71) |
| Western Europe | 58.4 (34.5-77.5) | 52.4 (30-71.3) | −0.1 (−0.24-−0.03) |  | 58.4 (34.3-77.3) | 52.6 (30.6-71.8) | −0.1 (−0.23-−0.02) |
| Andean Latin America | 89 (77.1-98.4) | 89.5 (76.7-98.9) | 0 (−0.01-0.02) |  | 87.3 (73.5-97.9) | 88 (74.3-98.7) | 0.01 (−0.01-0.03) |
| Central Latin America | 82 (62-97.9) | 76.8 (54.5-94.7) | −0.06 (−0.15-−0.01) |  | 81.7 (61.1-97.7) | 76.5 (55-94.4) | −0.06 (−0.14-−0.02) |
| Southern Latin America | 77.9 (56.7-95) | 77.7 (56.5-95.3) | 0 (−0.03-0.01) |  | 77.6 (56.4-94.7) | 77.2 (55.4-95) | −0.01 (−0.03-0.01) |
| Tropical Latin America | 75.2 (49.5-95.5) | 64.9 (38.2-86) | −0.14 (−0.29-−0.03) |  | 74.8 (48.6-95.2) | 64.5 (37.3-85.5) | −0.14 (−0.3-−0.03) |
| North Africa and Middle East | 83.9 (71.5-93.8) | 83 (66.5-96.4) | −0.01 (−0.08-0.04) |  | 83.5 (70.9-93.6) | 82.7 (66.2-95.7) | −0.01 (−0.07-0.04) |
| High-income North America | 51.3 (28.5-71.5) | 44.5 (22.1-64.7) | −0.13 (−0.32-−0.02) |  | 51.5 (28.5-71.3) | 44.8 (22.2-64.5) | −0.13 (−0.31-−0.02) |
| Oceania | 96.3 (89.9-100) | 97.1 (91.5-100) | 0.01 (0-0.02) |  | 96.3 (89.8-100) | 97 (91.4-100) | 0.01 (0-0.02) |
| Central Sub-Saharan Africa | 98.9 (97-100) | 99.1 (97.4-100) | 0 (0-0) |  | 99 (97.2-100) | 99 (97.1-100) | 0 (0-0) |
| Eastern Sub-Saharan Africa | 91.8 (81.3-99.3) | 89.9 (76.8-99.6) | −0.02 (−0.06-0.01) |  | 91.8 (81.3-99.3) | 89.7 (76.4-99.6) | −0.02 (−0.07-0) |
| Southern Sub-Saharan Africa | 89.1 (73.9-100) | 86.1 (67.7-99.8) | −0.03 (−0.1-0) |  | 89 (73.5-100) | 86 (68.1-99.8) | −0.03 (-0.09-0) |
| Western Sub-Saharan Africa | 97.9 (95-99.8) | 97.3 (93.9-99.8) | −0.01 (−0.01-0) |  | 97.5 (94.2-99.8) | 97.3 (93.8-99.8) | 0 (−0.01-0) |

**Supplementary Table S10. Global and regional age-specific summary exposure value rates of a diet low in whole grains attributable to early-onset colorectal cancer in 1990 and 2019, and its temporal trends from 1990 to 2019, by sex**

| Characteristics | Male | | |  | Female | | |
| --- | --- | --- | --- | --- | --- | --- | --- |
|  | Age-specific SEV rate in 1990 (95% UI) | Age-specific SEV rate in 2019 (95% UI) | ARC (%) (95% UI) |  | Age-specific SEV rate in 1990 (95% UI) | Age-specific SEV rate in 2019 (95% UI) | ARC (%) (95% UI) |
| Overall | 80.8 (73.5-88) | 80.2 (72.8-87.7) | −0.01 (−0.01-0) |  | 79 (71.4-86.8) | 77.9 (69.9-86.1) | −0.01 (−0.02-−0.01) |
| Socio-demographic index |  |  |  |  |  |  |  |
| High | 78.9 (70.6-87.4) | 83.2 (76-90.4) | 0.05 (0.03-0.08) |  | 73.8 (63.6-83.8) | 79.1 (70.4-87.4) | 0.07 (0.04-0.11) |
| High -middle | 88.4 (83.6-93.4) | 85.5 (79.5-91.7) | −0.03 (−0.05-−0.02) |  | 90.3 (86.3-94.3) | 86.4 (80.7-91.9) | −0.04 (−0.07-−0.02) |
| Middle | 78.7 (70.7-86.7) | 77.8 (69.5-86.2) | −0.01 (−0.02-0) |  | 76.7 (68.2-85.3) | 76.1 (67.5-84.9) | −0.01 (−0.02-0) |
| Low-middle | 75.4 (66.6-84.2) | 74.9 (66.5-83.7) | −0.01 (−0.02-0.01) |  | 72.8 (64-82.1) | 72.2 (63.4-81.9) | −0.01 (−0.03-0.01) |
| Low | 83.6 (76.8-90.2) | 83.7 (76.8-90.3) | 0 (−0.01-0.01) |  | 81.3 (73.9-88.6) | 81.3 (73.6-88.7) | 0 (−0.01-0.01) |
| GBD regions |  |  |  |  |  |  |  |
| High-income Asia Pacific | 67.5 (55.5-79.8) | 75 (64.8-85.1) | 0.11 (0.06-0.18) |  | 63.7 (51.3-77.2) | 72.2 (61.3-82.8) | 0.13 (0.07-0.22) |
| Central Asia | 99.9 (100-100) | 99.9 (100-100) | 0 (0-0) |  | 99.9 (100-100) | 99.9 (100-100) | 0 (0-0) |
| East Asia | 83 (75.8-90.3) | 84.4 (77.6-91.2) | 0.02 (0-0.04) |  | 81.3 (73.3-89.1) | 82.8 (75.1-90.2) | 0.02 (0-0.05) |
| South Asia | 73.2 (64.1-82.2) | 73.5 (64.4-82.7) | 0 (−0.01-0.02) |  | 70.8 (61.9-80.7) | 70.8 (61.6-80.8) | 0 (−0.02-0.02) |
| Southeast Asia | 67.4 (55.5-80.1) | 64.9 (51.9-78.5) | −0.04 (−0.06-−0.02) |  | 61.6 (48.6-76.2) | 58.8 (45.2-74.4) | −0.04 (−0.09-−0.01) |
| Australasia | 77.1 (67.5-86.5) | 81.3 (73-89.4) | 0.05 (0.03-0.09) |  | 74.3 (63.8-84.5) | 79 (70.1-87.7) | 0.06 (0.03-0.1) |
| Caribbean | 87.5 (81.8-93.2) | 83.7 (76.5-90.7) | −0.04 (−0.07-−0.02) |  | 85.6 (79.1-91.9) | 81.6 (73.7-89.4) | −0.05 (−0.07-−0.03) |
| Central Europe | 91 (86.8-95.2) | 84.7 (77.6-91.9) | −0.07 (−0.11-−0.03) |  | 88.4 (83.2-93.6) | 85.2 (78.8-91.4) | −0.04 (−0.06-−0.02) |
| Eastern Europe | 97.5 (96-99.2) | 91.6 (87.3-95.7) | −0.06 (−0.09-−0.03) |  | 96.7 (94.7-98.7) | 89.7 (84.5-94.5) | −0.07 (−0.11-−0.04) |
| Western Europe | 86.7 (80.7-92.7) | 89.2 (84.2-94.3) | 0.03 (0.01-0.05) |  | 91.9 (88.3-95.4) | 91.6 (88-95.4) | 0 (−0.01-0.01) |
| Andean Latin America | 84.8 (78-91.6) | 80.1 (71.6-88.6) | −0.06 (−0.09-−0.03) |  | 82.8 (75.2-90.4) | 77.4 (67.8-86.5) | −0.07 (−0.1-−0.03) |
| Central Latin America | 71.5 (61.6-82) | 71 (61.1-81.7) | −0.01 (−0.02-0.01) |  | 67.6 (57.1-79) | 67.7 (57.4-78.3) | 0 (−0.02-0.03) |
| Southern Latin America | 99.6 (99.3-99.9) | 91.1 (86.2-95.7) | −0.09 (−0.13-−0.04) |  | 98.8 (98-99.6) | 89 (83.1-94.7) | −0.1 (−0.15-−0.05) |
| Tropical Latin America | 79.8 (70.8-88.8) | 74.4 (63.8-85.3) | −0.07 (−0.11-−0.03) |  | 78.2 (69.9-87.1) | 72.8 (61.5-84.2) | −0.07 (−0.14-−0.01) |
| North Africa and Middle East | 96.9 (95.5-98.3) | 96 (94.2-97.8) | −0.01 (−0.01-0) |  | 96.7 (95.3-98.2) | 95.8 (94.1-97.6) | −0.01 (−0.01-0) |
| High-income North America | 80.3 (71.9-88.8) | 81.7 (73.6-89.6) | 0.02 (0-0.05) |  | 77.9 (68.4-86.9) | 80 (71.1-88.5) | 0.03 (0-0.07) |
| Oceania | 92.3 (88.3-96.2) | 91.3 (87-95.5) | −0.01 (−0.03-0) |  | 91.9 (87.8-96) | 90.7 (86-95) | −0.01 (−0.03-0) |
| Central Sub-Saharan Africa | 76.9 (67.3-86) | 78.9 (70.2-87.6) | 0.03 (0.01-0.05) |  | 73.8 (63.4-84) | 76.4 (67-85.9) | 0.04 (0.02-0.06) |
| Eastern Sub-Saharan Africa | 79.3 (71.1-87.2) | 79.2 (70.8-87.4) | 0 (−0.01-0.01) |  | 77 (68.4-85.6) | 76.3 (67-85.4) | −0.01 (−0.03-0) |
| Southern Sub-Saharan Africa | 65.5 (53.1-78.6) | 66.6 (54.6-79.8) | 0.02 (0-0.05) |  | 61.5 (48.8-75.4) | 62.7 (49.7-76.4) | 0.02 (−0.01-0.05) |
| Western Sub-Saharan Africa | 85.7 (79.3-91.9) | 82.3 (74.8-89.9) | −0.04 (−0.06-−0.02) |  | 84 (77-90.9) | 80 (71.3-88.1) | −0.05 (−0.07-−0.03) |

**Supplementary Table S11. Global and regional age-specific summary exposure value rates of a diet low in calcium attributable to early-onset colorectal cancer in 1990 and 2019, and its temporal trends from 1990 to 2019, by sex**

| Characteristics | Male | | |  | Female | | |
| --- | --- | --- | --- | --- | --- | --- | --- |
|  | Age-specific SEV rate in 1990 (95% UI) | Age-specific SEV rate in 2019 (95% UI) | ARC (%) (95% UI) |  | Age-specific SEV rate in 1990 (95% UI) | Age-specific SEV rate in 2019 (95% UI) | ARC (%) (95% UI) |
| Overall | 58.4 (49.2-70.4) | 50.9 (40.8-64.9) | −0.13 (−0.18-−0.07) |  | 53.3 (44.1-65.2) | 46.5 (36-60.9) | −0.13 (−0.18-−0.07) |
| Socio-demographic index |  |  |  |  |  |  |  |
| High | 24.5 (15.2-39.5) | 21.4 (12.7-35.7) | −0.13 (−0.17-−0.09) |  | 18.7 (10.9-31.4) | 17 (9.7-28.6) | −0.09 (−0.14-−0.05) |
| High -middle | 47.6 (36.3-63) | 36.3 (24.6-54.4) | −0.24 (−0.34-−0.13) |  | 41 (30.3-56.2) | 31.3 (20.6-47.9) | −0.24 (−0.34-−0.14) |
| Middle | 73.4 (64.1-85.4) | 58.2 (47.3-73.5) | −0.21 (−0.27-−0.12) |  | 68.5 (58.7-81.8) | 52.8 (41.3-68.5) | −0.23 (−0.3-−0.14) |
| Low-middle | 68.9 (59.9-81.2) | 58.6 (48.1-72.7) | −0.15 (−0.2-−0.09) |  | 65.9 (56.2-79.1) | 54.6 (43.7-69.8) | −0.17 (−0.23-−0.1) |
| Low | 81.7 (74-91.2) | 76.2 (67.2-87.8) | −0.07 (−0.09-−0.04) |  | 77.9 (68.9-89.4) | 71.1 (61.1-84.7) | −0.09 (−0.12-−0.05) |
| GBD regions |  |  |  |  |  |  |  |
| High-income Asia Pacific | 45 (31.5-64.8) | 39.5 (26-59.9) | −0.12 (−0.2-−0.06) |  | 38.2 (24.4-59.5) | 37.9 (24.4-59.2) | −0.01 (−0.06-0.04) |
| Central Asia | 36.4 (24.2-56) | 28.7 (18.3-45.7) | −0.21 (−0.28-−0.16) |  | 29.2 (17.7-48.3) | 21.8 (12.6-37.2) | −0.25 (−0.33-−0.18) |
| East Asia | 71.1 (59.6-85.5) | 49 (35.5-67.9) | −0.31 (−0.43-−0.16) |  | 65.7 (53.2-82.1) | 45.8 (32.6-65.4) | −0.3 (−0.42-−0.15) |
| South Asia | 63.7 (53.6-77.7) | 53.7 (42.6-68.4) | −0.16 (−0.21-−0.1) |  | 60.9 (50.2-75.8) | 49.9 (38.6-66) | −0.18 (−0.24-−0.11) |
| Southeast Asia | 95.1 (93.4-96.5) | 86.3 (79.1-94.2) | −0.09 (−0.15-−0.02) |  | 93.5 (90.7-96.3) | 81.8 (73.3-92) | −0.12 (−0.19-−0.04) |
| Australasia | 20.4 (10-37.9) | 20.4 (10.6-36.9) | 0 (−0.07-0.1) |  | 14.7 (6.1-28.2) | 14.7 (6.9-27.3) | 0 (−0.13-0.2) |
| Caribbean | 57.1 (45.9-73) | 55.7 (44.7-71) | −0.02 (−0.05-0) |  | 49.9 (38.4-66.8) | 49.6 (38.6-65.5) | −0.01 (−0.04-0.03) |
| Central Europe | 24.1 (13.3-42.9) | 19.1 (9.9-34.8) | −0.21 (−0.28-−0.16) |  | 17.8 (8.2-33.9) | 13.4 (6.1-25.6) | −0.25 (−0.33-−0.2) |
| Eastern Europe | 28.9 (16.7-49.5) | 22 (11.5-40.1) | −0.24 (−0.37-−0.13) |  | 22 (11.2-40.9) | 15.9 (7-30.7) | −0.28 (−0.47-−0.14) |
| Western Europe | 17.5 (8.7-32.3) | 14.7 (7-27.3) | −0.16 (−0.23-−0.1) |  | 11.9 (5.2-22.4) | 9.2 (4-16.9) | −0.22 (−0.32-−0.15) |
| Andean Latin America | 69.1 (57.9-84.8) | 56.1 (43.6-74.3) | −0.19 (−0.26-−0.1) |  | 61.8 (49.8-78.8) | 47.6 (34.4-66.6) | −0.23 (−0.32-−0.12) |
| Central Latin America | 58.8 (46.2-76.9) | 48 (34.8-67.1) | −0.18 (−0.26-−0.09) |  | 50.3 (37.2-69.2) | 39.5 (26.4-60.1) | −0.22 (−0.31-−0.11) |
| Southern Latin America | 42.1 (29-61.9) | 34.9 (22.3-55.5) | −0.17 (−0.26-−0.09) |  | 33.9 (21.3-54.9) | 27.2 (15.2-47.3) | −0.2 (−0.31-−0.1) |
| Tropical Latin America | 53.5 (40.7-72) | 33.6 (20.9-53.8) | −0.37 (−0.5-−0.22) |  | 44.6 (31.3-64.6) | 25.7 (13.8-45.6) | −0.42 (−0.58-−0.26) |
| North Africa and Middle East | 57.5 (46.4-72.3) | 47 (35.5-63.3) | −0.18 (−0.25-−0.1) |  | 51.5 (40-66.6) | 41.1 (29.8-58) | −0.2 (−0.27-−0.12) |
| High-income North America | 16.1 (7.8-29.7) | 12.8 (6.3-23.3) | −0.21 (−0.32-−0.1) |  | 11.6 (5-22.4) | 10.2 (4.3-19) | −0.12 (−0.26-0.03) |
| Oceania | 80.7 (71.8-91.4) | 83.6 (75.6-93) | 0.04 (0.01-0.06) |  | 75.3 (65.2-88.4) | 78.7 (69.1-90.4) | 0.05 (0.02-0.08) |
| Central Sub-Saharan Africa | 88.8 (82.2-95.7) | 90.4 (85.6-95.5) | 0.02 (0-0.05) |  | 84.7 (76.4-94) | 86.4 (79.8-94) | 0.02 (0-0.05) |
| Eastern Sub-Saharan Africa | 84.5 (78.4-92.2) | 78.2 (69.9-88.7) | −0.08 (−0.11-−0.04) |  | 80.6 (73.3-90) | 72.6 (63-85) | −0.1 (−0.14-−0.05) |
| Southern Sub-Saharan Africa | 82.9 (75.9-91.1) | 80.1 (72.3-89.7) | −0.03 (−0.06-−0.01) |  | 78.3 (70-88.6) | 75.2 (65.7-87.1) | −0.04 (−0.07-−0.01) |
| Western Sub-Saharan Africa | 87.8 (81.4-94.8) | 76.5 (66.7-89.1) | −0.13 (−0.19-−0.06) |  | 83.4 (75.2-92.8) | 70.2 (59.2-84.9) | −0.16 (−0.22-−0.08) |

**Supplementary Table S12. Global and regional age-specific summary exposure value rates of a diet low in fiber attributable to early-onset colorectal cancer in 1990 and 2019, and its temporal trends from 1990 to 2019, by sex**

| Characteristics | Male | | |  | Female | | |
| --- | --- | --- | --- | --- | --- | --- | --- |
|  | Age-specific SEV rate in 1990 (95% UI) | Age-specific SEV rate in 2019 (95% UI) | ARC (%) (95% UI) |  | Age-specific SEV rate in 1990 (95% UI) | Age-specific SEV rate in 2019 (95% UI) | ARC (%) (95% UI) |
| Overall | 40.7 (28.8-52.1) | 30.4 (20.2-40.5) | −0.25 (−0.32-−0.19) |  | 41.5 (29.5-53.1) | 31.2 (21.2-41.7) | −0.25 (−0.32-−0.19) |
| Socio-demographic index |  |  |  |  |  |  |  |
| High | 39.1 (25.8-52) | 30.8 (19.4-41.9) | −0.21 (−0.29-−0.15) |  | 39.9 (26-53.3) | 33.2 (21.1-45.4) | −0.17 (−0.23-−0.11) |
| High -middle | 33.2 (21.3-44.9) | 24.1 (13.8-34.9) | −0.27 (−0.4-−0.15) |  | 34.1 (21.7-46.1) | 25.5 (15-36.9) | −0.25 (−0.39-−0.14) |
| Middle | 44.2 (32.2-55.3) | 30.6 (20.7-41.1) | −0.31 (−0.4-−0.22) |  | 45.8 (33.6-57.1) | 31.2 (21.3-41.8) | −0.32 (−0.41-−0.23) |
| Low-middle | 49.5 (38.1-60.3) | 37.5 (27-47.9) | −0.24 (−0.32-−0.18) |  | 49.5 (38-60.4) | 37.9 (27.1-48.9) | −0.24 (−0.3-−0.18) |
| Low | 31.7 (20.8-42.1) | 27 (17.6-36.3) | −0.15 (−0.22-−0.07) |  | 30.8 (20.1-41.3) | 25.9 (16.4-35.4) | −0.16 (−0.23-−0.09) |
| GBD regions |  |  |  |  |  |  |  |
| High-income Asia Pacific | 34.7 (22.2-47.2) | 41.9 (28-54.3) | 0.21 (0.08-0.39) |  | 36 (23.4-49.3) | 45.6 (30.6-58.6) | 0.27 (0.12-0.45) |
| Central Asia | 43.5 (29.2-57.2) | 30.8 (19.4-43.2) | −0.29 (−0.38-−0.22) |  | 43.4 (28.4-57.5) | 30.4 (18.2-43.3) | −0.3 (−0.39-−0.22) |
| East Asia | 41.7 (26.1-56.8) | 24.4 (12.8-37.9) | −0.42 (−0.61-−0.2) |  | 44.6 (28.6-60) | 27.6 (15.5-41.2) | −0.38 (−0.58-−0.18) |
| South Asia | 48.9 (35.8-61.1) | 36.8 (25.4-48.8) | −0.25 (−0.35-−0.17) |  | 49.1 (35.8-61.6) | 37.4 (25.8-49.7) | −0.24 (−0.32-−0.15) |
| Southeast Asia | 75.4 (66.4-82.8) | 60.9 (47.5-72.3) | −0.19 (−0.29-−0.13) |  | 76.1 (67-83.6) | 61.1 (47.6-72.4) | −0.2 (−0.3-−0.13) |
| Australasia | 46.6 (31.5-60.4) | 39.4 (25.1-53.3) | −0.15 (−0.27-−0.05) |  | 46.7 (30.8-61.4) | 39.1 (24.9-53.7) | −0.16 (−0.29-−0.06) |
| Caribbean | 42 (27-55.4) | 28.7 (17.8-39.7) | −0.32 (−0.39-−0.25) |  | 41.9 (26.6-56.2) | 28.8 (17.4-40.4) | −0.31 (−0.4-−0.25) |
| Central Europe | 27.5 (16.6-39.2) | 24.8 (15-35.2) | −0.1 (−0.17-−0.02) |  | 27.2 (16-38.9) | 24.5 (14.5-35) | −0.1 (−0.17-−0.02) |
| Eastern Europe | 26.6 (16.2-37.8) | 26.5 (15.4-38) | 0 (−0.15-0.15) |  | 26.1 (15.2-37.8) | 26.1 (14.8-38.3) | 0 (−0.15-0.14) |
| Western Europe | 35.1 (22.3-47.9) | 33 (20.2-45.7) | −0.06 (−0.14-0.01) |  | 35.1 (21.7-48.5) | 33.3 (20.3-46.8) | −0.05 (−0.13-0.02) |
| Andean Latin America | 48.2 (32.2-62.6) | 34.9 (23.4-46.2) | −0.28 (−0.35-−0.21) |  | 48.4 (32.2-63.2) | 34.4 (22.7-46.3) | −0.29 (−0.37-−0.2) |
| Central Latin America | 23.3 (15.3-31.6) | 21.6 (13.6-30.3) | −0.08 (−0.18-0.02) |  | 22.8 (14.7-31) | 21 (13-30.4) | −0.08 (−0.17-0.02) |
| Southern Latin America | 48.7 (33.2-62.9) | 42.8 (27.6-57.2) | −0.12 (−0.22-−0.04) |  | 48.9 (32.8-63.7) | 42.7 (27.6-57.2) | −0.13 (−0.22-−0.05) |
| Tropical Latin America | 42.5 (28.7-55.7) | 31.2 (19.6-43.2) | −0.27 (−0.38-−0.17) |  | 42.5 (28-56.1) | 31.1 (18.7-44.1) | −0.27 (−0.39-−0.16) |
| North Africa and Middle East | 18.2 (11-26.3) | 16.8 (10.3-24.2) | −0.08 (−0.14-0) |  | 17.9 (10.7-26) | 16.7 (10.2-23.8) | −0.07 (−0.14-0.01) |
| High-income North America | 42.7 (28.5-56.4) | 26 (14.7-37.8) | −0.39 (−0.54-−0.27) |  | 43 (28.5-57) | 27.9 (16.2-40.7) | −0.35 (−0.48-−0.24) |
| Oceania | 9 (5.1-13.9) | 5.1 (2.6-9.1) | −0.43 (−0.58-−0.23) |  | 8.6 (4.9-13.1) | 4.5 (2.4-7.9) | −0.47 (−0.62-−0.28) |
| Central Sub-Saharan Africa | 22.7 (13.4-32.8) | 31.1 (18-44.9) | 0.37 (0.21-0.61) |  | 21.7 (12.6-32) | 30.4 (17.3-44.9) | 0.4 (0.2-0.63) |
| Eastern Sub-Saharan Africa | 18.1 (11.2-25.8) | 13.9 (8.9-19.6) | −0.23 (−0.3-−0.15) |  | 17.8 (10.5-25.9) | 13.1 (8.2-18.9) | −0.26 (−0.33-−0.18) |
| Southern Sub-Saharan Africa | 17.7 (10-26.4) | 18.9 (10.3-29) | 0.07 (−0.13-0.3) |  | 17.3 (9.4-26) | 18.4 (9.9-28.4) | 0.06 (−0.14-0.31) |
| Western Sub-Saharan Africa | 17 (10.3-24.5) | 8 (5-11.8) | −0.53 (−0.58-−0.46) |  | 17.1 (10.4-25) | 7.4 (4.6-11) | −0.57 (−0.62-−0.5) |

**Supplementary Table S13. Global and regional age-specific summary exposure value rates of a diet high in red meat attributable to early-onset colorectal cancer in 1990 and 2019, and its temporal trends from 1990 to 2019, by sex**

| Characteristics | Male | | |  | Female | | |
| --- | --- | --- | --- | --- | --- | --- | --- |
|  | Age-specific SEV rate in 1990 (95% UI) | Age-specific SEV rate in 2019 (95% UI) | ARC (%) (95% UI) |  | Age-specific SEV rate in 1990 (95% UI) | Age-specific SEV rate in 2019 (95% UI) | ARC (%) (95% UI) |
| Overall | 39.7 (32.9-46.5) | 43.1 (37.2-48.6) | 0.08 (0.01-0.17) |  | 40.1 (33.5-46.5) | 42.9 (36.8-48.3) | 0.07 (0-0.15) |
| Socio-demographic index |  |  |  |  |  |  |  |
| High | 72.4 (66.7-77.6) | 72.1 (66-77.6) | 0 (−0.04-0.03) |  | 72.9 (67.4-77.9) | 71.8 (65.2-77.7) | −0.02 (−0.06-0.02) |
| High -middle | 54.3 (45.8-62.7) | 64 (57-69.9) | 0.18 (0.07-0.31) |  | 56 (48-63.7) | 63.4 (56.3-69.5) | 0.13 (0.04-0.24) |
| Middle | 30.6 (21.8-39.7) | 42 (34.7-48.6) | 0.37 (0.18-0.72) |  | 27.7 (20.3-35.3) | 35.2 (28.4-41.6) | 0.27 (0.15-0.47) |
| Low-middle | 15.6 (11.2-20.5) | 20.7 (16.2-25.5) | 0.32 (0.21-0.5) |  | 15.7 (11.6-20.1) | 21 (16.7-25.7) | 0.34 (0.25-0.48) |
| Low | 16 (10.1-21.7) | 17.2 (11-23.2) | 0.07 (0.03-0.13) |  | 15.8 (10.3-21.4) | 17.1 (11.3-23) | 0.08 (0.03-0.13) |
| GBD regions |  |  |  |  |  |  |  |
| High-income Asia Pacific | 33.9 (23.6-44) | 54.7 (46.2-62.4) | 0.62 (0.39-1.05) |  | 32.6 (22.3-42.5) | 45.2 (34.9-54.8) | 0.39 (0.21-0.71) |
| Central Asia | 59 (51.1-66.4) | 62 (53.6-69.8) | 0.05 (−0.01-0.12) |  | 58.7 (50.9-66.1) | 62 (54.1-69.7) | 0.06 (−0.01-0.13) |
| East Asia | 41.9 (29.2-54.8) | 73.5 (62.3-82.4) | 0.76 (0.39-1.4) |  | 42.7 (30.8-55.8) | 74.4 (62.9-83.8) | 0.74 (0.37-1.41) |
| South Asia | 7.4 (4.9-10.4) | 8 (5.1-11) | 0.07 (0.01-0.14) |  | 7 (4.7-9.5) | 7.5 (5-10.5) | 0.08 (−0.03-0.19) |
| Southeast Asia | 16 (9.7-22.1) | 24.9 (17.7-32) | 0.56 (0.38-0.88) |  | 16 (9.7-22.2) | 24.7 (17.3-32) | 0.54 (0.37-0.89) |
| Australasia | 99.9 (99.7-100) | 99.7 (98.6-100) | 0 (−0.01-0) |  | 99.9 (99.6-100) | 99.7 (98.6-100) | 0 (−0.01-0) |
| Caribbean | 28.6 (19.4-37.4) | 29 (19.7-38.1) | 0.01 (−0.06-0.1) |  | 28.3 (19.2-37.1) | 28.4 (19-37.5) | 0 (−0.07-0.08) |
| Central Europe | 63.2 (55-70.9) | 73.1 (65.6-79.8) | 0.16 (0.1-0.24) |  | 63.2 (55.2-70.9) | 72.9 (64.7-79.7) | 0.15 (0.09-0.23) |
| Eastern Europe | 73.6 (66.4-80.3) | 56.6 (45.8-66.5) | −0.23 (−0.34-−0.14) |  | 73.3 (66.1-80) | 56.4 (46-66.4) | −0.23 (−0.33-−0.14) |
| Western Europe | 86.6 (82.7-90.2) | 82.5 (76.8-87.3) | −0.05 (−0.08-−0.02) |  | 86.5 (82.3-90.2) | 82.4 (77-87.3) | −0.05 (−0.08-−0.02) |
| Andean Latin America | 27.7 (19.1-36) | 38.8 (29.4-47.8) | 0.4 (0.24-0.66) |  | 27.6 (19-36.2) | 35.6 (26-45) | 0.29 (0.18-0.48) |
| Central Latin America | 44.2 (34.6-53.6) | 47.5 (37.9-57) | 0.07 (−0.01-0.18) |  | 44 (34-53.3) | 46.6 (37.3-55.7) | 0.06 (−0.02-0.17) |
| Southern Latin America | 85.7 (82.1-89) | 93.4 (89-96.8) | 0.09 (0.05-0.14) |  | 85.5 (82-88.9) | 93.6 (89.5-96.9) | 0.1 (0.06-0.14) |
| Tropical Latin America | 58 (47.7-67.3) | 89.9 (82.8-95.2) | 0.55 (0.36-0.83) |  | 57.8 (47.8-67.1) | 89.8 (82.8-95.2) | 0.55 (0.36-0.85) |
| North Africa and Middle East | 25.3 (16.4-33.9) | 25.1 (16.2-33.6) | −0.01 (−0.05-0.04) |  | 24.6 (15.7-33.2) | 24.4 (15.4-33.2) | −0.01 (−0.05-0.03) |
| High-income North America | 84.6 (79.1-89.4) | 81.8 (73.2-88.7) | −0.03 (−0.11-0.04) |  | 84.4 (78.9-89.4) | 80.2 (70.2-87.9) | −0.05 (−0.15-0.03) |
| Oceania | 35.6 (24.7-46.6) | 30.7 (20-42.3) | −0.14 (−0.31-0.06) |  | 35.6 (24.9-47.3) | 30.3 (19.8-42.2) | −0.15 (−0.32-0.04) |
| Central Sub-Saharan Africa | 15.6 (10.4-21.1) | 15 (10.3-20) | −0.04 (−0.1-0.05) |  | 15.2 (9.9-20.6) | 15.4 (10.5-20.5) | 0.01 (−0.06-0.11) |
| Eastern Sub-Saharan Africa | 18.5 (11.5-25.5) | 19.2 (11.6-26.6) | 0.04 (−0.02-0.09) |  | 19.1 (11.8-26.5) | 20.1 (12.6-27.9) | 0.05 (−0.06-0.16) |
| Southern Sub-Saharan Africa | 40.9 (30.7-50.5) | 46.1 (35.6-55.9) | 0.13 (0-0.3) |  | 40.6 (30.6-50.3) | 45.3 (33.9-55.6) | 0.11 (−0.01-0.27) |
| Western Sub-Saharan Africa | 18.1 (11.1-25.1) | 20.2 (12.5-27.9) | 0.12 (0.06-0.19) |  | 18.5 (11.4-25.5) | 20 (12.6-27.6) | 0.09 (0.03-0.16) |

**Supplementary Table S14. Global and regional age-specific summary exposure value rates of a diet high in processed meat attributable to early-onset colorectal cancer in 1990 and 2019, and its temporal trends from 1990 to 2019, by sex**

| Characteristics | Male | | |  | Female | | |
| --- | --- | --- | --- | --- | --- | --- | --- |
|  | Age-specific SEV rate in 1990 (95% UI) | Age-specific SEV rate in 2019 (95% UI) | ARC (%) (95% UI) |  | Age-specific SEV rate in 1990 (95% UI) | Age-specific SEV rate in 2019 (95% UI) | ARC (%) (95% UI) |
| Overall | 27.9 (18.3-39.5) | 27.5 (17-41.7) | −0.01 (−0.1-0.07) |  | 30.5 (20.9-41) | 30.4 (19.7-44.3) | 0 (−0.09-0.08) |
| Socio-demographic index |  |  |  |  |  |  |  |
| High | 66.9 (44.4-86.1) | 67 (48.3-83) | 0 (−0.05-0.1) |  | 74.4 (53.4-92.1) | 78.8 (62.3-92.8) | 0.06 (0-0.18) |
| High -middle | 33.6 (24.2-45.6) | 31.8 (19.1-49.2) | −0.05 (−0.22-0.08) |  | 40.6 (31-51.6) | 40.3 (25.6-56.9) | −0.01 (−0.18-0.13) |
| Middle | 11.1 (5.8-20.7) | 16.3 (8.3-30.5) | 0.46 (0.25-0.67) |  | 12 (6.1-23.6) | 18.7 (9.6-34) | 0.56 (0.29-0.85) |
| Low-middle | 15.1 (7.9-27.8) | 19 (9.8-33.1) | 0.26 (0.08-0.45) |  | 15.2 (8.3-26.7) | 20.7 (11.8-33.6) | 0.36 (0.14-0.59) |
| Low | 20.7 (10.5-37) | 22.3 (11.8-39.2) | 0.08 (−0.04-0.24) |  | 25.4 (13.9-42.2) | 28.7 (16.3-46.1) | 0.13 (0.02-0.29) |
| GBD regions |  |  |  |  |  |  |  |
| High-income Asia Pacific | 54.3 (28.4-78.9) | 59.3 (31.7-84.9) | 0.09 (−0.04-0.28) |  | 71.6 (46.2-94.6) | 77.3 (52.7-97.2) | 0.08 (−0.02-0.26) |
| Central Asia | 48.1 (28-71.7) | 49.2 (28.7-72.8) | 0.02 (−0.13-0.23) |  | 55.4 (33.8-77.6) | 57.3 (34.2-79.8) | 0.03 (−0.08-0.18) |
| East Asia | 8.7 (3.9-19.2) | 16.4 (7-34.7) | 0.89 (0.22-1.6) |  | 9.5 (4.2-20) | 19.4 (8.6-38.6) | 1.05 (0.42-1.94) |
| South Asia | 15.3 (7.9-29.1) | 18.8 (10-33.7) | 0.23 (0.07-0.46) |  | 13.4 (7.1-25.4) | 17.4 (9.6-32.1) | 0.3 (0.1-0.57) |
| Southeast Asia | 6.6 (3.1-15.5) | 9 (4.2-19.8) | 0.36 (0.09-0.62) |  | 7.8 (3.7-18) | 11.1 (5.3-22.8) | 0.43 (0.06-0.73) |
| Australasia | 68.3 (41.5-93) | 75.6 (48.5-98.9) | 0.11 (0.02-0.29) |  | 73.9 (46.6-97.9) | 78.1 (52-99.7) | 0.06 (0.01-0.18) |
| Caribbean | 15 (6.5-32) | 15.9 (7.1-32.1) | 0.05 (−0.1-0.27) |  | 16.9 (7.1-34.5) | 17.9 (8.1-36.8) | 0.06 (−0.11-0.28) |
| Central Europe | 40.9 (21.6-63.8) | 56 (33-77.2) | 0.37 (0.14-0.7) |  | 52.2 (24.6-79.2) | 61 (31.7-86.8) | 0.17 (0.03-0.43) |
| Eastern Europe | 79.6 (70.9-88) | 62.7 (39.7-84.6) | −0.21 (−0.43-−0.03) |  | 82 (75.6-88.8) | 66.7 (43-88.7) | −0.19 (−0.43-0.02) |
| Western Europe | 75.2 (51.2-94.7) | 78.6 (56.8-97.1) | 0.05 (0.02-0.1) |  | 78.3 (56-96.6) | 81.7 (61.6-98.4) | 0.04 (0.01-0.1) |
| Andean Latin America | 10.7 (5.3-21) | 13.3 (6-26.1) | 0.24 (−0.08-0.57) |  | 10.5 (5-20.5) | 13.2 (5.8-28) | 0.25 (−0.06-0.56) |
| Central Latin America | 19.7 (9.4-37.3) | 22.7 (10.9-41) | 0.15 (−0.09-0.49) |  | 22.1 (11-40) | 25.1 (12.4-43.7) | 0.13 (−0.1-0.44) |
| Southern Latin America | 47.9 (28.7-69.7) | 59.3 (36.9-82.6) | 0.24 (0.07-0.52) |  | 53.9 (32.4-77.4) | 66.6 (42.9-90) | 0.24 (0.07-0.49) |
| Tropical Latin America | 16.7 (7.2-35.5) | 24.4 (11.9-44.2) | 0.46 (0.16-0.99) |  | 19.1 (8.6-37.2) | 28.6 (14.5-49) | 0.5 (0.17-1.18) |
| North Africa and Middle East | 10.7 (3.6-26.2) | 12.6 (4.2-31.3) | 0.18 (0.03-0.36) |  | 13 (5.6-28.3) | 16 (7-33.7) | 0.24 (0.04-0.54) |
| High-income North America | 77.3 (53.6-97.1) | 84.7 (66.5-98.2) | 0.1 (0.01-0.28) |  | 78.4 (55.4-96.5) | 85.9 (70.7-98) | 0.1 (0.01-0.32) |
| Oceania | 9.6 (3.9-20.7) | 9.8 (4.1-21.2) | 0.02 (−0.19-0.29) |  | 10.7 (4.3-23) | 10.9 (4.5-23.8) | 0.02 (−0.22-0.32) |
| Central Sub-Saharan Africa | 17.6 (3.6-45) | 16.7 (3.4-46.4) | −0.05 (−0.35-0.25) |  | 17.6 (3.6-45) | 16.7 (3.4-46.4) | −0.05 (−0.35-0.25) |
| Eastern Sub-Saharan Africa | 19.3 (8.9-37.7) | 20.4 (9.5-39) | 0.05 (−0.06-0.22) |  | 21 (10-39.6) | 22.4 (10.7-41.7) | 0.07 (−0.06-0.26) |
| Southern Sub-Saharan Africa | 15.9 (6.8-33.2) | 18.8 (8.2-38.2) | 0.18 (0.01-0.47) |  | 18.1 (7.7-37) | 21.5 (10.2-40.7) | 0.19 (0-0.48) |
| Western Sub-Saharan Africa | 31 (16-51.6) | 35.3 (18.9-56.8) | 0.14 (−0.05-0.46) |  | 36.3 (19.6-57.6) | 42.3 (23.7-64.7) | 0.16 (−0.02-0.44) |
